# Spontaneous Intracellular Formation of Covalent Organic Frameworks via Enzyme-Initiated Cascade Reactions to Boost Cancer Radiotherapy

**DOI:** 10.1021/jacs.6c07767

**Published:** 2026-07-24

**Authors:** Qun Guan, Le-Le Zhou, Wei Zhou, Zhiqing Yang, Bichun Chen, Yan-An Li, Ruibing Wang

**Affiliations:** a State Key Laboratory of Mechanism and Quality of Chinese Medicine, Institute of Chinese Medical Sciences, 59193 University of Macau, Taipa, Macao 999078, China; b Department of Pharmaceutical Sciences and MoE Frontiers Science Center for Precision Oncology, University of Macau, Taipa, Macao 999078, China; c School of Chemistry and Pharmaceutical Engineering, Medical Science and Technology Innovation Center, 12589 Shandong First Medical University & Shandong Academy of Medical Sciences, Jinan 250000, China; d Tumor Research and Therapy Center, Shandong Provincial Hospital Affiliated to Shandong First Medical University, Jinan 250021, China; e College of Chemistry, Chemical Engineering and Materials Science, Collaborative Innovation Center of Functionalized Probes for Chemical Imaging in Universities of Shandong, MoE Key Laboratory of Molecular and Nano Probes, 47856 Shandong Normal University, Jinan 250014, China

## Abstract

Tumor radioresistance is a leading cause of radiotherapy failure. This study presents a facile strategy for enhancing tumor radiosensitivity using a covalent organic framework (COF) constructed within cells. Upon cellular internalization, acetamide and iodoacetal precursors undergo hydrolysis to form amine and iodoaldehyde monomers that spontaneously polymerize and crystallize within the lysosomes. This intracellularly generated iodine-rich COF (in situ **UM-113**) possesses an ordered structure that effectively facilitates radiation energy deposition, producing localized reactive oxygen species and amplifying DNA damage. In vitro and in vivo, in situ **UM-113** suppresses tumor growth by triggering both ferroptosis and mitotic catastrophe. This strategy bypasses the complex synthesis and purification steps required for conventional nanomedicine. Thus, in situ COF construction is a promising strategy for enhancing cancer radiotherapy, offering a modular platform for the intracellular synthesis of functional bionanomaterials.

## Graphical abstract

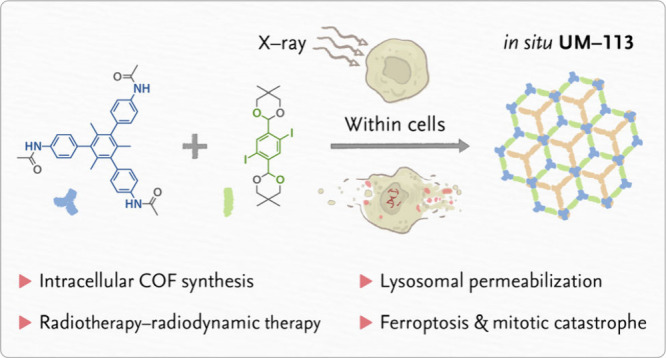

The therapeutic efficacy of radiotherapy, wherein high-energy ionizing radiation is used to damage tumor tissue, is often limited by radioresistance.^1^ Radiosensitizers offer a promising strategy to overcome this limitation by concentrating radiation energy within tumor cells or by diminishing their intrinsic repair capacity, broadening the therapeutic window without increasing the radiation dose, and have significant development potential.^2^

Radiosensitizers used in (pre)­clinical research fall into two main categories. Small-molecule radiosensitizers, e.g., cisplatin and nitroimidazoles, offer advantages such as good stability, convenient administration, and well-defined mechanisms.^3^ However, their clinical utility is limited by rapid clearance, insufficient tumor accumulation, and significant systemic toxicity.^4^ Conversely, nanoradiosensitizers, e.g., metal–organic frameworks, typically comprising high-*Z* elements, exhibit robust radiodynamic effects and prolonged tumor retention.^5^ However, their clinical application is hindered by their complex synthesis and purification, potential instability during storage and transportation, and concerns over metal-induced biotoxicity.^6^

Recently, covalent organic frameworks (COFs), a class of porous crystalline polymers, have emerged as promising radiosensitization platforms owing to their intrinsic biocompatibility and structural tunability.^7^ Nevertheless, their clinical translation faces the aforementioned challenges, even in the absence of metal-related toxicity concerns.^8^

Herein, we report an in situ COF synthesis strategy that integrates the advantages of both small-molecule and nanoscale radiosensitizers. Through the intracellular polymerization of small-molecule precursors, iodine-containing radiosensitizers are directly converted into COF nanoparticles (in situ **UM-113**) within the cells, effectively inhibiting radioresistant tumor growth in synergy with X-ray irradiation (Figure 1). Our results demonstrate the potential of intracellular polymerization to sensitize cells to radiotherapy, establishing a paradigm for in situ nanotherapeutics.

**Figure 1:**
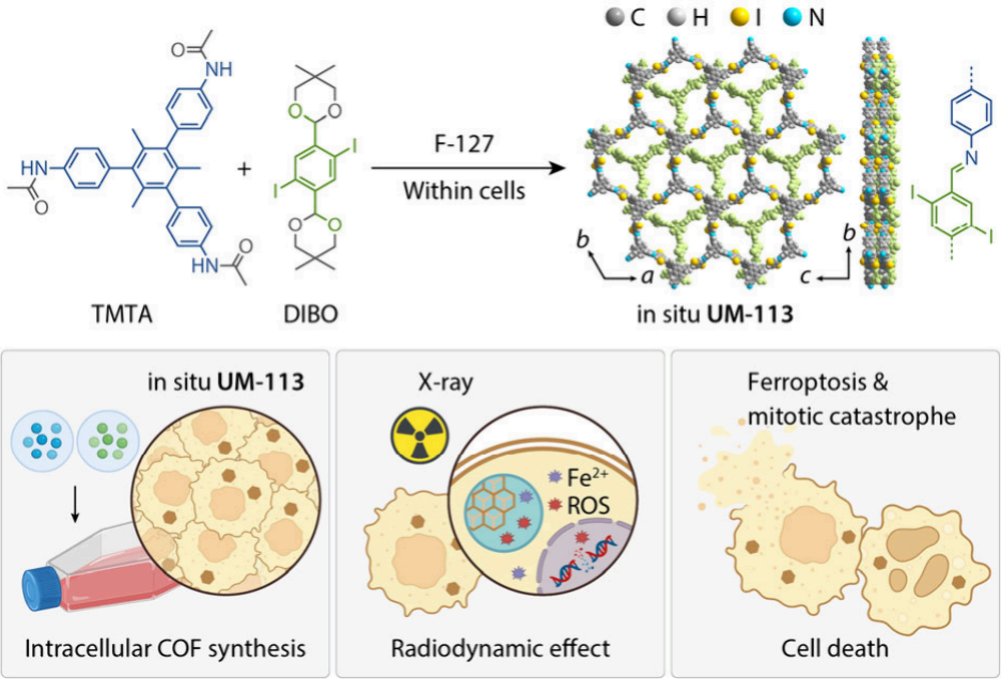
Spontaneous formation of intracellular COFs for enhancing cancer radiotherapy.

Precise control of the crystallization kinetics required to synthesize highly crystalline COFs.^9^ A promising strategy to achieve such control involves protecting groups that temporarily block reactive sites, thereby enabling triggered monomer release under specified conditions.^10^ We designed an enzyme-initiated cascade reaction using two protected building blocks 2,4,6-tris­(4-acetamidophenyl)-1,3,5-trimethylbenzene (TMTA) and 2,2′-(2,5-diiodo-1,4-phenylene)­bis­(5,5-dimethyl-1,3-dioxane) (DIBO) (Figures S1 and S2). In a typical synthesis, TMTA and DIBO were incubated with esterase in an acetate buffer (pH 8.8) to selectively hydrolyze the acetamide groups of TMTA and release reactive amines. Subsequent acidification with acetic acid to pH 4.7 removed the neopentyl glycol acetal protecting groups from DIBO, generating a dialdehyde that immediately underwent imine polymerization with the free amine. Time-dependent ultraviolet–visible spectroscopy showed gradual imine formation, with the characteristic absorption at 425 nm increasing over 24 h (Figure 2a). Control experiments conducted with albumin instead of esterase, or at pH 6.5 and 7.4, showed little color change (Figure S3). This cascade process was further validated through stepwise model reactions (Figures S4–S6).

The reaction was quenched with triethylamine to afford the yellow solid **UM-113**. Powder X-ray diffraction (PXRD) confirmed its crystallinity, with an experimental pattern that closely matched the AB-stacking model based on the **hcb** topology (Figures 2b and S7).^11^ Pawley refinement confirmed the *P*6_3_ space group, giving cell parameters *a* = *b* = 37.22 Å, *c* = 6.94 Å, α = β = 90°, and γ = 120° (Tables S1 and S2). The permanent porosity of **UM-113** was confirmed by type-I N_2_ adsorption–desorption isotherms at 77 K, which revealed a pore size of 1.3 nm, consistent with the AB-stacking model (Figure S8).^12^ Spectroscopic analyses verified the formation of C=N linkages and the retention of C–I groups (Figures S9 and S10). Thermogravimetric analysis indicated high thermal stability up to 300 °C with no residual guests (Figure S11). Scanning and transmission electron microscopy (TEM) revealed that **UM-113**, with a particle size of approximately 100 nm (Figure S12). Conversely, direct polymerization of the unprotected monomers 2,4,6-tris­(4-aminophenyl)-1,3,5-trimethylbenzene and 2,5-diiodoterephthalaldehyde in methyl acetate yielded **UM-114­(AA′)**, featuring an AA′-stacking structure (Figure S13). This material comprised micron-sized particles that readily aggregated in Dulbecco’s phosphate-buffered saline (DPBS). Moreover, using alternative solvents (e.g., nitriles and dichloromethane) only produced amorphous products (Figure S14).

**Figure 2:**
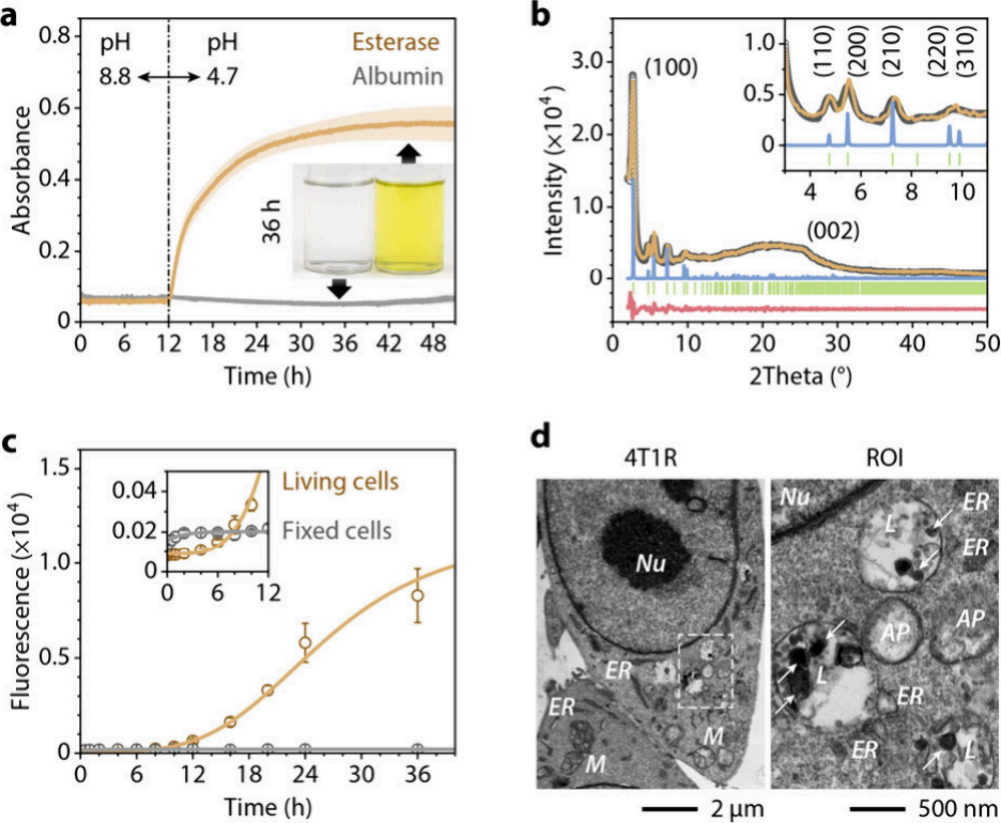
COF synthesis in water and inside 4T1R cells. (a) Kinetics of esterase- and acid-catalyzed cascade synthesis of **UM-113** from TMTA (50 μM) and DIBO (75 μM) with esterase (50 U/L). (b) PXRD analysis. (c) Intracellular fluorescence intensity measured by flow cytometry of 4T1R cells treated with TMTA (4.0 μM), DIBO (6.0 μM), and F-127 (0.04 wt %) for 0–36 h. Data are expressed as mean ± SD and fitted with the Logistic model, *n* = 4 biological replicates. (d) TEM images of the treated 4T1R cells showing in situ **UM-113** nanoparticles (indicated by arrows) within lysosomes.

Lysosomal contents, with a pH of 4.5–5.0, provide a favorable catalytic environment for intracellular COF synthesis.^13^ Considering the hydrophobicity and biosafety of TMTA and DIBO, they were dissolved in F-127 and co-incubated with radioresistant 4T1R cells (Figures S15 and S16). F-127 facilitates cellular uptake by improving solubility and increasing cytomembrane permeabilization and pinocytosis (Figure S17). Flow cytometry revealed that intracellular fluorescence remained approximately unchanged for the first 6 h and then gradually increased, indicating a cascade reaction involving acetamide hydrolysis and imine polymerization occurred inside the cells (Figures 2c, S18, and S19). This process was cell-dependent, as no fluorescence change was observed in methanol-fixed cells under identical conditions. After 24 h of co-incubation, confocal microscopy showed green punctate fluorescence that colocalized with lysosomes, with a high Pearson’s colocalization coefficient of 0.82 ± 0.03 (Figure S20). TEM imaging further localized the polymerization, revealing abundant spherical crystalline nanoparticles with an interplanar spacing of 3.5 Å exclusively within lysosomes (Figure 2d and S21). Small-angle PXRD analysis of the gel isolated from the cells revealed a diffraction peak at approximately 2.7°, confirming its crystallinity (Figure S22). Notably, in situ **UM-113** was also synthesized in healthy tissue cells, with slower reaction kinetics (Figures S23 and S24). These results demonstrate the spontaneous synthesis of in situ **UM-113** within the lysosomes of living cells.

The highly crystalline and periodically arranged iodine atoms within **UM-113** endow it with strong X-ray attenuation capacity, surpassing those of the commercial CT contrast agents iopromide and iohexol (Figure S25). Hence, **UM-113** was hypothesized to exhibit radiodynamic properties by promoting the radiolysis of water and oxygen.^14^ Under 2–10 Gy X-ray irradiation in DPBS, **UM-113** significantly amplified the fluorescence of various reactive oxygen species (ROS) probes, including hydroxyphenyl fluorescein (HPF), dihydroethidium (DHE), bis­(benzyl boronic ester)­fluorescein (FBBBE), and singlet oxygen sensor green (SOSG), confirming the generation of ·OH, ·O_2_^–^, H_2_O_2_, and ^1^O_2_, respectively (Figures 3 and S26–S29).^15^ Linear model fitting further demonstrated that these fluorescence enhancements were 2.9-, 2.2-, 4.9-, and 1.2-fold higher, respectively, than those observed in the DIBO control. Consistently, after 4 Gy X-ray irradiation, in situ **UM-113** increased the intracellular ROS levels more effectively than DIBO (Figure S30).

**Figure 3:**
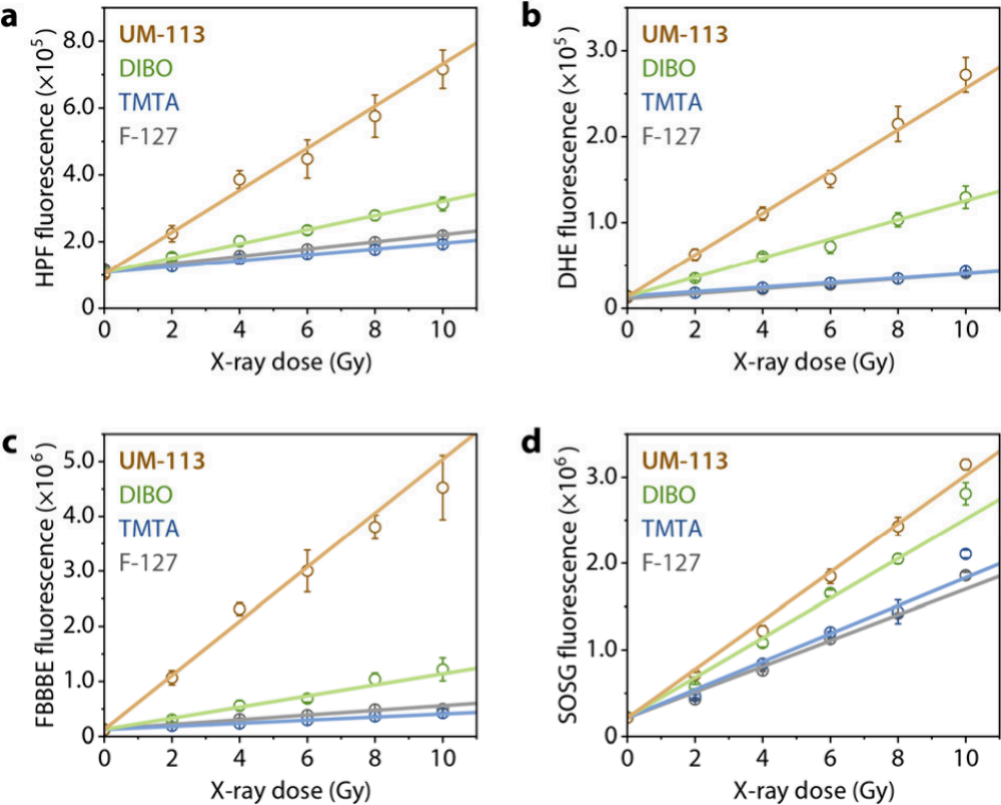
Detection of X-ray-induced ^•^OH (a), ^•^O_2_^–^ (b), H_2_O_2_ (c), and ^1^O_2_ (d) generation in DPBS using HPF, DHE, FBBBE, and SOSG fluorescence probes, respectively, in the presence of F-127 (1.0 wt %), TMTA (100 μM), DIBO (150 μM), or **UM-113** (300 μM, iodine equiv). Data are expressed as mean ± SD and fitted with the linear model, *n* = 4 independent experiments.

The radiosensitizing effects of in situ **UM-113** were investigated by pretreating 4T1R cells with TMTA and/or DIBO for 24 h to induce in situ **UM-113** synthesis, then exposing them to 4 Gy X-ray irradiation. Thirty minutes postirradiation, immunofluorescence staining for γH2AX, a marker of DNA double-strand breaks, revealed considerably more nuclear foci in co-treated cells than in cells pretreated with DIBO alone (Figure 4a).^16^ The alamarBlue cell viability assays showed that 24 and 72 h postirradiation, in situ **UM-113** reduced the cell viability to 61% and 12%, respectively, compared to 73% and 31% with DIBO alone, indicating an enhanced radiodynamic effect (Figure 4b). Consistently, colony formation assays demonstrated that in situ **UM-113** treatment significantly reduced both the number and size of the colonies compared to DIBO alone (Figures 4c and S31). These results suggest that in situ **UM-113** achieves sustained inhibition and killing of 4T1R tumor cells via radiodynamic therapy.

**Figure 4:**
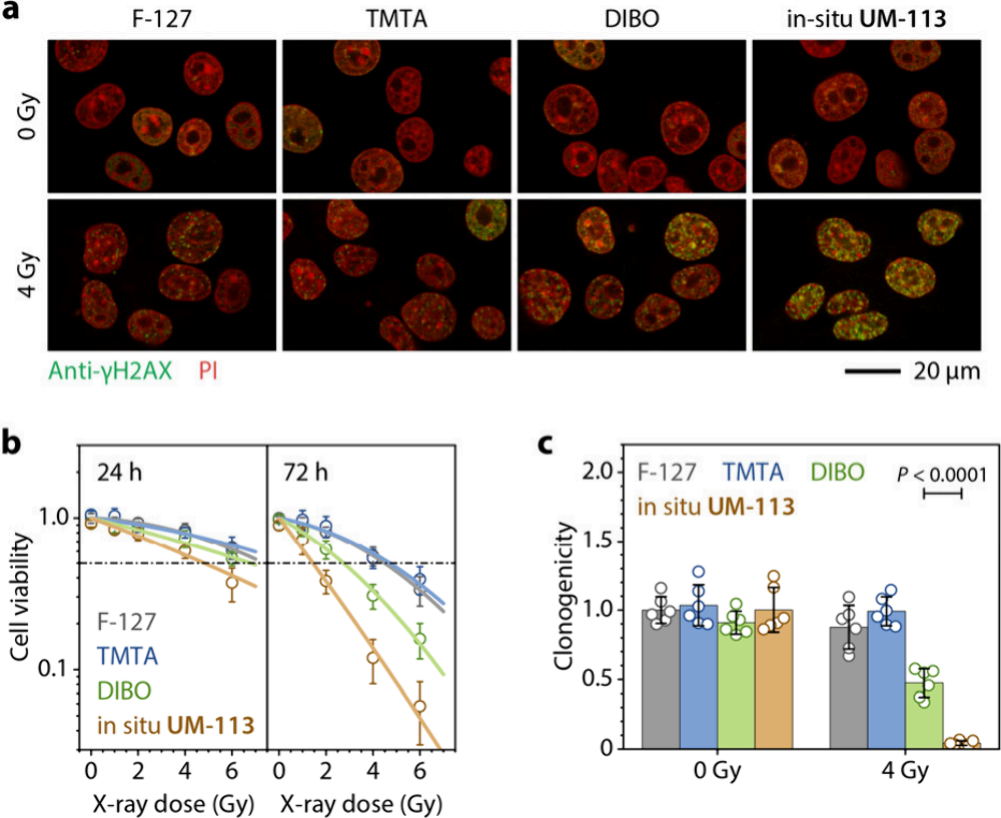
In vitro radiotherapy induced by in situ **UM-113**. 4T1R cells are pretreated with TMTA (4.0 μM), DIBO (6.0 μM), or/and F-127 (0.04 wt %) for 24 h and irradiated with X-rays (0–6 Gy). (a) γH2AX immunofluorescence staining. (b) Cell viability assays. (c) Clonogenic assays. Data are expressed as mean ± SD and fitted with the linear quadratic model, *n* = 6 technical replicates. Statistical significance is calculated by two-way ANOVA with Tukey’s post hoc test.

Temporal analysis of cell death revealed a multiphasic response following treatment with in situ **UM-113** and X-ray irradiation. At 24 h, although the cells exhibited no apparent morphological changes, significant biochemical alterations indicated lysosome-originated ferroptosis characterized by lysosomal permeabilization, leading to the release of Fe^2+^ into the cytoplasm, which in turn triggered lipid peroxidation and ROS accumulation (Figure S32).^17^ This oxidative stress was corroborated by glutathione depletion, upregulation of *Ptgs2* mRNA, and increased malondialdehyde levels. Notably, despite the partial depolarization of the mitochondrial membrane potential and minor phosphatidylserine externalization, the absence of caspase 3 activation suggests that apoptosis is not the primary cell death mechanism at this stage (Figure S33). At 72 h, the cells exhibited hallmarks of mitotic catastrophe, marked by nuclear abnormalities (e.g., micronuclei and multinucleation) and cytoskeletal disorganization (Figure S34). Increased COX2 expression and senescence-associated β-galactosidase activity indicated that cells surviving the initial ferroptotic stress underwent cellular senescence and lost their proliferative capacity upon prolonged culture.^18^ Conversely, DIBO-sensitized radiotherapy induced only mild ferroptosis at 24 h and did not trigger senescence. Thus, in situ **UM-113** couples acute DNA damage with ferroptotic oxidative damage, ultimately leading to delayed mitotic catastrophe and senescence, thereby ensuring long-term antitumor efficacy.

Intratumoral COF synthesis was investigated by co-injecting TMTA and DIBO directly into 4T1R tumors in BALB/c mice. After 24 h, the dissociated tumor cells exhibited concentration-dependent fluorescence corresponding to in situ **UM-113** (Figures 5a and S35). The formation of in situ **UM-113** deposited iodine within the tumor, significantly slowing down its clearance (Figure S36). TEM imaging revealed electron-dense crystalline structures exclusively within the lysosomes of the co-injection group, which were completely absent in the F-127 control group, further supporting the intralysosomal formation of in situ **UM-113** (Figures 5b and S37).

**Figure 5:**
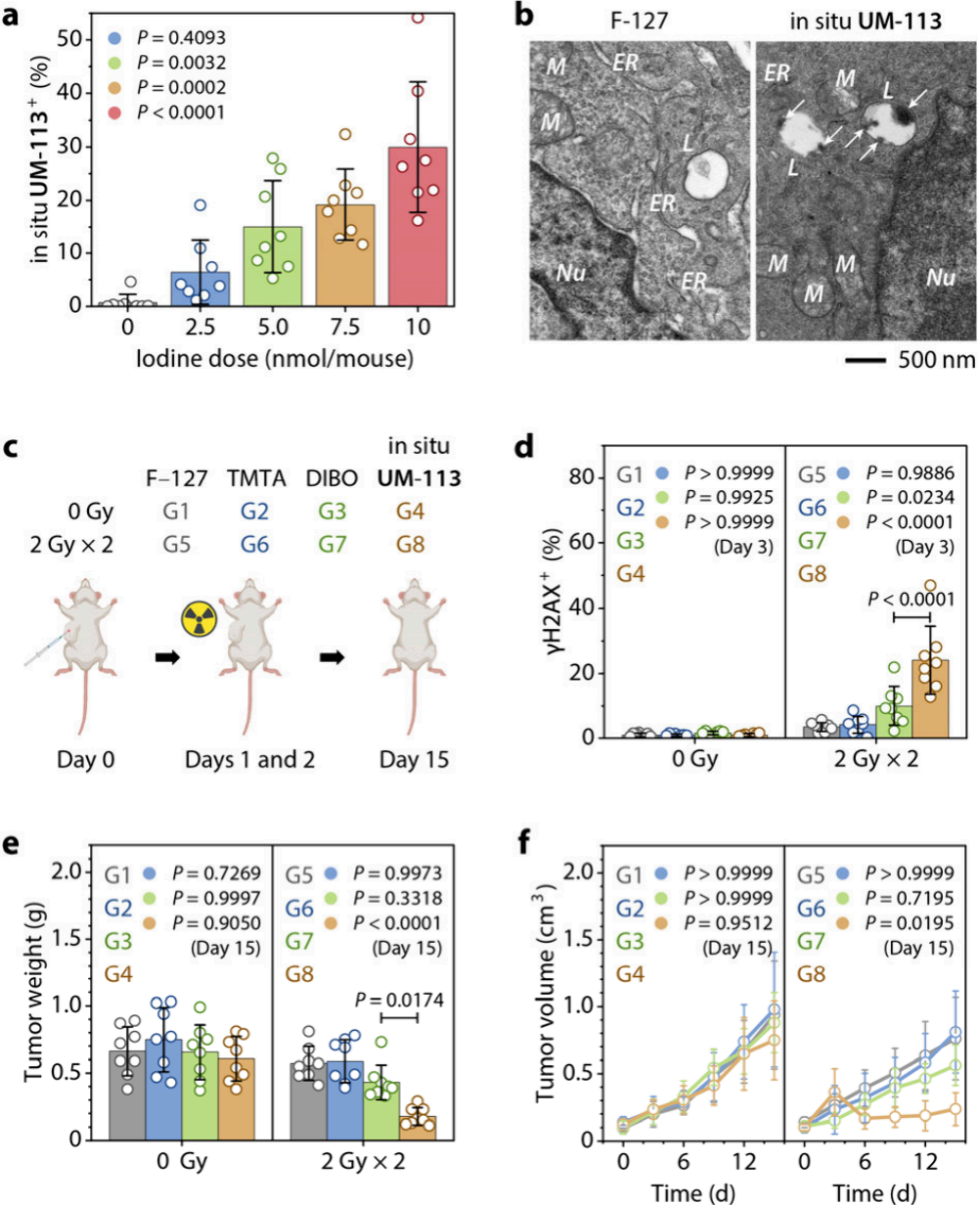
In vivo therapy induced by in situ **UM-113**. (a) Percentage of in situ **UM-113**-positive cancer cells in tumor tissue 24 h after co-injection of F-127, TMTA, and DIBO. (b) TEM images of tumors showing in situ **UM-113** nanoparticles (indicated by arrows) within lysosomes. (c) In vivo treatment protocol. (d) Percentage of γH2AX-positive cells in excised tumor tissue on day 3. (e) Tumor weight on day 15. (f) Tumor growth curves. Data are expressed as mean ± SD, *n* = 8 mice/group. Statistical significance is determined using one-way ANOVA with Dunnett’s post hoc test (a), two-way ANOVA with Tukey’s post hoc test (d, e), and two-way repeated measures ANOVA with Geisser–Greenhouse correction and Tukey’s post hoc tests (f).

The therapeutic efficacy was evaluated in vivo by administering 4T1R tumor-bearing mice with an intratumoral co-injection of TMTA (2.50 nmol/mouse) and DIBO (3.75 nmol/mouse), followed by 2 Gy/d X-ray irradiation at 24 and 48 h postinjection (Figure 5c). Consistent with the in vitro findings, in situ **UM-113**-sensitized radiotherapy resulted in a 7.0-fold increase in intratumoral γH2AX expression compared to radiotherapy alone, indicating enhanced DNA damage (Figures 5d and S38). At the treatment end point, only the group receiving in situ **UM-113**-sensitized radiotherapy showed significant tumor suppression, with the tumor weights reduced to 27% of those of the control group (Figures 5e and S39). Neither in situ **UM-113** alone nor DIBO-sensitized radiotherapy produced notable antitumor effects (Figures 5f and S40). Histological analysis of tumors treated with in situ **UM-113**-sensitized radiotherapy revealed features consistent with ferroptosis and necrosis, including cytoplasmic vacuolization and cytomembrane rupture (Figure S41). Immunohistochemical staining further demonstrated reduced Ki67 expression, suggesting impaired tumor cell proliferation.^19^ Notably, the mouse body weight remained stable throughout the treatment period, and no obvious histopathological damage was observed in the major organs, suggesting acceptable systemic safety (Figures S42 and S43). Moreover, increasing the intratumoral injection dose led to tumor eradication, whereas intravenous injection did not achieve satisfactory efficacy (Figures S44 and S45).

This study presented a strategy for overcoming tumor radioresistance by in situ COF synthesis via enzyme-initiated cascade reactions inside cancer cells. The as-formed COF nanoparticles enhance the localized radiation energy deposition and amplify DNA damage and ROS production, thereby suppressing tumor growth. Although the intratumoral administration may limit further clinical translation, intracellular COF construction combines the advantages of small-molecule drugs and nanomedicines, offering a promising strategy for enhanced oncotherapy and advancing the in situ synthesis of functional bionanomaterials within living cells.^20^

## Supplementary Material



## Notes

Atomic coordinates of **UM-113** and **UM-114­(AA′)** (Tables S1 and S2); supplementary data for COF characterization, in vitro results, and animal experiments (Figures S1–S45); and experimental procedures (PDF)
