# Supplementary material for: Spontaneous Intracellular Formation of Covalent Organic Frameworks via Enzyme-Initiated Cascade Reactions to Boost Cancer Radiotherapy
Source: J Am Chem Soc. 2026 Jul 24;148(30):31634–40. doi: 10.1021/jacs.6c07767 (PMC13450384; doi:10.1021/jacs.6c07767)
Supplement: Supplementary file 1 [file ja6c07767_si_001.pdf]

Supporting Information

Spontaneous Intracellular Formation of Covalent Organic Frameworks via Enzyme-Initiated Cascade Reactions to Boost Cancer Radiotherapy

Qun Guan,[a,b†] Le-Le Zhou,[a,c†] Wei Zhou,[d†] Zhiqing Yang,[a] Bichun Chen,[a] Yan-An Li,[e] Ruibing Wang[a,b*]

[a] State Key Laboratory of Mechanism and Quality of Chinese Medicine, Institute of Chinese Medical Sciences, University of Macau, Taipa, Macao 999078, China. *E-mail: rwang@um.edu.mo

[b] Department of Pharmaceutical Sciences and MoE Frontiers Science Center for Precision Oncology, University of Macau, Taipa, Macao 999078, China.

[c] School of Chemistry and Pharmaceutical Engineering, Medical Science and Technology Innovation Center, Shandong First Medical University & Shandong Academy of Medical Sciences, Jinan 250000, China.

[d] Tumor Research and Therapy Center, Shandong Provincial Hospital Affiliated to Shandong First Medical University, Jinan 250021, China.

[e] College of Chemistry, Chemical Engineering and Materials Science, Collaborative Innovation Center of Functionalized Probes for Chemical Imaging in Universities of Shandong, MoE Key Laboratory of Molecular and Nano Probes, Shandong Normal University, Jinan 250014, China.

† Q.G., L.-L.Z., and W.Z. contributed equally.

1. Experimental materials

Unless otherwise noted, all solvents and reagents, e.g., 2,5-diiodoterephthalaldehyde (LeYan, Cat# 1449992), 2,4,6-tris(4-aminophenyl)-1,3,5-trimethylbenzene (Jilin Chinese Academy of Sciences – Yanshen Technology, Cat# YSZC974), and Pluronic F-127 (Sigma–Aldrich, Cat# P2443), were used as received.

2. Experimental instrumentations

Supercritical CO_2 drying was performed using a Tousimis Samdri-PVT-3D critical point dryer. Damp solids were contained in folded filter paper secured with a staple and dried with a 15 min purge time and 15 min equilibration time after heating.

Single crystal X-ray diffractometry was performed on an Agilent SuperNova CCD-based diffractometer with Cu *K*α radiation (λ = 1.54184 Å). A suitable single crystal was selected and mounted onto thin glass fibers. Crystal data were collected at 208 K. The structure was solved by a combination of direct methods with subsequent difference Fourier syntheses and refined by a full matrix least-squares technique based on F^2 using the SHELXL software package. All the non-hydrogen atoms were refined anisotropically. The hydrogen atoms were generated geometrically.

Powder X-ray diffraction (PXRD) patterns were obtained on a Rigaku SmartLab SE X-ray powder diffractometer with Cu *K*α line focused radiation (λ = 1.54184 Å) in the range of 2θ = 2.00°–50.00° (or 2.00°–10.00°) at a step size of 0.01° and a scanning rate of 5.00°/min (or 0.50°/min) in a continuous mode. Powder samples were placed directly on zero-background sample holders and flattened using glass microscope slides.

Liquid-state ^{1}H and ^{13}C nuclear magnetic resonance (NMR) spectra were recorded on a Bruker Avance III 400 MHz NMR spectrometer. Chemical shifts were reported as δ values relative to tetramethylsilane as an internal reference compound. The ^{1}H and ^{13}C NMR spectra averaged 16 and 1024 scans, respectively.

Cross-polarization magic-angle spinning (MAS) solid-state ^{13}C NMR spectra were recorded on a Bruker Avance III HD 400 MHz NMR spectrometer with a 4 mm double-resonance MAS probe. Each spectrum represented an average of 1200 scans.

N_2 adsorption–desorption isotherms were collected by a Micromeritics ASAP 2460 surface area and porosity analyzer at 77 K. The samples were degassed at 120°C for 8 h using a Micromeritics VacPrep degassing station before measurements. The Brunauer–Emmett–Teller (BET) equation was used to calculate the specific surface areas. The pore size distribution was derived from nitrogen measurements using the non-local density functional theory model.

Fourier transform infrared (FTIR) spectra were obtained on a Thermo Scientific Nicolet iS50 FTIR spectrometer

equipped with a diamond attenuated total reflection (ATR) module between 4000–400 cm^{-1}. Each spectrum represented an average of 128 scans.

Ultraviolet–visible (UV–vis) absorption spectra were recorded as required on the three instruments described below: (1) Shimadzu UV-2700 double-beam UV–vis spectrophotometer equipped with a 10 mm quartz cuvette; (2) Thermo Scientific NanoDrop One microvolume UV–vis spectrophotometer; (3) Molecular Devices SpectraMax M2 multimode microplate reader equipped with a temperature control system.

Fluorescence spectra were recorded with the following two devices as required: (1) Edinburgh FLS1000 photoluminescence spectrometer equipped with a 450 W Xe arc lamp; (2) Hitachi F-7000 fluorescence spectrophotometer; (3) Molecular Devices SpectraMax M2 multimode microplate reader equipped with a temperature control system.

Raman spectrum was obtained by a LabRAM HR Evolution Raman spectrometer with a 532 nm laser.

X-ray photoelectron spectroscopy (XPS) was performed on a Thermo Fisher Scientific ESCALAB 250Xi XPS system equipped with a monochromatic Al *K*α radiation source (1486.6 eV). The C 1s signal located at 284.8 eV from adventitious carbon was used as binding energy reference. The Shirely baseline was subtracted from the overall signal data before data deconvolution.

Inductively coupled plasma–mass spectrometry (ICP–MS) measurements were carried out on a Thermo Scientific iCAP RQ ICP–MS system. The standard solutions were obtained from Reference Materials Research Center of Shandong Metallurgical Research Institute (Jinan, China). Before each analysis, the external standard method was used to establish a work curve.

Mass spectrometry analysis was performed using the following 4 devices as appropriate: (1) Bruker maXis ultrahigh-resolution time-of-flight triple quadrupole mass spectrometer; (2) Agilent 6530 quadrupole time-of-flight liquid chromatography–mass spectrometry system; (3) Agilent 7250 quadrupole time-of-flight gas chromatography–mass spectrometer; (4) Thermo Scientific Q Exactive Plus hybrid quadrupole-Orbitrap mass spectrometer. High-resolution mass spectrum (HRMS) was reported as mass per charge ratios (m/z).

Transmission electron microscopy (TEM) images were recorded on an FEI Talos F200X high-resolution scanning transmission electron microscope. Before measurement, the samples were prepared in methanol by sonicating the material for 5 min, followed by application on a copper TEM grid, and air-dried at room temperature.

Scanning electron microscopy (SEM) images were recorded using a Zeiss Supra 55 instrument. Before measurement, the SEM samples were prepared by depositing a diluted methanol suspension onto silicon wafers (approximately 3 mm × 3 mm), followed by air drying and coating with a thin layer of Pt to increase the contrast.

Thermogravimetric analysis (TGA) was performed with a Mettler Toledo TGA/DSC3+ thermogravimetric analyzer. Approximately 3.0 mg of dried sample was analyzed from room temperature to 950°C at a heating rate of 10°C/min under a N_2 atmosphere.

Flow cytometric analysis was performed using the following two devices as appropriate: (1) BD FACSAria Fusion cell sorter equipped with 405, 488, 561, and 640 nm lasers; (2) Beckman Coulter CytoFLEX flow cytometer equipped with 405, 488, and 638 nm lasers. FACSFlow sheath fluid (BD Biosciences, Cat# 342003) and CytoFLEX sheath fluid (Beckman Coulter, Cat# B51503) were used in flow cytometric analysis. For live cell analysis, unless otherwise noted, cells were maintained in HBSS (Gibco, Cat# C14175500CP) supplemented with HEPES (25 mM, pH 7.3; VivaCell, Cat# C3544-0100) and fetal bovine serum (2.0 vol%; Gibco, Cat# A5256701). For fixed cell analysis, unless otherwise noted, cells were maintained in DPBS (Gibco, Cat# C14190500CP).

X-ray irradiation was performed using a Varian Trilogy linear accelerator. For cell radiotherapy, cells were placed on a solid water slab (30 cm × 30 cm × 1 cm), and 6 MV X-rays were delivered along the central axis through a 40 cm × 40 cm square open field at a dose rate of 5 Gy/min. Prior to irradiating tumor-bearing mice, anesthesia was induced with isoflurane (RWD Life Science, Cat# R510-22-10). The tumor was positioned within a narrow 20 cm × 1 cm open field and covered with a 1.0 cm thick tissue compensation membrane. 6 MV X-rays were then delivered along the central axis at a dose rate of 5 Gy/min.

Computed tomography (CT) was performed using a Siemens Somatom Force dual-source CT scanner (Siemens Healthcare, Germany). For sample preparation, samples were dispersed in DPBS (Gibco, Cat# C14190500CP), transferred to polypropylene centrifuge tubes, and fixed on a plexiglass sample holder prior to image acquisition. CT scans were acquired with a fixed tube voltage of 70 kV. Multi-planar reformatted images were generated using a dedicated reconstruction algorithm with a strength level of 5, the Qr40 kernel, and a slice thickness of 0.5 mm.

Laser scanning confocal fluorescence images of cells were acquired using a Leica Stellaris 5 confocal laser scanning microscope equipped with 405 nm and supercontinuum white light (operating at 85% intensity) lasers using the 20× and 63× objective lens at 23°C. The 2D imaging was performed at the focal plane of maximum fluorescence intensity with a scan speed of 400 Hz. To minimize photodamage to the samples, regions of interest were initially identified using transmitted light before fluorescence imaging. Four-chamber glass-bottom dishes (Cellvis, Cat# D35C4-20-0-N) and 8-chamber slides (Cellvis, Cat# C8-1.5H-N) were used for cell culture in experiments. For live cell imaging, cells were maintained in FluoroBrite DMEM (Gibco, Cat# A1896701) supplemented with HEPES (25 mM, pH 7.3; VivaCell, Cat# C3544-0100) and fetal bovine serum (5.0 vol%; Gibco, Cat# A5256701). For fixed cell imaging, cells were maintained in DPBS (Gibco, Cat# C14190500CP) or HBSS (Gibco, Cat# C14175500CP). Appropriate controls were implemented throughout the experiments to prevent inter-channel crosstalk.

Bright-field imaging was conducted using the following three devices as appropriate: (1) Leica DMi8 inverted

microscope; (2) Olympus IX73 inverted microscope; (3) Sartorius Incucyte S3 instrument placed in a water-jacketed CO_2 incubator with CO_2 (5 vol%) at 37°C.

Microplate assays were conducted using Molecular Devices SpectraMax iD5, i3x, or M2 multimode microplate readers.

Reverse transcription reaction and quantitative real-time polymerase chain reaction (PCR) were performed on an Applied Biosystems 7500 real-time PCR system.

3. Structural modeling

Molecular modeling and Pawley refinement were performed using *BIOVIA Materials Studio* (version 2018). The initial lattice model was conceived from the native connectivity and geometry of the building blocks, and then geometrically optimized using the *Forcite* molecular dynamics module (Universal force fields, Ewald summations). The lattice parameters were further optimized by Pawley refinement using the Reflex package, in conjunction with the experimental PXRD patterns and optimized COF structures, until the residual precision (R_p) and the weighted residual precision (R_{wp}) values converged.

4. Statistical analysis

Data are presented as mean ± SD. No data were excluded from the analyses. The researchers were not blinded to the experimental design or result assessments during most of the experiments since data analyses were based on objectively measurable data; but the histopathology experiments were performed in a blinded fashion.

Statistical analysis and data plotting are performed in *GraphPad Prism* (version 11.0.2), *R* (version 4.5.2), and *OriginPro* (version 10.3.0.197). Normal data distribution was assumed but not formally tested. Variance alignment was verified using the Brown–Forsythe test. The experimental sample size and statistical analysis are described in the corresponding figure captions. The sample sizes were not predetermined using statistical methods but followed accepted conventions.

5. Cell culture

NIH/3T3, 4T1, HC11, and MCF10A cell lines were provided by the Cell Bank, Chinese Academy of Sciences (Shanghai, China).

NIH/3T3 and 4T1 cells were cultured in DMEM (Corning, Cat# 10-013-CVRC) supplemented with HEPES (25 mM, pH 7.3; VivaCell, Cat# C3544-0100), GlutaMAX (2.0 mM; Gibco, Cat# 35050061), fetal bovine serum (10 vol%; Gibco, Cat# A5256701), and Normocin (100 μg/mL; Invivogen, Cat# ant-nr-2) in a water-jacketed incubator with CO_2 (5 vol%) at 37°C.

HC11 cell line was cultured in RPMI-1640 medium (Gibco, Cat# C22400500BT) supplemented with HEPES (25 mM, pH 7.3; VivaCell, Cat# C3544-0100), GlutaMAX (2.0 mM; Gibco, Cat# 35050061), fetal bovine serum (10 vol%; Gibco, Cat# A5256701), and Normocin (100 μg/mL; Invivogen, Cat# ant-nr-2) in a water-jacketed incubator with CO_2 (5 vol%) at 37°C.

MCF10A cells were cultured using MEGM mammary epithelial cell growth medium bullet kit (Lonza, Cat# CC-3150; without GA-1000) supplemented with cholera toxin (100 ng/mL; Sigma–Aldrich, Cat# C8052) in a water-jacketed incubator with CO_2 (5 vol%) at 37°C.

6. Radioresistant cell line

Radiation-resistant 4T1R cells were obtained through the continuous irradiation method. Briefly, 4T1 cells were irradiated twice weekly (every Tuesday and Thursday) with 2 Gy/d of X-rays until a cumulative dose of 80 Gy was reached. The obtained 4T1R cells were cultured in DMEM (Corning, Cat# 10-013-CVRC) supplemented with HEPES (25 mM, pH 7.3; VivaCell, Cat# C3544-0100), GlutaMAX (2.0 mM; Gibco, Cat# 35050061), fetal bovine serum (10 vol%; Gibco, Cat# 26140-079), and Normocin (100 μg/mL; Invivogen, Cat# ant-nr-2) in a water-jacketed incubator with CO_2 (5 vol%) at 37°C.

To validate the successful establishment of radioresistant cell lines, 4T1 and 4T1R cells were exposed to either a single dose of 4 Gy or fractionated doses of 2 Gy/d for 2 d. After an additional 24 h of incubation, their cell viabilities were assessed using alamarBlue HS (Invitrogen, Cat# A50101). The fluorescence signals were recorded at an excitation wavelength of 550 nm and an emission wavelength of 590 nm.

For detecting γH2AX expression of 4T1 and 4T1R cells by flow cytometry, 4T1 and 4T1R cells cultured in 12-well plates were exposed to either a single dose of 4 Gy or fractionated doses of 2 Gy/d for 2 d. After an additional 30 min of incubation, the cells harvested using trypsin–EDTA (Gibco, Cat# 25200056) and subsequently fixed in ethanol (1.0 mL, 75 vol%, precooled at −20°C) for 1 h at −20°C. The cells were washed three times with DPBS and then blocked with DPBS (500 μL) containing normal goat serum (5 vol%; VivaCell, Cat# C2530-0010) and Tween-20 (0.1 vol%; TCI, Cat# T2530) for 2 h at 25°C. The cells were incubated with rabbit anti-γH2AX (100 μL, 1:400 dilution; Cell Signaling Technology, Cat# 9718) for 24 h at 4°C. The cells were washed three times with DPBS (500 μL) containing normal goat serum (5 vol%; VivaCell, Cat# C2530-0010) and Tween-20 (0.1 vol%; TCI, Cat# T2530) and incubated with Alexa Fluor 488-conjugated goat anti-rabbit IgG (H+L) secondary antibody (100 μL, 1:2000 dilution; Abcam, Cat# ab150077) for 1 h at 25°C. The cells were washed once with DPBS prior to flow cytometric analysis. Fluorescence signals of Alexa Fluor 488 were acquired using a 488 nm laser for excitation and detected via the FITC channel.

7. Experimental animals

BALB/c mice (5 weeks of age, female) were purchased from Vital River (Beijing, China) and housed in specific pathogen-free animal research facilities. The mice were provided with ^{60}Co-irradiated food (Beijing Keao Xieli, Cat# 1016706476803973120), aseptic corncob bedding (Beijing Keao Xieli, Cat# 1016711547352604672), and autoclaved water and kept in a room with a 12:12 h light–dark cycle, a temperature of 20–23°C, and 30–70% relative humidity.

When any of the following conditions were met, the mice were considered ethically dead and euthanized immediately: (1) biological death; (2) shock; (3) paralysis; (4) persistent seizures or convulsions; (5) impending death or inability to move; (6) no response to gentle stimulation; (7) difficulty breathing, including salivation or cyanosis; (8) diarrhea or urinary incontinence; (9) inability to eat or drink; (10) weight loss > 4 g; (11) rectal temperature change > 4°C; (12) skin lesions > 30%; (13) local skin ulceration or infection with no relief after povidone iodine disinfection for 24 h; (14) tumor size > 20 mm in any direction; (15) other cases where a veterinarian deems euthanasia necessary.

For euthanasia, cervical dislocation was performed after inhalational anesthesia with isoflurane (RWD Life Science, Cat# R510-22-10) to minimize suffering and ensure death.

8. Monomer synthesis

For TMTA synthesis, a mixture of 2,4,6-tris(4-aminophenyl)-1,3,5-trimethylbenzene (157 mg, 0.4 mmol), acetic acid (20 mL), and acetic anhydride (4 mL) was stirred at 50°C for 6 h. The mixture was poured into ice water (100 mL) and filtered through a 0.45 μm nylon membrane (Millipore, Cat# HNWP04700). The solid was washed with water until free of acidic odor, and dried at 60°C to afford a white product (180 mg, 87% yield based on $C_{33}H_{33}N_3O_3$). ^{1}H NMR (400 MHz, DMSO-d_6): δ/ppm 9.97 (s, 3H), 7.63 (d, J = 8.4 Hz, 6H), 7.09 (d, J = 8.5 Hz, 6H), 2.05 (s, 9H), 1.62 (s, 9H). ^{13}C NMR (101 MHz, DMSO-d_6): δ/ppm 168.71, 139.47, 138.27, 136.65, 132.96, 129.82, 119.57, 24.47, 19.74. HRMS (ESI): m/z calculated for $C_{33}H_{34}N_3O_3$ $[M+H]^+$ 520.2595, found 520.2556; m/z calculated for $C_{66}H_{67}N_6O_6$ $[2M+H]^+$ 1039.5122, found 1039.5111.

For DIBO synthesis, a mixture of 2,5-diiodoterephthalaldehyde (116 mg, 0.3 mmol), neopentyl glycol (83.3 mg, 0.8 mmol), *p*-toluenesulfonic acid monohydrate (115 mg, 0.6 mmol), and toluene (20 mL) was stirred at 110°C for 24 h. After cooling to room temperature, the mixture was mixed with aqueous K_2CO_3 (10 mL, 0.2 g/mL). The product was extracted with dichloromethane and dried over anhydrous Na_2SO_4. The solvent was removed under reduced pressure. The product was recrystallized from dichloromethane as a white powder (158 mg, 94% yield based on $C_{18}H_{24}I_2O_4$). Crystals suitable for single-crystal X-ray diffraction were obtained by slow evaporation of a chloroform solution at 4°C over 8 d. ^{1}H NMR (400 MHz, DMSO-d_6): δ/ppm 7.89 (s, 2H), 5.36 (s, 2H), 3.80–3.54 (m, 8H), 1.19 (s, 6H), 0.76 (s, 6H). ^{13}C NMR (101 MHz, DMSO-d_6): δ/ppm 142.59, 138.10, 103.28, 97.60, 77.19,

30.26, 23.32, 21.67. HRMS (ESI): *m*/*z* calculated for $C_{18}H_{25}I_2O_4$ [M+H]$^+$ 558.9837, found 558.9821; *m*/*z* calculated for $C_{18}H_{24}I_2NaO_4$ [M+Na]$^+$ 580.9656, found 580.9591. CCDC 2533880.

9. Model molecule synthesis

For TMTA hydrolysis, TMTA (5.2 mg, 10 μmol), Pluronic F-127 (1.0 g), and esterase (10 U) were dissolved in an aqueous NaOAc solution (200 mL, 0.1 M, pH 8.8). The mixture was kept at 37°C for 12 h. The product was extracted with dichloromethane, and the organic layer was dried over anhydrous Na_2SO_4. The solvent was removed under reduced pressure, and the product was recrystallized from dichloromethane/methanol (6/1, v/v) to yield a white powder. ^{1}H NMR (400 MHz, DMSO-d_6): δ/ppm 6.79 (d, *J* = 8.1 Hz, 6H), 6.61 (d, *J* = 8.1 Hz, 6H), 5.08 (bs, 6H), 1.65 (s, 9H). ^{13}C NMR (101 MHz, DMSO-d_6): δ/ppm 147.03, 139.96, 133.16, 130.07, 129.98, 114.62, 19.88. HRMS (ESI): *m*/*z* calculated for $C_{27}H_{28}N_3$ [M+H]$^+$ 394.2278, found 394.2284.

For DIBO hydrolysis, DIBO (8.4 mg, 15 μmol) and Pluronic F-127 (1.0 g) were dissolved in an aqueous HOAc/NaOAc solution (200 mL, 0.2 M, pH 4.7). The mixture was kept at 37°C for 4.5 h. The product was extracted with dichloromethane, and the organic layer was dried over anhydrous Na_2SO_4. The solvent was removed under reduced pressure, and the product was recrystallized from dichloromethane to yield a yellow powder. ^{1}H NMR (400 MHz, DMSO-d_6): δ/ppm 9.91 (s, 2H), 8.22 (s, 2H). ^{13}C NMR (101 MHz, DMSO-d_6): δ/ppm 194.23, 142.18, 139.52, 99.68. HRMS (ESI): *m*/*z* calculated for $C_8H_4I_2O_2$ [M]$^+$ 385.8301, found 385.8310.

To synthesize 1,1'-(2,5-diiodo-1,4-phenylene)bis(*N*-phenylmethanimine), acetanilide (8.1 mg, 60 μmol), DIBO (8.4 mg, 15 μmol), esterase (10 U), and Pluronic F-127 (1.0 g) were dissolved in an aqueous NaOAc solution (200 mL, 0.1 M, pH 8.8). The mixture was kept at 37°C for 4.5 h. Acetic acid (1.2 mL) was added to the mixture, followed by incubation at 37°C for 24 h. The mixture was mixed with DMF (100 mL, precool at 4°C) and filtered through a 0.45 μm nylon membrane (Millipore, Cat# HNWP04700). The obtained solid was washed with dichloromethane and dried at room temperature to obtain the target product. Crystals suitable for single-crystal X-ray diffraction were obtained by slow evaporation of a THF solution over approximately 70 d at 4°C. ^{1}H NMR (400 MHz, THF-d_8): δ/ppm 8.74 (s, 2H), 8.69 (s, 2H), 7.49–7.41 (m, 4H), 7.37–7.27 (m, 6H). ^{13}C NMR (101 MHz, THF-d_8): δ/ppm 160.66, 151.04, 140.03, 139.42, 129.09, 126.77, 121.07, 100.08. HRMS (ESI): *m*/*z* calculated for $C_{20}H_{15}I_2N_2$ [M+H]$^+$ 536.9319, found 536.9322. CCDC 2533881.

10. COF synthesis in water

To synthesize **UM-113**, TMTA (26.0 mg, 50 μmol), DIBO (41.9 mg, 75 μmol), esterase (50 U), and Pluronic F-127 (5.0 g) were dissolved in an aqueous NaOAc solution (1.0 L, 0.1 M, pH 8.8). The mixture was kept at 37°C for 12 h. Acetic acid (6.5 mL) was added to the mixture to adjust the pH to 4.7, followed by incubation at 37°C for 24 h. Triethylamine (10 mL) was subsequently introduced to the reaction mixture to quench the reaction. After 15 min, the precipitate was separated by filtration using a 0.2 μm nylon membrane (Millipore, Cat# GNWP04700) and

rinsed thrice with water and once with ethanol. The powder was dried in supercritical carbon dioxide and degassed at 40°C for 2 h to obtain the yellow solid product (37.0 mg, 81% yield based on $C_{78}H_{54}I_6N_6$).

To track the synthesis kinetics of **UM-113**, the reaction system was miniaturized to a 4.0 mL scale in a 6-well plate. The absorbance at 425 nm was measured for 51 h using a microplate reader, with the plate chamber maintained at 37°C.

To synthesize **UM-114(AA')**, 2,5-diiodoterephthalaldehyde (324 mg, 0.84 mmol) and 2,4,6-tris(4-aminophenyl)-1,3,5-trimethylbenzene (220 mg, 0.56 mmol) were added to a round-bottom flask containing methyl acetate (400 mL). The mixture was stirred at 600 rpm for 10 min at 25°C, after which acetic acid (20 mL) was introduced, and the resulting suspension was further stirred at 600 rpm for 72 h at 37°C. Triethylamine (20 mL) was subsequently added to the reaction system to quench the reaction. Following 15 min of incubation, the precipitate was isolated by filtration through a 0.2 μm nylon membrane (Millipore, Cat# GNWP04700), and rinsed three times with water and once with ethanol. The collected powder was dried in supercritical carbon dioxide and degassed at 40°C for 2 h, obtaining a yellow solid product (330 mg, 64% yield based on $C_{78}H_{54}I_6N_6$).

To synthesize amorphous polymers using acetic acid, 2,5-diiodoterephthalaldehyde (32.4 mg, 84 μmol) and 2,4,6-tris(4-aminophenyl)-1,3,5-trimethylbenzene (22.0 mg, 56 μmol) were added to a round-bottom flask containing either acetonitrile or phenylacetonitrile (40 mL). The mixture was stirred at 600 rpm for 10 min at 25°C, after which acetic acid (2.0 mL) was introduced. The resulting suspension was further stirred at 600 rpm for 3–7 d at a temperature range of 25–120°C. Triethylamine (2.0 mL) was subsequently added to the reaction system to quench the reaction. The precipitate was collected by centrifugation at 4000 rpm for 10 min at 4°C, then washed three times with the corresponding solvent (acetonitrile or phenylacetonitrile) and once with ethanol. Finally, the collected powder was dried in supercritical carbon dioxide and degassed at 40°C for 2 h, obtaining the yellow solid product. The amorphous polymer obtained by reacting at 37°C for 3 d is called **UM-114(POP)**.

To synthesize amorphous polymers using triethylamine, 2,5-diiodoterephthalaldehyde (32.4 mg, 84 μmol) and 2,4,6-tris(4-aminophenyl)-1,3,5-trimethylbenzene (22.0 mg, 56 μmol) were added to a round-bottom flask containing dichloromethane (36–40 mL). The mixture was stirred at 600 rpm for 10 min at 25°C, after which triethylamine (0–4.0 mL) was introduced. The resulting solution was further stirred at 600 rpm for 7 d at 40°C. After the solution was cooled to 25°C, 3 mL of the solution was transferred to a glass bottle to assess precipitate formation.

11. COF synthesis within cells

To assess Pluronic F-127–induced cell membrane permeabilization via flow cytometry, 4T1R cells grown in 12-well plates were incubated with DMEM (1.0 mL) containing PI (5.0 μM; MedChemExpress, Cat# HY-D0815) and Pluronic F-127 (0 or 0.04 wt%) for 2 h in a CO_2 incubator. The cells were harvested with trypsin–EDTA (Gibco,

Cat# 25200056) and analyzed on a flow cytometer using a 488 nm laser for excitation and the PE channel for detection. Dead cells, identified by high PI fluorescence intensity, were excluded from the analysis.

To assess Pluronic F-127–induced cell membrane permeabilization by confocal laser scanning fluorescence microscopy, 4T1R cells cultured in 4-chamber glass-bottom dishes were treated with DMEM (400 μL) containing fluorexon (1.0 μM; MedChemExpress, Cat# HY-D0040) and Pluronic F-127 (0 or 0.04 wt%) for 2–6 h in a CO_2 incubator. The cells were washed twice with DPBS and incubated with Hoechst 33342 (400 μL, 20 μM; MedChemExpress, Cat# HY-15559A) for 10 min in a CO_2 incubator. Following two additional washes with DPBS, confocal laser scanning fluorescence images were captured. The blue images of Hoechst 33342 were recorded at an excitation wavelength of 405 nm and an emission wavelength of 420–450 nm. The green images of fluorexon were recorded at an excitation wavelength of 488 nm and an emission wavelength of 500–540 nm.

To investigate the energy-dependent pinocytosis of Pluronic F-127, 4T1R cells cultured in 12-well plates were pretreated with 2-deoxy-*D*-glucose (2.0 mL, 20 mM; MedChemExpress, Cat# HY-13966), dynasore (2.0 mL, 75 μM; Selleck, Cat# S8047), nystatin (2.0 mL, 20 μM; Selleck, S1934), genistein (2.0 mL, 100 μM; MedChemExpress, Cat# HY-14596), methyl-β-cyclodextrin (2.0 mL, 5.0 mg/mL; MedChemExpress, Cat# HY-101461), chlorpromazine (2.0 mL, 50 μM; Sigma–Aldrich, Cat# C8138), or amiloride (2.0 mL, 0.2 mM; MedChemExpress, Cat# HY-B0285B) for 1 h. Subsequently, Cy3-labeled Pluronic F-127 (50 μL, 1.6 wt%) was introduced into the wells and the cells were incubated for an additional 2 h. The cells were harvested with trypsin–EDTA (Gibco, Cat# 25200056) and analyzed on a flow cytometer using a 488 nm laser for excitation and the PE channel for detection.

For monitoring intracellular COF synthesis via flow cytometry, 4T1R cells grown in 12-well plates were incubated with DMEM (1.0 mL) containing TMTA (4.0 μM), DIBO (6.0 μM), and Pluronic F-127 (0.04 wt%) for 0–36 h. The cells were harvested with trypsin–EDTA (Gibco, Cat# 25200056) and analyzed on a flow cytometer using a 405 nm laser for excitation and the AmCyan channel for detection. Methanol-fixed cells were used as the control. The same protocol was applied to HC11, NIH/3T3, and MCF10A cells, with DMEM substituted by their respective culture media.

For isolating intracellularly synthesized COFs from 4T1R cells via a triethylamine precipitation method for PXRD analysis, 4T1R cells cultured in T225 flasks were treated with DMEM (40 mL) containing TMTA (4.0 μM), DIBO (6.0 μM), and Pluronic F-127 (0.04 wt%) for 24 h. Then, the cells were washed once with DPBS, harvested using trypsin–EDTA (Gibco, Cat# 25200056), and centrifuged at 1000 rpm for 10 min at 4°C. The cells were washed once with DPBS and then lysed in a lysis buffer (1.0 vol% Triton X-100, 25 mM HEPES, 150 mM NaCl, pH 7.3) under the assistance of ultrasound at 4°C. The lysates were centrifuged at 12000 rpm for 15 min at 4°C, and the supernatants were isolated to exclude cell debris and gelatinous substances. The supernatants were mixed with an equal volume of dichloromethane, sonicated for 10 min, and centrifuged at 12000 rpm for 15 min at 4°C to remove precipitated proteins. Subsequently, 1/5 volume of triethylamine was added to the resulting supernatants followed

by incubation at 40°C for 2 h. The resulting precipitates were washed once with water and twice with ethanol. To prepare the PXRD samples, the precipitates were dispersed in ethanol, deposited onto silicon wafers (approximately 3 × 3 mm), and air-dried.

For isolating intracellularly synthesized COFs from 4T1R cells via a density gradient centrifugation method for PXRD analysis, 4T1R cells were cultured in T225 flasks and incubated for 24 h with DMEM (40 mL per flask) supplemented with TMTA (4.0 μM), DIBO (6.0 μM), and Pluronic F-127 (0.04 wt%). The cells from 10 flasks were washed once with DPBS and harvested using a cell scraper. Lysosome isolation was performed using a commercial lysosome enrichment kit (Thermo Scientific, Cat# 89839) via an iodixanol gradient (15%, 17%, 20%, 23%, 27%, and 30%). After ultracentrifugation, the uppermost band was collected, diluted with three volumes of washing buffer (150 mM NaCl, 100 mM Na_3PO_4, pH 7.2), and centrifuged at 18000 ×*g* and 4°C for 30 min. The obtained pellet was resuspended in CHAPS lysis buffer (2 wt% CHAPS, 25 mM Tris, 150 mM NaCl, pH 7.2) and incubated for 10 min. After a subsequent centrifugation at 18000 ×*g* and 4°C for 15 min, the resulting pellet was washed three times with methanol, deposited onto silicon wafers (approximately 5 × 5 mm), and air-dried for PXRD characterization.

For confocal laser scanning fluorescence imaging of intracellularly synthesized COF nanoparticles, 4T1R cells cultured in 4-chamber glass-bottom dishes were treated with DMEM (400 μL) containing TMTA (4.0 μM), DIBO (6.0 μM), and Pluronic F-127 (0.04 wt%) for 24 h. The cells were washed twice with DPBS, incubated with LysoTracker Red DND-99 (200 μL, 50 nM; Invitrogen, Cat# A66466) for 15 min in a CO_2 incubator. The cells were washed twice with DPBS, incubated with Nuclear Red LCS1 (200 μL, 1.0 μM; AAT Bioquest, Cat# 17542) for 10 min in a CO_2 incubator. The cells were washed twice with DPBS, and confocal laser scanning fluorescence images were captured. The green images of COFs were recorded at an excitation wavelength of 405 nm and an emission wavelength of 500–540 nm. The red images of LysoTracker Red DND-99 were recorded at an excitation wavelength of 561 nm and an emission wavelength of 580–620 nm. The cyan images of Nuclear Red LCS1 were recorded at an excitation wavelength of 633 nm and an emission wavelength of 650–700 nm. The obtained raw images were smoothed using a Gaussian blur with a radius of 1 pixel to reduce noise, and subsequently converted to an 8-bit format for colocalization analysis using *ImageJ* (version 1.54p).

For bio-TEM imaging of intracellularly synthesized COFs, 4T1R cells cultured in 10 cm dishes were treated with DMEM (10 mL) containing TMTA (4.0 μM), DIBO (6.0 μM), and Pluronic F-127 (0.04 wt%) for 24 h. The cells were carefully washed once with DPBS, fixed in glutaraldehyde (10 mL, 2.5 vol%) for 5 min, and collected using cell scrapers. The cells were collected via centrifugation at 1200 rpm for 10 min at 4°C, resuspended in glutaraldehyde (1.0 mL, 2.5 vol%), and stored at 4°C. On the day of analysis, the cells were collected by centrifugation at 1200 rpm for 10 min at 4°C, dehydrated through a graded ethanol series, and embedded in epoxy resin. All samples were cut into 50–80 nm thick sections for bio-TEM examination.

For evaluating cell proliferation in the presence of Pluronic F-127 and organic monomers, 4T1R cells cultured in 96-well microplates were treated with DMEM (100 μM) containing TMTA (0–128 μM), DIBO (0–128 μM), or Pluronic F-127 (0–2.0 wt%) for 24 h. The cells were washed once with DPBS, and their cell viabilities were analyzed by a cell counting kit-8 (Dojindo, Cat# CK04). Untreated cells were used as the control group while methanol-treated cells were used as the blank. The cell viability data were fitted to a logistic equation and iterated to convergence using the orthogonal distance regression algorithm.

For assessing cytotoxicity of Pluronic F-127 and organic monomers by flow cytometry, 4T1R cells cultured in 12-well plates were treated with DMEM (1.0 mL) containing TMTA (4.0 μM), DIBO (6.0 μM), and Pluronic F-127 (0.04 wt%) for 24 h. The cells were washed once with DPBS and harvested using trypsin–EDTA (Gibco, Cat# 25200056), and mixed with 7-aminoactinomycin D (400 μL, 5.0 μM; MedChemExpress, Cat# HY-D1020). After 10 min, flow cytometric analysis was performed using a 488 nm laser for excitation and the PC5.5 channel for detection. Cells treated with ethanol (1.0 mL, 75 vol%, precooled at 4°C) for 12 h at 4°C served as the positive control.

To monitor intracellular COF synthesis in HC11 and 4T1R co-cultures by flow cytometry, a co-culture model with fluorescent labels was established. HC11 and 4T1R cells were stained with sulfo-Cy7.5 NHS ester (1.0 mL, 2.0 μM; MedChemExpress, Cat# HY-D1568) (1.0 mL, 2.0 μM, Invitrogen, Cat# C34552) and sulfo-Cy3 NHS ester (1.0 mL, 2.0 μM; MedChemExpress, Cat# HY-D0823), respectively, for 1.5 h in a CO_2 incubator. The cells were washed three times with DMEM and co-seeded into T25 flasks at a 1:1 ratio. After 4 h, the co-cultures were treated with DMEM (5.0 mL) containing TMTA (4.0 μM), DIBO (6.0 μM), and Pluronic F-127 (0.04 wt%) for 24 h. The co-cultures were then harvested with trypsin–EDTA (Gibco, Cat# 25200056) and analyzed by flow cytometry. The signals of sulfo-Cy7.5 NHS ester were detected at the APC-A750 channel with a 640 nm laser for excitation. The signals of sulfo-Cy3 NHS ester were detected at the PE channel with a 488 nm laser for excitation. The signals of COFs were detected at the KO525 channel with a 405 nm laser for excitation.

To visualize intracellular COF synthesis in HC11 and 4T1R co-cultures via confocal laser scanning fluorescence microscopy using a μ-slide 2-well co-culture chamber slide (ibidi, Cat# 81806), HC11 and 4T1R cells were seeded into the inner reservoirs of the slide. After 24 h, the culture medium was replaced with DMEM (600 μL) containing TMTA (4.0 μM), DIBO (6.0 μM), and Pluronic F-127 (0.04 wt%) added to the main wells. Following a 24 h incubation, the co-cultures were washed twice with DPBS and fixed with paraformaldehyde (600 μL, 4 wt%) for 15 min at 25°C. The fixed cells were washed twice with DPBS and stained with PI (600 μL, 5.0 μM; MedChemExpress, Cat# HY-D0815) for 30 min at 25°C. After two additional washes with DPBS, confocal laser scanning fluorescence images were captured. The green images of COFs were recorded at an excitation wavelength of 405 nm and an emission wavelength of 500–540 nm. The red images of PI were recorded at an excitation wavelength of 561 nm and an emission wavelength of 590–630 nm.

12. X-ray-induced ROS generation

For detection of X-ray-induced ·OH generation, Pluronic F-127 (1.0 mL, 1.0 wt%), TMTA (1.0 mL, 100 μM), DIBO (1.0 mL, 150 μM), and **UM-113** (1.0 mL; 300 μM, iodine equiv.) were added in a 12-well plate. HPF (2.0 μL, 2.5 mM in DMF; Selleck, Cat# E7752) was added in to each well and exposed to X-ray irradiation (0–10 Gy). After 30 min, the fluorescence spectra were recorded at an excitation wavelength of 455 nm.

For detection of X-ray-induced $\cdot O_2^-$ generation, Pluronic F-127 (1.0 mL, 1.0 wt%), TMTA (1.0 mL, 100 μM), DIBO (1.0 mL, 150 μM), and **UM-113** (1.0 mL; 300 μM, iodine equiv.) were added in a 12-well plate. DHE (2.0 μL, 5.0 mM in DMSO; Selleck, Cat# S6792) was added in to each well and exposed to X-ray irradiation (0–10 Gy). After 30 min, the fluorescence spectra were recorded at an excitation wavelength of 500 nm.

For detection of X-ray-induced H_2O_2 generation, Pluronic F-127 (1.0 mL, 1.0 wt%), TMTA (1.0 mL, 100 μM), DIBO (1.0 mL, 150 μM), and **UM-113** (1.0 mL; 300 μM, iodine equiv.) were added in a 12-well plate. FBBBE (5.0 μL, 10 mM in DMSO; MedChemExpress, Cat# HY-D1693) was added in to each well and exposed to X-ray irradiation (0–10 Gy). After 30 min, the fluorescence spectra were recorded at an excitation wavelength of 455 nm.

For detection of X-ray-induced $^1O_2$ generation, Pluronic F-127 (1.0 mL, 1.0 wt%), TMTA (1.0 mL, 100 μM), DIBO (1.0 mL, 150 μM), and **UM-113** (1.0 mL; 300 μM, iodine equiv.) were added in a 12-well plate. SOSG (2.0 μL, 2.0 mM in methanol; Invitrogen, Cat# S36002) was added in to each well and exposed to X-ray irradiation (0–10 Gy). After 30 min, the fluorescence spectra were recorded at an excitation wavelength of 455 nm.

For detection of X-ray-induced total ROS generation in vitro by flow cytometry, 4T1R cells cultured in 12-well plates were incubated with DMEM (1.0 mL) containing TMTA (4.0 μM), DIBO (6.0 μM), and Pluronic F-127 (0.04 wt%) for 24 h. The cells were washed twice with DPBS and exposed to X-ray irradiation (0–4 Gy). After 30 min, the cells were incubated with CellROX Deep Red (500 μL, 5.0 μM; Invitrogen, Cat# C10422) for 30 min in a CO_2 incubator. The cells were washed twice with DPBS and harvested with trypsin–EDTA (Gibco, Cat# 25200056). The cells were washed once with DPBS for flow cytometric analysis. The signals of CellROX Deep Red were detected at the APC channel with a 640 nm laser for excitation.

13. Radiotherapy in vitro

For γH2AX immunofluorescence staining of 4T1R cells, 4T1R cells cultured in 8-chamber slides were treated with DMEM (400 μL) containing TMTA (4.0 μM), DIBO (6.0 μM), and Pluronic F-127 (0.04 wt%) for 24 h. The cells were washed twice with DPBS and exposed to X-ray irradiation (0–4 Gy). After an additional 30 min of incubation, the cells were fixed in paraformaldehyde (300 μL, 4 wt%) for 15 min at 25°C and washed three times with DPBS. The cells were permeabilized with Triton X-100 (300 μL, 0.5 vol%; TCI, Cat# P1775) for 5 min and washed twice with DPBS. The cells were then blocked with DPBS (300 μL) containing normal goat serum (5 vol%; VivaCell,

Cat# C2530-0010) and Tween-20 (0.1 vol%; TCI, Cat# T2530) for 2 h at 25°C. The cells were incubated with rabbit anti-γH2AX (100 μL, 1:400 dilution; Cell Signaling Technology, Cat# 9718) for 24 h at 4°C. The cells were washed three times with DPBS (300 μL) containing normal goat serum (5 vol%; VivaCell, Cat# C2530-0010) and Tween-20 (0.1 vol%; TCI, Cat# T2530) and incubated with Alexa Fluor Plus 647-conjugated goat anti-rabbit IgG (H+L) highly cross-adsorbed secondary antibody (200 μL, 1:1000 dilution; Invitrogen, Cat# A32733) for 1 h at 25°C. The cells were washed three times with DPBS and incubated with PI (300 μL, 5.0 μM; MedChemExpress, Cat# HY-D0815) for 30 min at 25°C. The cells were washed twice with DPBS and confocal laser scanning fluorescence images were captured. The green images of Alexa Fluor Plus 647 were recorded at an excitation wavelength of 633 nm and an emission wavelength of 650–700 nm. The red images of PI were recorded at an excitation wavelength of 561 nm and an emission wavelength of 590–630 nm.

For evaluating cell proliferation, 4T1R cells cultured in 96-well black microplates were treated with DMEM (100 μM) containing TMTA (4.0 μM), DIBO (6.0 μM), and Pluronic F-127 (0.04 wt%) for 24 h. The cells were washed twice with DPBS and exposed to X-ray irradiation (0–6 Gy). After an additional 24–72 h of incubation, their cell viabilities were analyzed using alamarBlue HS (Invitrogen, Cat# A50101). The fluorescence signals were recorded at an excitation wavelength of 550 nm and an emission wavelength of 590 nm. The cell viability (S) was fitted with the linear quadratic model using the formula $S = \exp(-\alpha D - \beta D^2)$, where D is the X-ray dose, α represents linear irreparable damage, and β represents quadratic reparable damage.

For clonogenic assays, 4T1R cells were cultured at a density of 10^3 cells/well in 6-well plates for 24 h, treated with DMEM (2.0 mL) containing TMTA (4.0 μM), DIBO (6.0 μM), and Pluronic F-127 (0.04 wt%) for 24 h. The cells were washed twice with DPBS and exposed to X-ray irradiation (0–4 Gy). After 6 d of incubation, the cells were fixed with methanol (1.0 mL, precooled at −20°C) for 10 min and washed twice with DPBS. The cells were stained with fresh Giemsa staining solution (1.0 mL, 1:10 dilution; Solarbio, Cat# G1015) for 12 h at 25°C. The plates were washed with water, air-dried, and photographed.

14. Ferroptosis in vitro

For detecting lysosomal alkalization via flow cytometry, 4T1R cells cultured in 12-well plates were treated with DMEM (1.0 mL) containing TMTA (4.0 μM), DIBO (6.0 μM), and Pluronic F-127 (0.04 wt%) for 24 h. Following two washes with DPBS, the cells were exposed to X-ray irradiation (0–4 Gy). After an additional 24 h, the cells were incubated with LysoTracker Deep Red (1.0 mL, 50 nM; Invitrogen, Cat# L12492) for 30 min in a CO_2 incubator. Following two washes with DPBS, the cells harvested using trypsin–EDTA (Gibco, Cat# 25200056) and washed once with DPBS prior to flow cytometric analysis. The signals of LysoTracker Deep Red were detected at the APC channel with a 638 nm laser for excitation.

For detecting Fe(II) by confocal laser scanning fluorescence microscopy, 4T1R cells cultured in 4-chamber glass-

bottom dishes were treated with DMEM (400 μL) containing TMTA (4.0 μM), DIBO (6.0 μM), and Pluronic F-127 (0.04 wt%) for 24 h. The cells were washed twice with DPBS and exposed to X-ray irradiation (0–4 Gy). After 24 h, the cells were washed twice with DPBS and incubated with Nuclear Red LCS1 (400 μL, 1.0 μM; AAT Bioquest, Cat# 17542) for 10 min in a CO_2 incubator. Following two additional washes with DPBS, the cells were incubated with FerroOrange (400 μL, 1.0 μM; Dojindo, Cat# F374) for 30 min in a CO_2 incubator. Confocal imaging was performed immediately without further washing. The green images of FerroOrange were recorded at an excitation wavelength of 543 nm and an emission wavelength of 560–600 nm. The red images of Nuclear Red LCS1 were recorded at an excitation wavelength of 633 nm and an emission wavelength of 650–700 nm.

For detecting lipid peroxidation by flow cytometry, 4T1R cells cultured in 12-well plates were incubated with DMEM (1.0 mL) containing TMTA (4.0 μM), DIBO (6.0 μM), and Pluronic F-127 (0.04 wt%) for 24 h. The cells were washed twice with DPBS and exposed to X-ray irradiation (0–4 Gy). After 24 h, the cells were incubated with BODIPY 665/676 (500 μL, 1.0 μM; Invitrogen, Cat# B3932) for 30 min in a CO_2 incubator. Following two washes with DPBS, the cells were harvested using trypsin–EDTA (Gibco, Cat# 25200056) and subsequently washed once with DPBS prior to flow cytometric analysis. Fluorescence signals were acquired using a 561 nm laser for excitation and detected via the PE channel.

For detecting oxidative stress by flow cytometry, 4T1R cells cultured in 12-well plates were incubated with DMEM (1.0 mL) containing TMTA (4.0 μM), DIBO (6.0 μM), and Pluronic F-127 (0.04 wt%) for 24 h. The cells were washed twice with DPBS and exposed to X-ray irradiation (0–4 Gy). After 24 h, the cells were incubated with ROS Brite 570 (500 μL, 2.0 μM; AAT Bioquest, Cat# 16000) for 30 min in a CO_2 incubator. Following two washes with DPBS, the cells were harvested using trypsin–EDTA (Gibco, Cat# 25200056) and washed once with DPBS prior to flow cytometric analysis. Fluorescence signals were acquired using a 488 nm laser for excitation and detected via the PE channel.

For detecting intracellular GSH and GSSG, 4T1R cells cultured in 6-well plates were treated with DMEM (2.0 mL) containing TMTA (4.0 μM), DIBO (6.0 μM), and Pluronic F-127 (0.04 wt%) for 24 h. The cells were washed twice with DPBS and exposed to X-ray irradiation (0–4 Gy). After 24 h, the cells were carefully washed with DPBS and collected using cell scrapers. The GSH and GSSG levels were measured using a GSH/GSSG assay kit (MedChemExpress, Cat# HY-K0311) following the manufacturer's guideline. The GSH and GSSG levels were normalized to cell numbers.

For detecting intracellular MDA, 4T1R cells cultured in 10 cm dishes were treated with DMEM (10 mL) containing TMTA (4.0 μM), DIBO (6.0 μM), and Pluronic F-127 (0.04 wt%) for 24 h. The cells were washed twice with DPBS and exposed to X-ray irradiation (0–4 Gy). After 24 h, the cells were carefully washed with DPBS and collected using cell scrapers. The MDA levels were measured using an MDA assay kit (MedChemExpress, Cat# HY-K0319) following the manufacturer's guideline. The MDA contents were normalized to the total protein amount quantified

using a Bradford assay kit (Epizyme, Cat# ZJ104).

For detecting *Ptgs2* mRNA, 4T1R cells cultured in 6-well plates were treated with DMEM (2.0 mL) containing TMTA (4.0 μM), DIBO (6.0 μM), and Pluronic F-127 (0.04 wt%) for 24 h. The cells were washed twice with DPBS and exposed to X-ray irradiation (0–4 Gy). After an additional 24 h of incubation, the 4T1R cells were rinsed twice with DPBS for RNA extraction using RNAfast200 kit (Fastagen, Cat# 220010). The extracted RNA was dissolved in water and the concentration was determined by measuring absorbance at 260 nm. First-strand complementary DNA was synthesised from 0.5–1.0 μg of total RNA using a HiFiScript cDNA synthesis kit (CoWin Biosciences, Cat# CW2569) following the manufacturer's protocol. Real-time PCR was performed by FastSYBR mixture (CoWin Biosciences, Cat# CW0955) according to the manufacturer's protocol. The threshold cycle (*Ct*) values for each gene were normalized to those of *Actb*, and the $2^{-\Delta\Delta Ct}$ method was used for quantitative analysis. Results are represented as fold changes from the control group. The following primers were used here: *Actb*, forward 5'-CATTGCTGACAGGATGCAGAAGG-3' and reverse 5'-TGCTGGAAGGTGGACAGTGAGG-3'; *Ptgs2*, forward 5'-GCGACATACTCAAGCAGGAGCA-3' and reverse 5'-AGTGGTAACCGCTCAGGTGTTG-3'.

For cell death rescue experiments, 4T1R cells cultured in 96-well black microplates were treated with DMEM (100 μM) containing TMTA (4.0 μM), DIBO (6.0 μM), and Pluronic F-127 (0.04 wt%) for 24 h. The cells were washed twice with DPBS and exposed to X-ray irradiation (0–4 Gy). The cells were incubated with DMEM (100 μM) in the absence or presence of DMSO (0.1 vol%; Sigma–Aldrich, Cat# D2650), NAC (5.0 mM; MedChemExpress, Cat# HY-B0215), GSHee (2.0 mM; Sigma–Aldrich, Cat# G1404), Fer-1 (1.0 μM; Selleck, Cat#S7243), Lip-1 (0.5 μM; MedChemExpress, Cat# HY-12726), DFOM (100 μM; MedChemExpress, Cat# HY-B0988), Z-VAD-FMK (25 μM; Selleck, Cat# S7023), Nec-1s (5.0 μM; MedChemExpress, Cat# HY-14622A), and VX765 (20 μM; MedChemExpress, Cat# HY-13205). The cells were washed twice with DPBS and their cell viabilities were analyzed using alamarBlue HS (Invitrogen, Cat# A50101). The fluorescence signals were recorded at an excitation wavelength of 550 nm and an emission wavelength of 590 nm.

15. Apoptosis in vitro

For detecting mitochondrial membrane potentials by flow cytometry, 4T1R cells cultured in 12-well plates were incubated with DMEM (1.0 mL) containing TMTA (4.0 μM), DIBO (6.0 μM), and Pluronic F-127 (0.04 wt%) for 24 h. The cells were washed twice with DPBS and exposed to X-ray irradiation (0–4 Gy). After 24 h, the cells were washed twice with DPBS and harvested with trypsin–EDTA (Gibco, Cat# 25200056). The cells were incubated with JC10 (200 μL, 2.0 μM; Yeasen, Cat# 40707ES03) for 30 min in a CO_2 incubator. The cells were mixed with DMEM (1.0 mL) for flow cytometric analysis. The green signals were detected at the FITC channel with a 488 nm laser for excitation. The red signals were detected at the PE channel with a 561 nm laser for excitation.

For detecting phosphatidylserine translocation by flow cytometry, 4T1R cells cultured in 12-well plates were

incubated with DMEM (1.0 mL) containing TMTA (4.0 μM), DIBO (6.0 μM), and Pluronic F-127 (0.04 wt%) for 24 h. Following two washes with DPBS, the cells were subjected to X-ray irradiation (0–4 Gy). After an additional 24 h incubation, the cells were washed twice with DPBS and harvested using trypsin–EDTA (Gibco, Cat# 25200056). The cells were washed twice with DMEM and incubated for 30 min in a CO_2 incubator. The cells were stained with an annexin V–Alexa Fluor 647/PI apoptosis detection kit (Yeasen, Cat# 40304ES60) following the manufacturer's guidelines. Flow cytometric analysis was performed. The signals of Alexa Fluor 647 were detected at the APC channel with a 640 nm laser for excitation. The signals of PI were detected at the PE-Texas Red channel with a 561 nm laser for excitation.

For detecting caspase 3 activity, 4T1R cells cultured in 6-well plates were incubated with DMEM (2.0 mL) containing TMTA (4.0 μM), DIBO (6.0 μM), and Pluronic F-127 (0.04 wt%) for 24 h. The cells were washed twice with DPBS and exposed to X-ray irradiation (0–4 Gy). After 24 h, the cells were washed twice with DPBS and used for detecting caspase 3 activity using a caspase 3 activity assay kit (MedChemExpress, Cat# HY-K2611) according to the manufacturer's guidelines. The absorbance at 405 nm was measured every 30 s for a total of 2 h, and the increase rate of the absorbance reflects caspase 3 activity. Cells treated with staurosporine (2.0 μM; TargetMol, Cat# T3211) for 10 h were used as the positive control of apoptosis.

16. Mitotic catastrophe in vitro

For imaging cytoskeleton by confocal laser scanning fluorescence microscopy, 4T1R cells cultured in 4-chamber glass-bottom dishes were treated with DMEM (400 μL) containing TMTA (4.0 μM), DIBO (6.0 μM), and Pluronic F-127 (0.04 wt%) for 24 h. The cells were washed twice with DPBS and exposed to X-ray irradiation (0–4 Gy). After 72 h, the cells were washed twice with DPBS, fixed in paraformaldehyde (400 μL, 4 wt%) for 15 min at 4°C, and washed three times with DPBS. The cells were permeabilized with Triton X-100 (400 μL, 0.1 vol%; TCI, Cat# P1775) for 5 min and washed twice with DPBS. The cells were incubated with DPBS (400 μL) containing TRITC-labeled phalloidin (1:500 dilution; MedChemExpress, Cat# HY-K0903) and Nuclear Red LCS1 (1.0 μM; AAT Bioquest, Cat# 17542) for 30 min at room temperature. The cells were washed twice with DPBS. Confocal laser scanning fluorescence images were captured. The green images of TRITC were recorded at an excitation wavelength of 561 nm and an emission wavelength of 580–620 nm. The red images of Nuclear Red LCS1 were recorded at an excitation wavelength of 633 nm and an emission wavelength of 650–700 nm.

For detecting cell cycles by flow cytometry, 4T1R cells cultured in 6-well plates were incubated with DMEM (2.0 mL) containing TMTA (4.0 μM), DIBO (6.0 μM), and Pluronic F-127 (0.04 wt%) for 24 h. The cells were washed twice with DPBS and exposed to X-ray irradiation (0–4 Gy). After 72 h, the cells were washed twice with DPBS, harvested with trypsin–EDTA (Gibco, Cat# 25200056), and fixed in ethanol (1.0 mL, 75 vol%, precooled at −20°C) for 24 h at −20°C. The cells were washed twice with DPBS. The cells were suspended in FxCycle PI/RNase solution (500 μL; Invitrogen, Cat# F10797) at a concentration of approximately 10^6 cells/mL. After incubation for 30 min,

the signals of PI were detected at the PE-Texas Red channel with a 561 nm laser for excitation.

For COX2 immunofluorescence staining, 4T1R cells cultured in 8-chamber slides were treated with DMEM (400 μL) containing TMTA (4.0 μM), DIBO (6.0 μM), and Pluronic F-127 (0.04 wt%) for 24 h. The cells were washed twice with DPBS and exposed to X-ray irradiation (0–4 Gy). After incubation for 72 h, the cells were fixed in paraformaldehyde (300 μL, 4 wt%) for 15 min at 25°C and washed three times with DPBS. The cells were permeabilized with Triton X-100 (300 μL, 0.5 vol%; TCI, Cat# P1775) for 5 min and washed twice with DPBS. The cells were then blocked with DPBS (300 μL) containing normal goat serum (5 vol%; VivaCell, Cat# C2530-0010) and Tween-20 (0.1 vol%; TCI, Cat# T2530) for 2 h at 25°C. The cells were incubated with rabbit anti-COX2 (100 μL, 1:400 dilution; Cell Signaling Technology, Cat# 12282S) for 24 h at 4°C. The cells were washed three times with DPBS (300 μL) containing normal goat serum (5 vol%; VivaCell, Cat# C2530-0010) and Tween-20 (0.1 vol%; TCI, Cat# T2530) and incubated with Alexa Fluor 594-conjugated goat anti-rabbit IgG (H+L) cross-adsorbed secondary antibody (100 μL, 1:500 dilution; Invitrogen, Cat# A11012) for 2 h at 25°C. The cells were washed three times with DPBS and incubated with Nuclear Red LCS1 (300 μL,1.0 μM; AAT Bioquest, Cat# 17542) for 30 min at 25°C. The cells were washed twice with DPBS and confocal laser scanning fluorescence images were captured. The green images of Nuclear Red LCS1 were recorded at an excitation wavelength of 633 nm and an emission wavelength of 650–700 nm. The red images of Alexa Fluor 594 were recorded at an excitation wavelength of 561 nm and an emission wavelength of 580–620 nm.

For cell senescence-related β-galactosidase staining, 4T1R cells cultured in 12-well plates were incubated with DMEM (1.0 mL) containing TMTA (4.0 μM), DIBO (6.0 μM), and Pluronic F-127 (0.04 wt%) for 24 h. The cells were washed twice with DPBS and exposed to X-ray irradiation (0–4 Gy). After 72 h, cells were washed twice with DPBS and stained using a cell senescence β-galactosidase staining kit (MedChemExpress, Cat# HY-K1089) following the manufacturer's instructions, except that the staining step was extended to 18 h at 37°C under ambient air.

17. In vivo radiotherapy via intratumoral injection

To establish 4T1R tumor models, 4T1R cells (approximately 2×10^6 cells/mouse) suspended in HBSS (50 μL; Gibco, Cat# C14175500CP) were subcutaneously injected into the flank of each mouse. Once tumors reached a volume of approximately 100 mm^3, mice were randomly assigned to eight groups (G1–G8). On day 0, mice in group G1 were intratumorally injected with normal saline (50 μL) containing Pluronic F-127 (0.5 wt%); mice in group G2 were intratumorally injected with normal saline (50 μL) containing TMTA (50 μM) and Pluronic F-127 (0.5 wt%); mice in group G3 were intratumorally injected with normal saline (50 μL) containing DIBO (75 μM) and Pluronic F-127 (0.5 wt%); and mice in group G4 were intratumorally injected with normal saline (50 μL) containing TMTA (50 μM), DIBO (75 μM), and Pluronic F-127 (0.5 wt%). Groups G5–G8 received formulations identical to G1–G4, respectively, but were additionally irradiated with 2 Gy X-ray on days 1 and 2. Mice were monitored until they met

the predefined ethical endpoints for euthanasia.

For bio-TEM analysis, some mice from groups G1 and G4 were euthanized on day 1. Their tumor tissues were excised, immediately trimmed into small pieces, and fixed in glutaraldehyde (10 mL, 2.5 vol%). The specimens were subsequently stored in glutaraldehyde (1.8 mL, 2.5 vol%) at 4°C. On the day of analysis, the samples were dehydrated through a graded ethanol series, embedded in epoxy resin, and sectioned to a thickness of 50–80 nm for bio-TEM examination.

For detecting in situ **UM-113** synthesis within the tumors, on day 1, some mice in groups G1 and G4 were euthanized. Their tumor tissues were collected and cut into small pieces in HBSS (1.0 mL) and mixed with HBSS (1.0 mL) containing collagenase II (2.0 mg/mL; Yeasen, Cat# 40508ES60), hyaluronidase (2.0 mg/mL; Yeasen, Cat# 20426ES60), and DNase I (40 U/mL; Yeasen, Cat# 10607ES15), followed by incubation for 2 h at 37°C. The dissociated tissues were crushed with a syringe plunger, strained through a 70 μm cell strainer, and rinsed with HBSS. The cells were collected by centrifugation at 1000 rpm for 6 min at 4°C and resuspended in a RBC lysis buffer (4.0 mL; Invitrogen, Cat# 00-4333-57), followed by incubation for 10 min at 4°C. The cells were washed twice with cold HBSS supplemented with HEPES (25 mM, pH 7.3; VivaCell, Cat# C3544-0100) and fetal bovine serum (2.0 vol%; Gibco, Cat# A5256701) to obtain cell suspensions. The cell suspensions were blocked with anti-CD16/CD32 (50 μL, 1:200 dilution; Invitrogen, Cat# 16-0161-85) for 1 h at 4°C. The cells were incubated with PE-Cy7-conjugated anti-CD45 (50 μL, 1:500 dilution; BioLegend, Cat# 103114) for 2 h at 4°C. The cells were washed twice with HBSS (100 μL) supplemented with HEPES (25 mM, pH 7.3; VivaCell, Cat# C3544-0100) and fetal bovine serum (2.0 vol%; Gibco, Cat# A5256701). The cells were fixed in an intracellular fixation buffer (100 μL, 1:2 dilution; Invitrogen, Cat# 00-8222-49) for 30 min at 4°C. The cells were washed twice with HBSS (100 μL) supplemented with HEPES (25 mM, pH 7.3; VivaCell, Cat# C3544-0100) and fetal bovine serum (2.0 vol%; Gibco, Cat# A5256701) and stored in the dark at 4°C until analysis by a flow cytometer using the AmCyan and PE-Cy7 channels.

To detect iodine content in tumors, mice in groups G3 and G4 were euthanized at 2–72 h post-injection. Tumor tissues were collected and digested using microwave heating in Teflon tubes containing tetramethylammonium hydroxide (1.0 mL, 5 wt%). The resulting solution was diluted with a mixture of isopropanol (1.5 vol%) and ammonia (7 mM) before ICP–MS analysis.

For detecting γH2AX expression, on day 3, some mice in groups G1 and G4 were euthanized. Their tumor tissues were collected and cell suspensions were obtained following the steps described above. The cell suspensions were blocked with anti-CD16/CD32 (50 μL, 1:200 dilution; Invitrogen, Cat# 16-0161-85) for 1 h at 4°C. The cells were incubated with PE-Cy7-conjugated anti-CD45 (50 μL, 1:500 dilution; BioLegend, Cat# 103114) for 2 h at 4°C. The cells were fixed and permeabilized using the Foxp3/transcription factor staining buffer set (Invitrogen, Cat# 00-5523-00) following the manufacturer's instructions. The cells were incubated with APC-conjugated anti-γH2AX

(50 μL, 1:100 dilution; BioLegend, Cat# 613416) for 2 h at 4°C. The cells were washed twice with HBSS supplemented with HEPES (25 mM, pH 7.3; VivaCell, Cat# C3544-0100) and fetal bovine serum (2.0 vol%; Gibco, Cat# A5256701) and stored in the dark at 4°C until analysis by a flow cytometer using the APC and PE-Cy7 channels.

For histological examination, tumor tissues and major organs were harvested at the treatment endpoint and washed with HBSS. The tissues were fixed in paraformaldehyde (4 wt%), dehydrated, embedded in paraffin, and sectioned at a thickness of 3–4 μm following standard protocols. The slices were stored at room temperature until used for H&E and IHC staining. For H&E staining, the slices were deparaffinized, rehydrated, and stained with an H&E stain kit (Nanjing Jiancheng Bioengineering Institute, Cat# D006-1-1) according to the manufacturer's guidelines. For IHC staining of Ki67, deparaffinized and rehydrated sections were subjected to antigen retrieval by microwaving in citrate antigen retrieval buffer (pH 6.0; ZSGB-BIO, Cat# ZLI-9064). The slices were permeabilized with Triton X-100 (0.3 vol%; TCI, Cat# P1775) for 15 min. The slices treated with H_2O_2 (0.3 wt%) for 10 min, and incubated with normal goat serum blocking solution (ZSGB-BIO, Cat# ZLI-9022) for 1 h. The slices were incubated with rabbit anti-Ki67 (1:200 dilution; Abcam, Cat# ab16667) overnight at 4°C. The bound antibodies were detected using a polymer-enhanced two-step immunohistochemistry detection system (ZSGB-BIO, Cat# PV-9001) and a diaminobenzidine kit (ZSGB-BIO, Cat# ZLI-9018) following the manufacturer's protocols. Nuclei were counterstained with hematoxylin (Nanjing Jiancheng Bioengineering Institute, Cat# D006-1-1).

18. In vivo radiotherapy via intravenous injection

To establish 4T1R tumor models, 4T1R cells (approximately $2×10^6$ cells/mouse) suspended in HBSS (50 μL; Gibco, Cat# C14175500CP) were subcutaneously injected into the flank of each mouse. Once tumors reached a volume of approximately 100 mm^3, mice were intravenously injected with normal saline (100 μL) containing TMTA (0–0.6 mM), DIBO (0–0.9 mM), and Pluronic F-127 (0–6.0 wt%). At 24 h and 48 h post-injection, tumors were locally irradiated with 2 Gy X-ray (each time). Mice were monitored until they met the predefined ethical endpoints for euthanasia.

For detecting in situ **UM-113** synthesis within the tumors, some mice were euthanized at 1–5 d post-injection. Their tumor tissues were collected and cut into small pieces in HBSS (1.0 mL) and mixed with HBSS (1.0 mL) containing collagenase II (2.0 mg/mL; Yeasen, Cat# 40508ES60), hyaluronidase (2.0 mg/mL; Yeasen, Cat# 20426ES60), and DNase I (40 U/mL; Yeasen, Cat# 10607ES15), followed by incubation for 2 h at 37°C. The dissociated tissues were crushed with a syringe plunger, strained through a 70 μm cell strainer, and rinsed with HBSS. The cells were collected by centrifugation at 1000 rpm for 6 min at 4°C and resuspended in a RBC lysis buffer (4.0 mL, 1:10 dilution; Invitrogen, Cat# 00-4300-54), followed by incubation for 10 min at 4°C. The cells were washed twice with cold HBSS supplemented with HEPES (25 mM, pH 7.3; VivaCell, Cat# C3544-0100) and fetal bovine serum (2.0 vol%; Gibco, Cat# A5256701) to obtain cell suspensions. The cell suspensions were blocked

with anti-CD16/CD32 (50 μL, 1:200 dilution; Invitrogen, Cat# 16-0161-85) for 1 h at 4°C. The cells were incubated with PE-Cy7-conjugated anti-CD45 (50 μL, 1:500 dilution; BioLegend, Cat# 103114) for 2 h at 4°C. The cells were washed twice with HBSS (100 μL) supplemented with HEPES (25 mM, pH 7.3; VivaCell, Cat# C3544-0100) and fetal bovine serum (2.0 vol%; Gibco, Cat# A5256701). The cells were fixed in an intracellular fixation buffer (100 μL, 1:2 dilution; Invitrogen, Cat# 00-8222-49) for 30 min at 4°C. The cells were washed twice with HBSS (100 μL) supplemented with HEPES (25 mM, pH 7.3; VivaCell, Cat# C3544-0100) and fetal bovine serum (2.0 vol%; Gibco, Cat# A5256701) and stored in the dark at 4°C until analysis by a flow cytometer using the KO525 and PC7 channels.

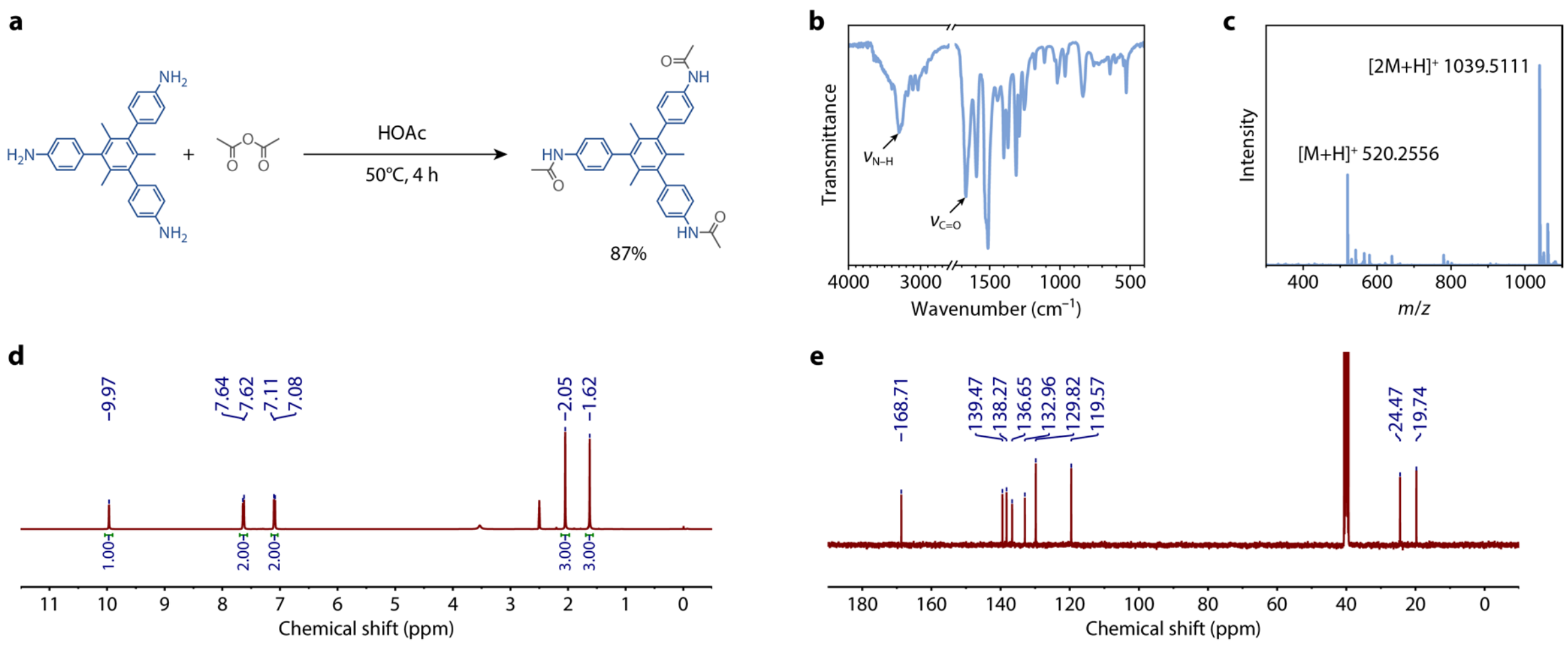

Figure S1. Synthesis of TMTA. (a) Synthetic route. (b) ATR–FTIR spectrum. (c) HRMS measured in CH_3OH. (d) ^1H NMR spectrum measured in DMSO-d_6. (e) ^{13}C NMR spectrum measured in DMSO-d_6.

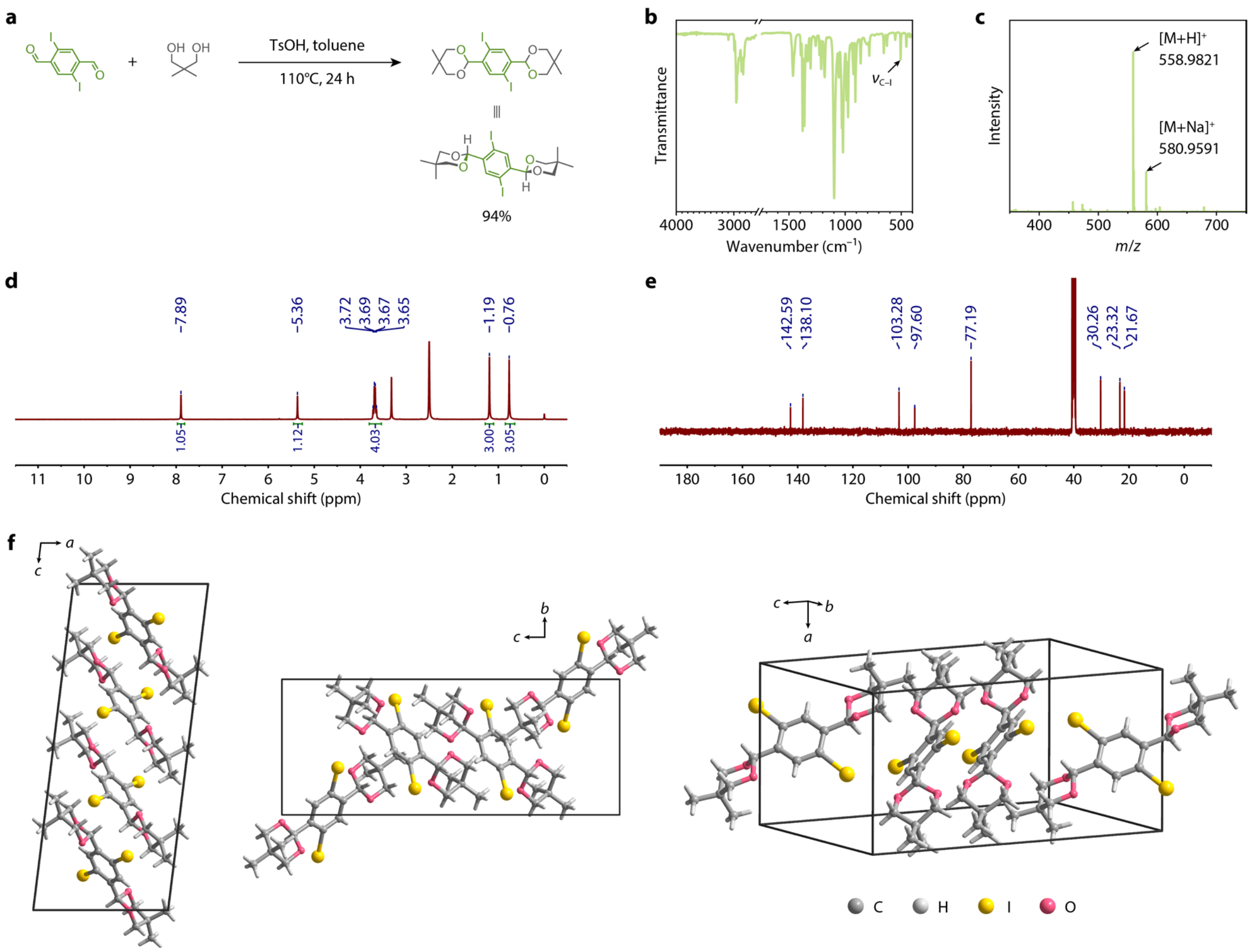

Figure S2. Synthesis of DIBO. (a) Synthetic route. (b) ATR–FTIR spectrum. (c) HRMS measured in CH_3OH. (d) ^{1}H NMR spectrum measured in DMSO-d_6. (e) ^{13}C NMR spectrum measured in DMSO-d_6. (f) Crystal structure. CCDC 2533880.

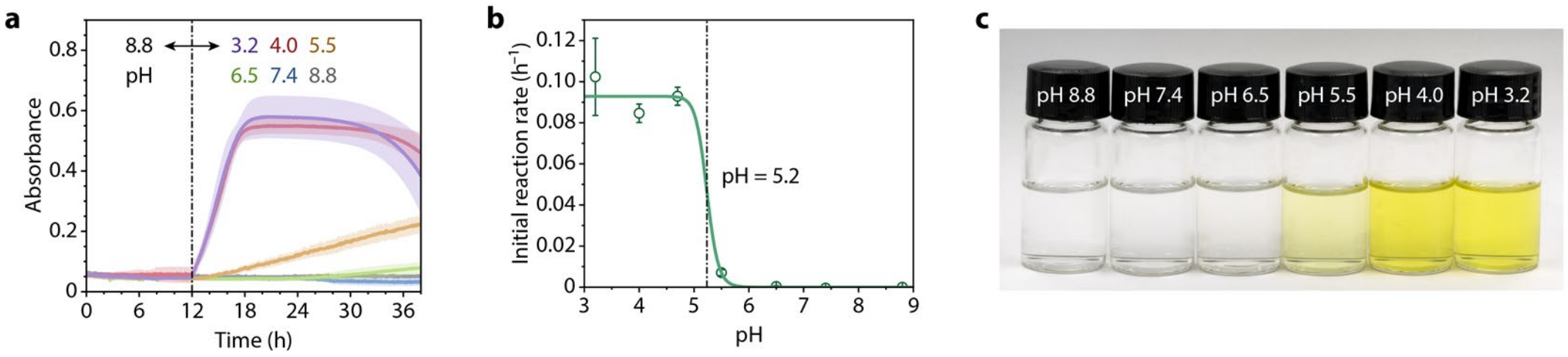

Figure S3. Kinetics of **UM-113** synthesis. (a) Time-dependent absorbance at 425 nm of reaction mixtures containing TMTA (50 μM) and DIBO (75 μM), esterase (50 U/L), and Pluronic F-127 (0.5 wt%) at pH 3.2–8.8. (b) Correlation between the initial reaction rate and pH. (c) Photograph of the reaction mixtures after 36 h of incubation. Data are presented as mean ± SD from 4 independent experiments (a, b) and fitted using a logistic equation (b).

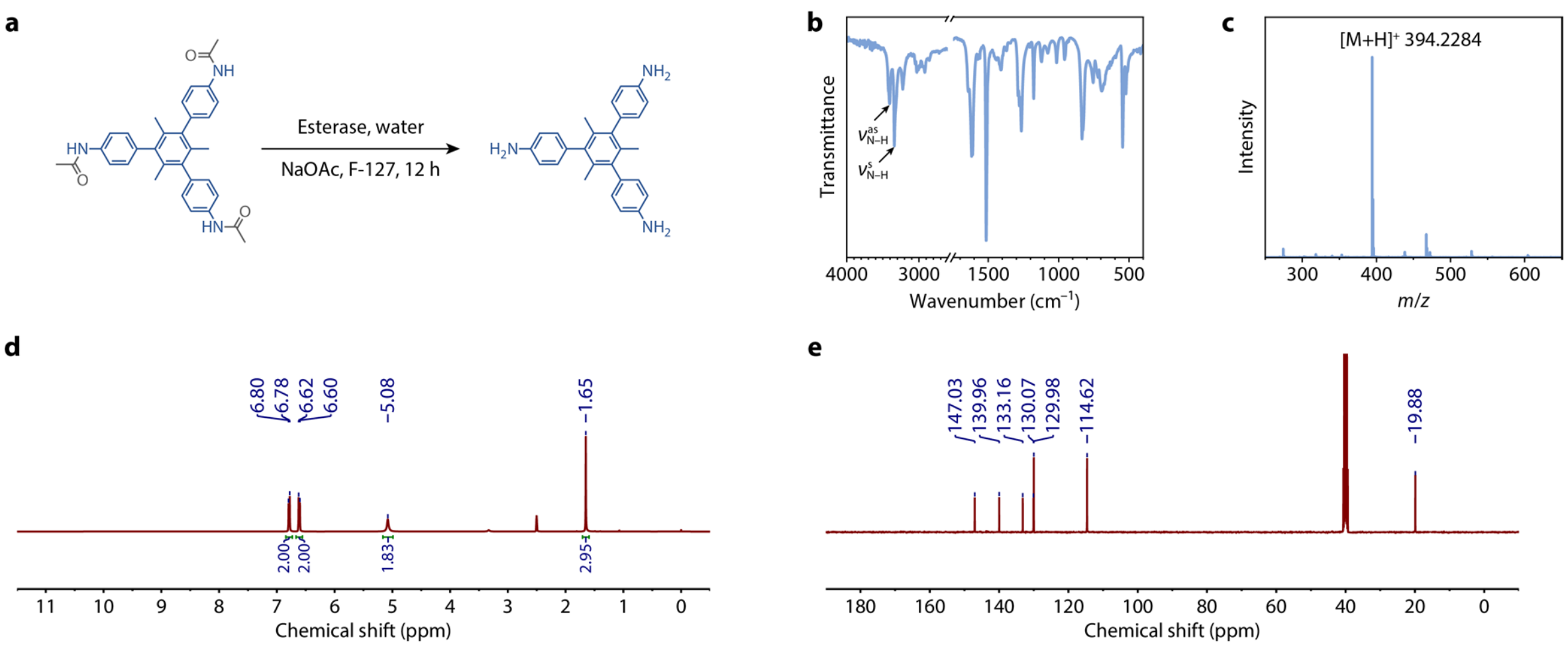

Figure S4. Enzyme-catalyzed hydrolysis of TMTA (50 μM) in an aqueous NaOAc solution (0.1 M, pH 8.8) in the presence of Pluronic F-127 (0.5 wt%) and esterase (50 U/L) at 37°C. (a) Synthetic route. (b) ATR–FTIR spectrum. (c) HRMS measured in CH_3OH. (d) ^1H NMR spectrum measured in DMSO-d_6. (e) ^{13}C NMR spectrum measured in DMSO-d_6.

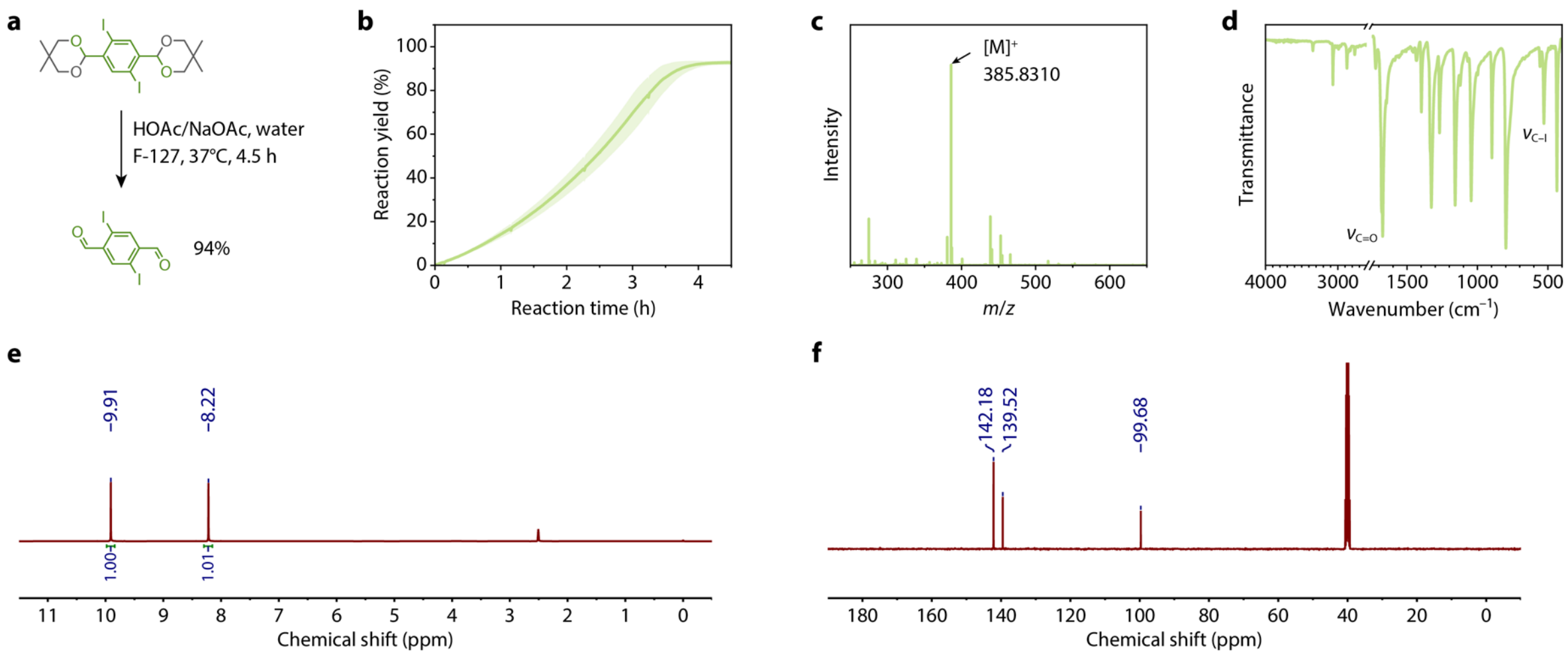

Figure S5. Acid-catalyzed hydrolysis of DIBO (75 μM) in an aqueous HOAc/NaOAc solution (0.2 M, pH 4.7) in the presence of Pluronic F-127 (0.5 wt%) at 37°C. (a) Synthetic route. (b) Hydrolysis reaction kinetics by detecting absorbance. Data are expressed as mean ± SD; n = 4 independent experiments. (c) HRMS measured in acetonitrile. (d) ATR–FTIR spectrum. (e) ^{1}H NMR spectrum measured in DMSO-d_6. (f) ^{13}C NMR spectrum measured in DMSO-d_6.

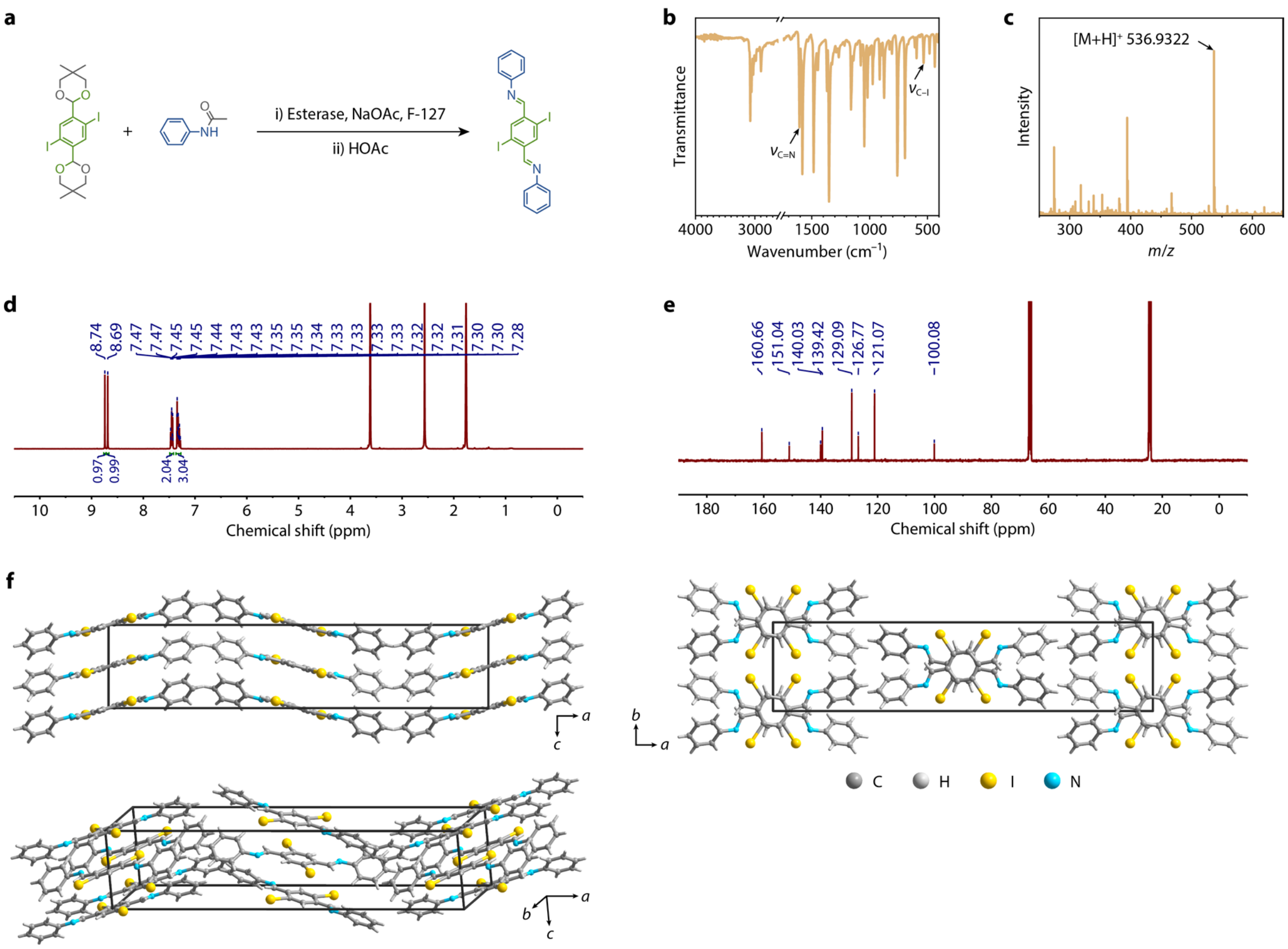

Figure S6. Synthesis of the diimine via esterase- and acid-catalyzed cascade reactions. (a) Synthetic route. (b) ATR–FTIR spectra. (c) HRMS measured in THF. (d) ^{1}H NMR spectrum measured in THF-d_8. (e) ^{13}C NMR spectrum measured in THF-d_8. (f) Crystal structure. CCDC 2533881.

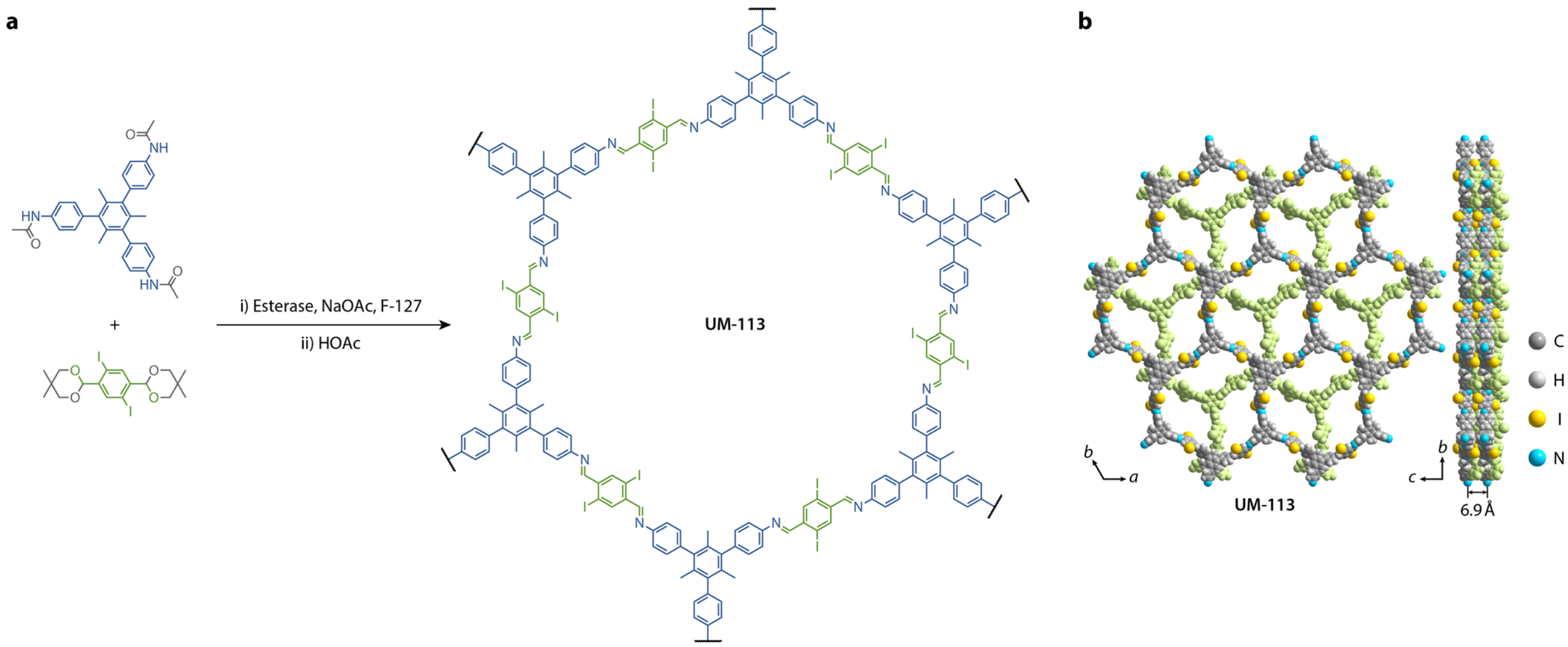

Figure S7. Synthesis (a) and the proposed AB-stacking mode (b) of **UM-113**.

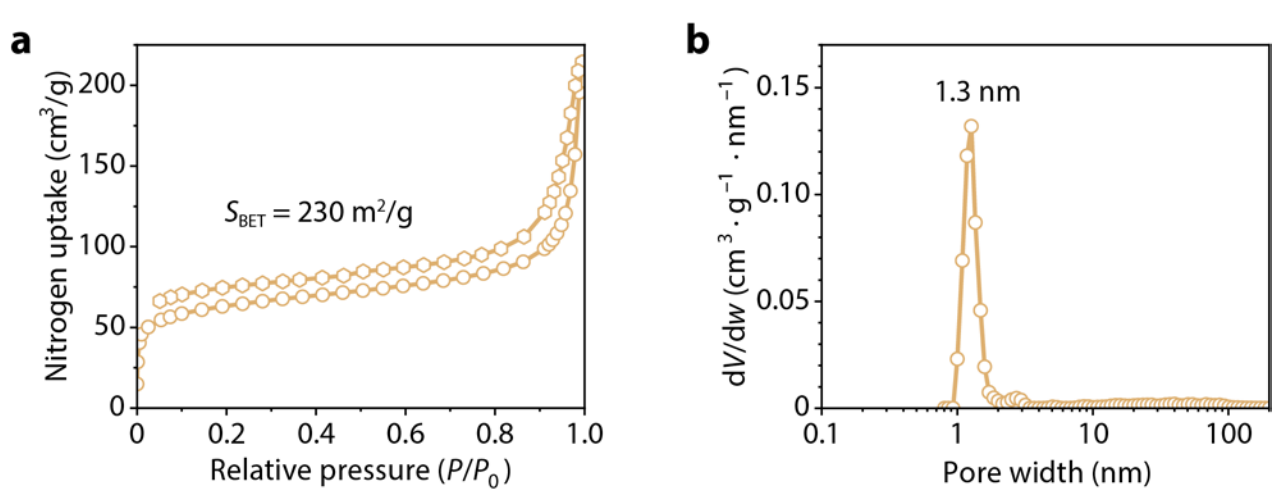

Figure S8. N_2 adsorption–desorption isotherms (a) measured at 77 K and pore size distribution plot (b) of **UM-113**.

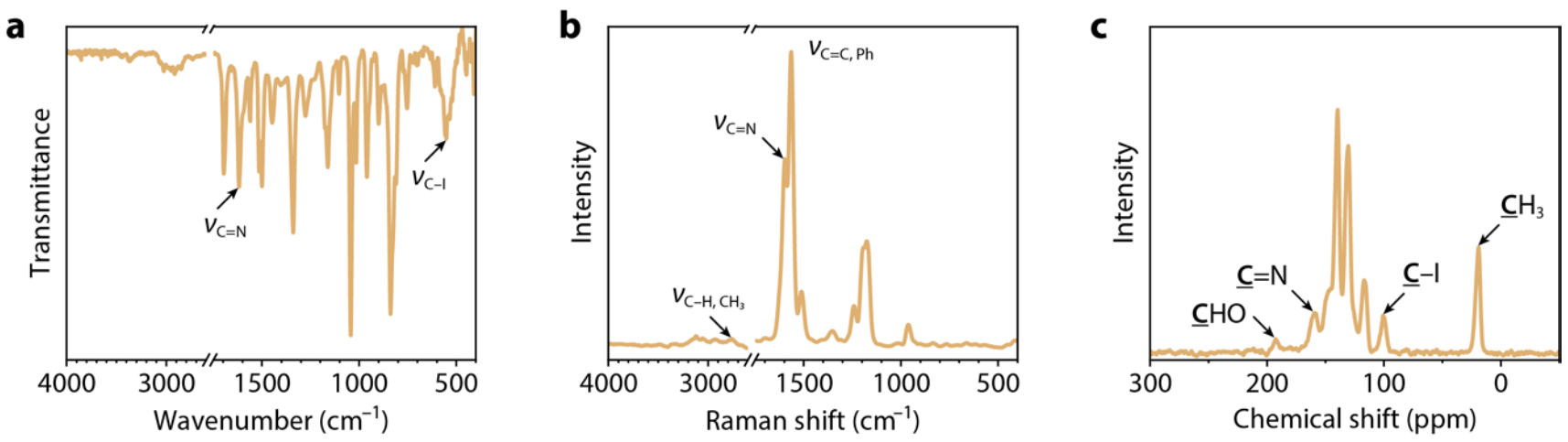

Figure S9. ATR–FTIR (a), Raman (b), and ssNMR (c) spectra of **UM-113**.

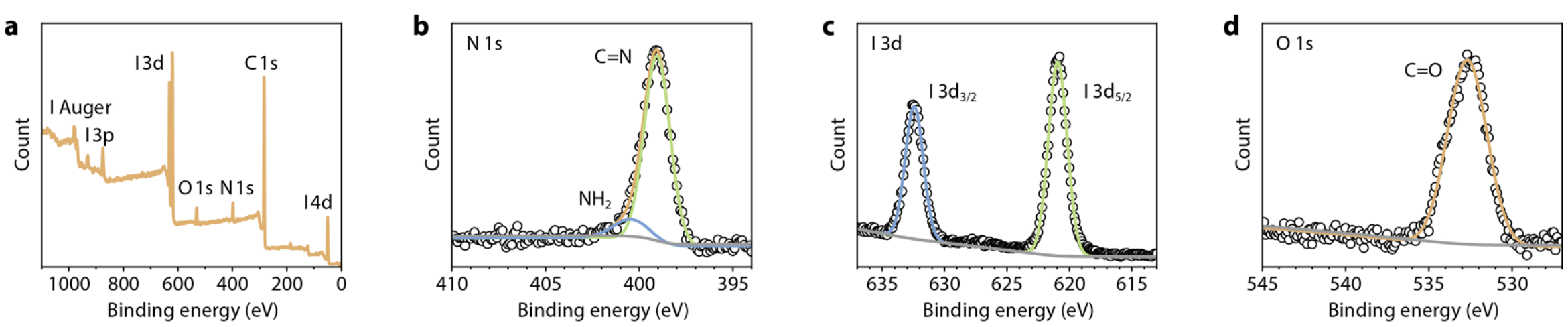

Figure S10. XPS analysis of **UM-113**. (a) XPS survey spectrum. (b–d) High-resolution XPS data in the N 1s (b), I 3d (c), and O 1s (d) regions.

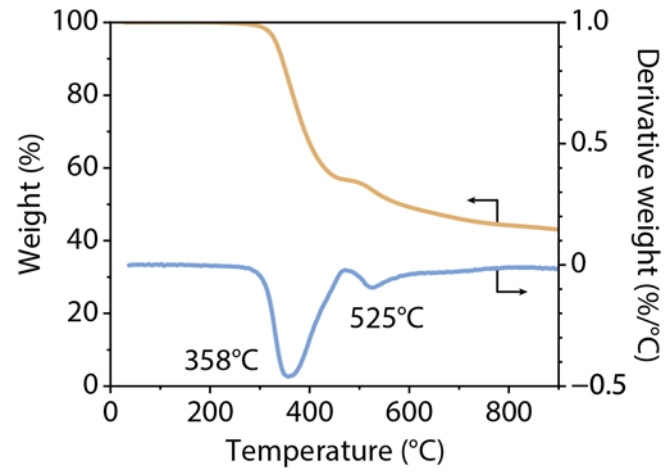

Figure S11. Thermogravimetric (brown) and the corresponding differential thermogravimetric (blue) curves of **UM-113**.

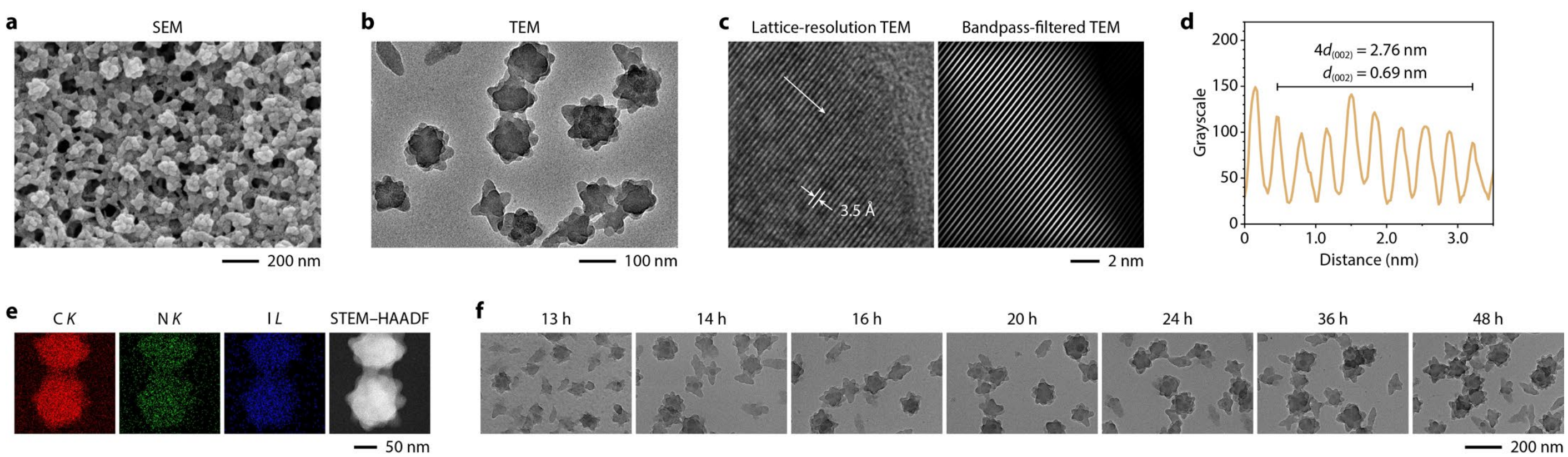

Figure S12. Electron microscopy characterization. (a) SEM image of **UM-113** nanoparticles. (b) TEM image of **UM-113** nanoparticles. (c) Lattice-resolution TEM image of **UM-113** nanoparticles and its bandpass-filtered images with enhanced contrast. (d) Interplanar spacing determined by measuring the average distance between adjacent lattice fringes. (e) Elemental mapping of **UM-113** nanoparticles. (f) Morphology of the nanoparticles isolated from the synthetic system at different time points.

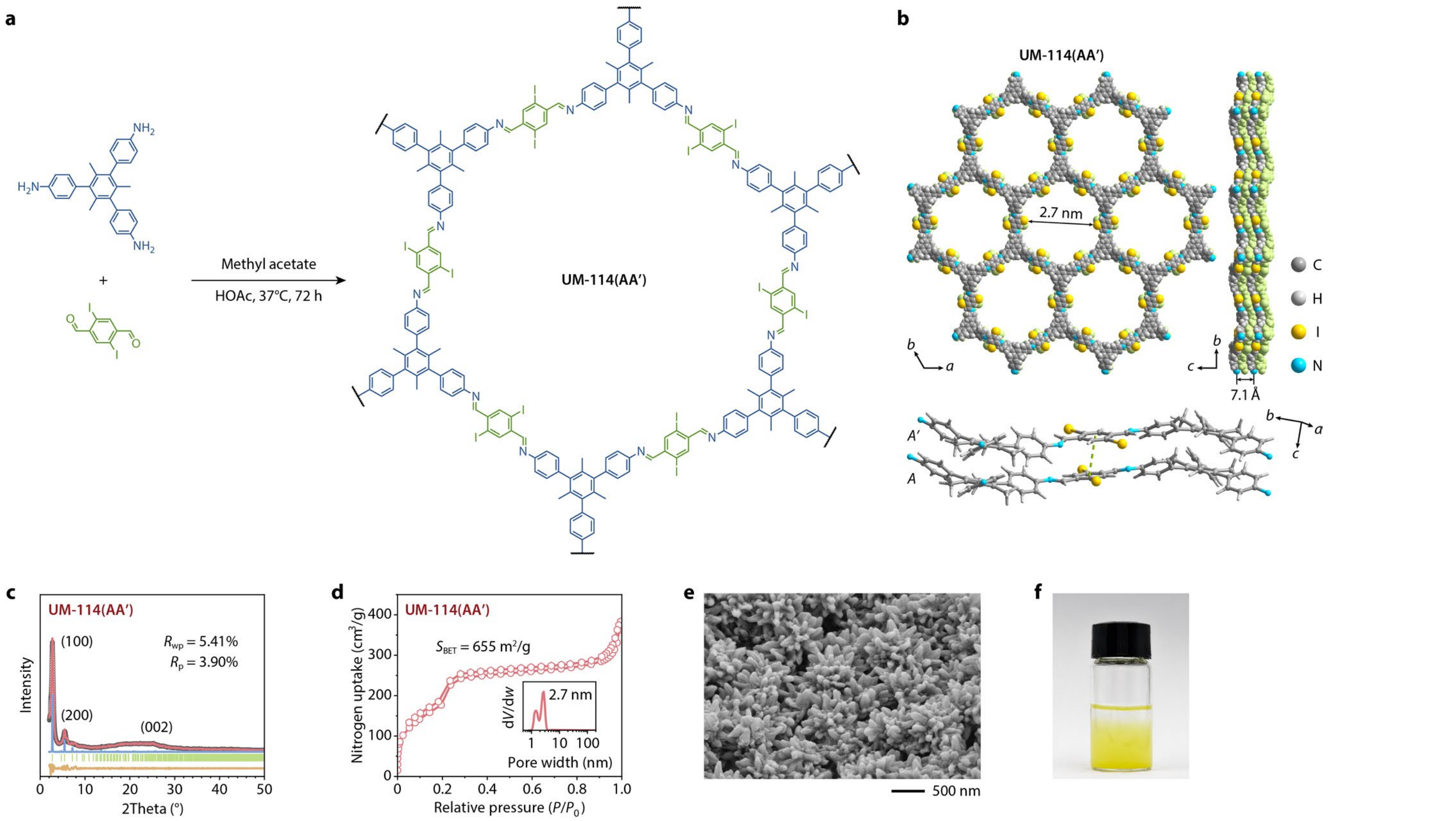

Figure S13. Synthesis and characterization of **UM-114(AA')**. (a) Synthetic scheme. (b) Proposed AA'-stacking model. (c) PXRD analysis showing the experimental patterns (black), Pawley-refined patterns (red), simulated patterns (blue), difference plots (brown), and Bragg positions (green). (d) N_2 adsorption–desorption isotherms measured at 77 K. Inset: Pore size distribution plot. (e) SEM image. (f) Digital photograph of **UM-114(AA')** dispersed in DPBS showing nanoparticle aggregation and sedimentation.

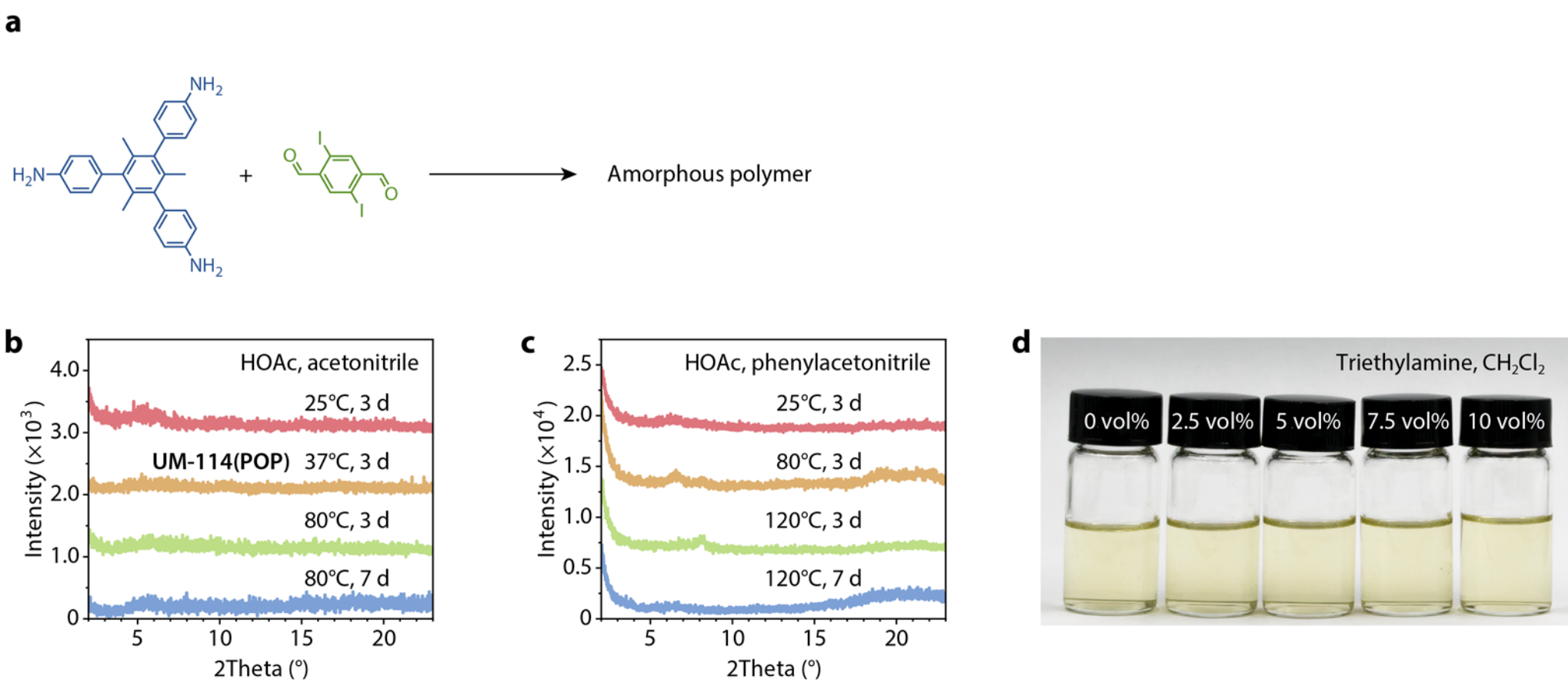

Figure S14. Synthesis of amorphous polymers. (a) Synthetic scheme. (b, c) PXRD patterns of amorphous polymers synthesized in acetonitrile (b) and phenylacetonitrile (c) with HOAc at 25–120°C for 3–7 d. (d) Photograph of a dichloromethane solution containing 2,5-diiodoterephthalaldehyde (2.1 mM), 2,4,6-tris(4-aminophenyl)-1,3,5-trimethylbenzene (1.4 mM), and triethylamine (0–10 vol%) after reacting at 40°C for 7 d.

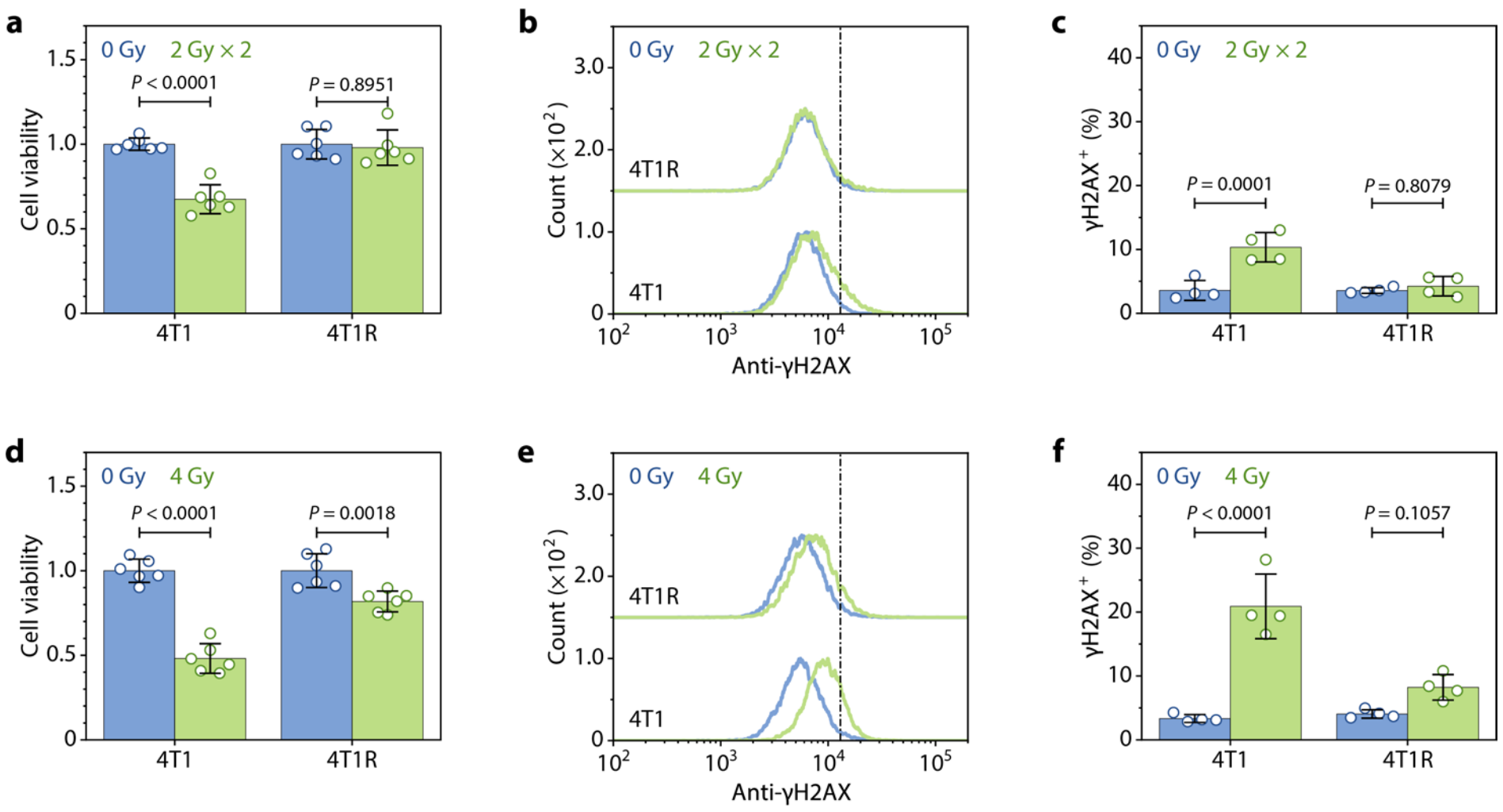

Figure S15. Construction of radioresistant 4T1R cell lines. (a) Cell viability assays of 4T1 and 4T1R cells following fractionated exposures of 2 Gy/d for 2 d. Interaction $p = 0.0002$. (b, c) Flow cytometric analysis (b) and the corresponding quantification (c) of γH2AX immunofluorescence staining of 4T1 and 4T1R cells following fractionated exposures of 2 Gy/d for 2 d. Interaction $p = 0.0026$. (d) Cell viability assays of 4T1 and 4T1R cells following a single exposure of 4 Gy. Interaction $p < 0.0001$. (e, f) Flow cytometric analysis (e) and the corresponding quantification (f) of γH2AX immunofluorescence staining of 4T1 and 4T1R cells following a single exposure of 4 Gy. Interaction $p = 0.0004$. Data are expressed as mean ± SD, $n = 6$ technical replicates (a, d) or 4 biological replicates (c, f). Statistical significance is calculated by two-way ANOVA with Šídák's multiple comparison test.

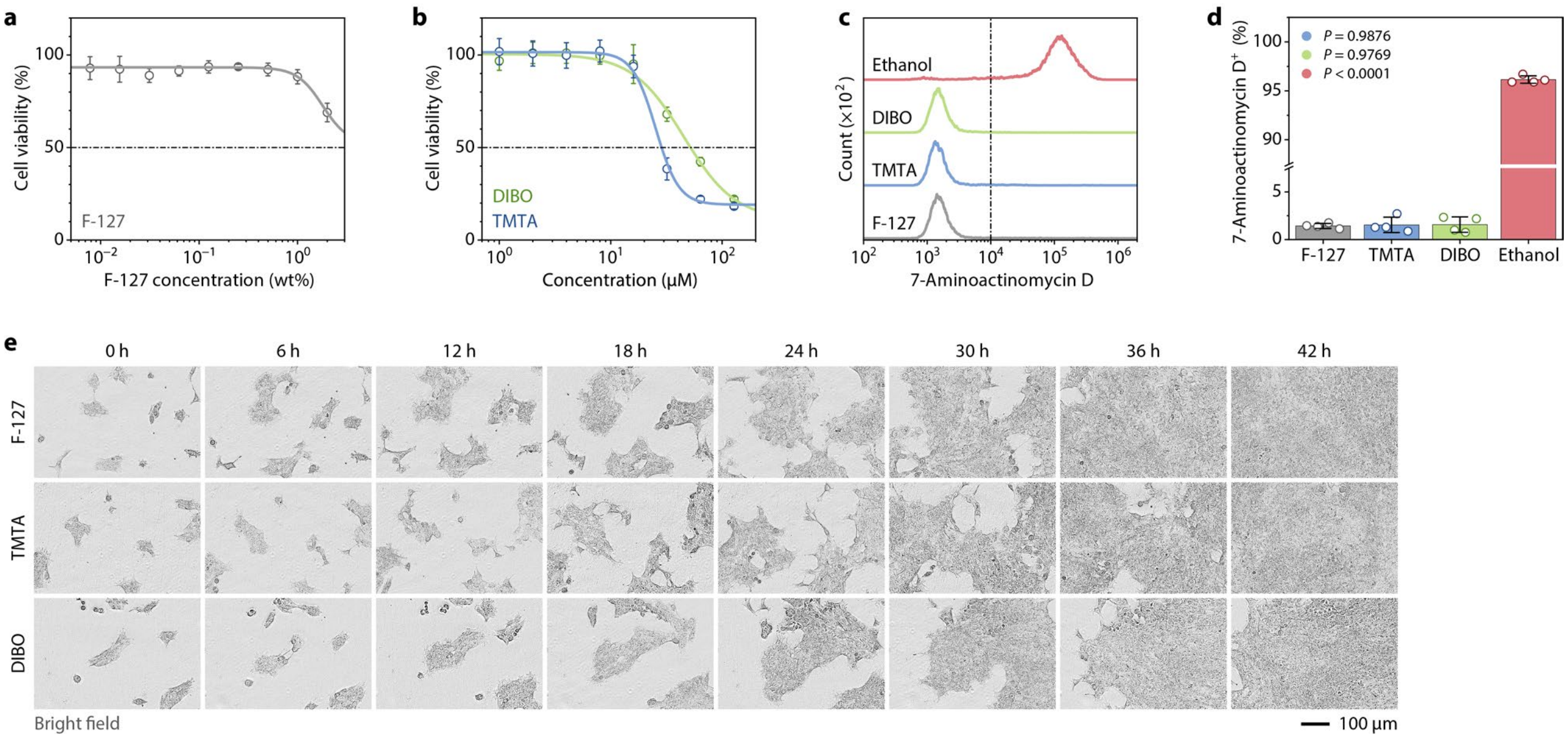

Figure S16. Cytotoxicity of Pluronic F-127, TMTA, and DIBO. (a, b) CCK-8 viability assays of 4T1R cells treated with Pluronic F-127 (a), TMTA (b), or DIBO (b) for 24 h. Data are expressed as mean ± SD and fitted using a logistic equation, *n* = 6 technical replicates. (c, d) Representative flow cytometric analysis (c) and 7-aminoactinomycin D$^+$ dead cell percentage (d) of 4T1R cells treated with Pluronic F-127 (0.04 wt%, 24 h), TMTA (4.0 μM, 24 h), DIBO (6.0 μM, 24 h), or ethanol (75 vol%, 12 h). Data are expressed as mean ± SD, *n* = 4 biological replicates. Statistical significance was calculated by one-way ANOVA with Dunnett's multiple comparison test. (e) Real-time live-cell imaging for monitoring cellular mitosis of 4T1R cells treated with Pluronic F-127 (0.04 wt%), TMTA (4.0 μM), or DIBO (6.0 μM) for 0–42 h.

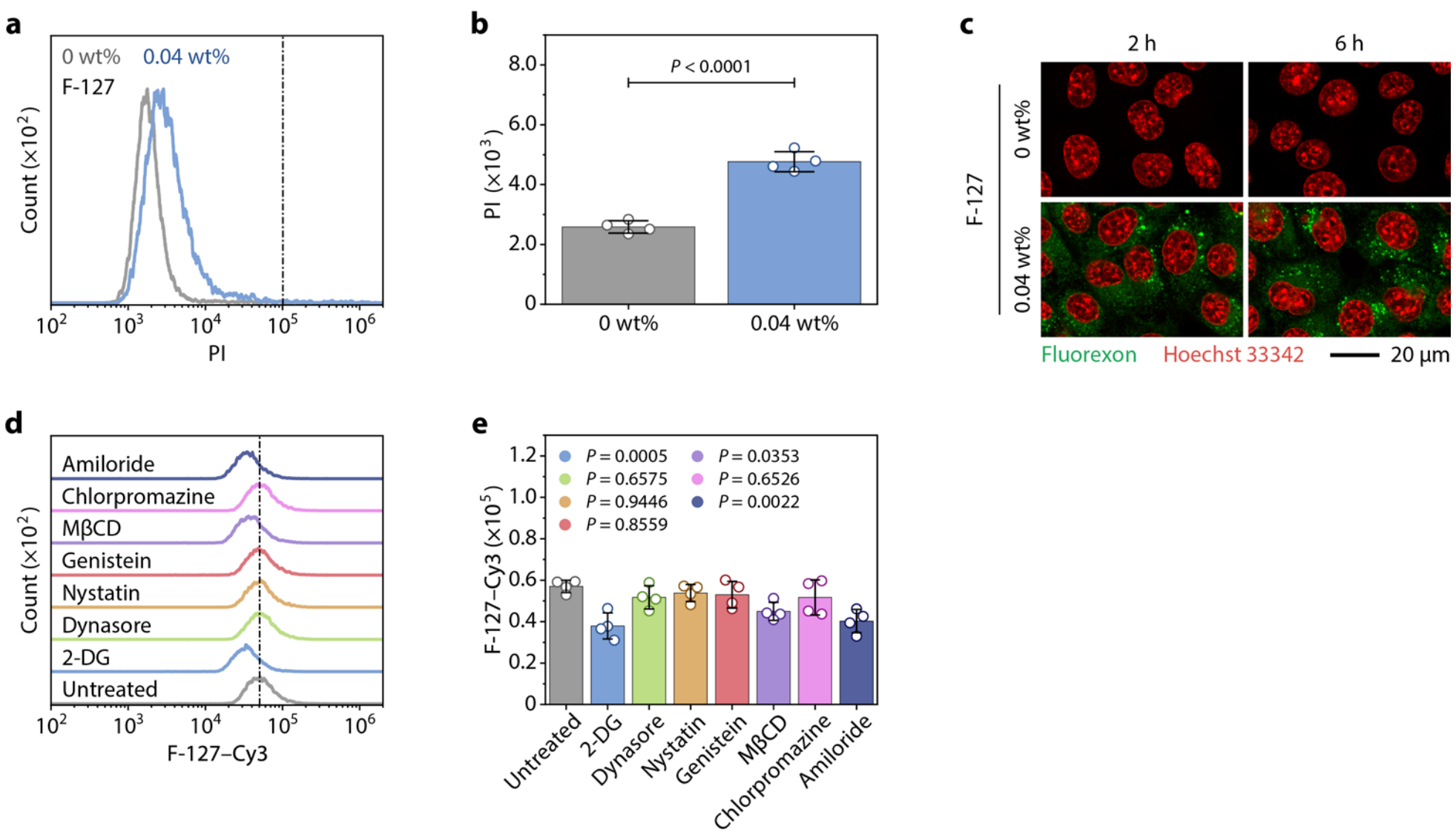

Figure S17. Pluronic F-127–mediated cytomembrane permeabilization and pinocytosis. (a, b) Flow cytometric analysis (a) and MFI of living cells (b) after incubation of 4T1R cells with Pluronic F-127 (0–0.04 wt%) and PI (5.0 μM) for 2 h. (c) Confocal laser scanning fluorescence microscopy images of 4T1R cells treated with Pluronic F-127 (0.04 wt%) and fluorexon (1.0 μM) for 2–6 h. (d, e) Flow cytometric analysis (d) and the corresponding MFI (e) of 4T1R cells incubated with Cy3-labeled Pluronic F-127 (0.04 wt%) in the presence of 2-DG (20 mM), dynasore (75 μM), nystatin (20 μM), genistein (100 μM), MβCD (5.0 mg/mL), chlorpromazine (50 μM), or amiloride (0.2 mM). Data are expressed as mean ± SD, n = 4 biological replicates. Statistical significance is determined using unpaired two-tailed t test (b) and one-way ANOVA with Dunnett's post-hoc test (e).

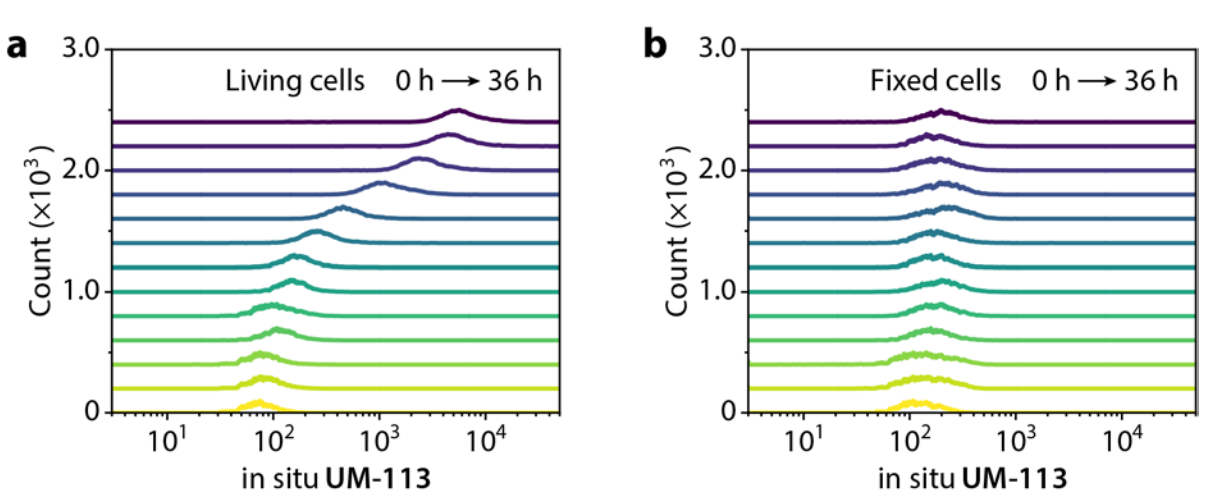

Figure S18. Flow cytometric analysis of the living (a) and fixed (b) 4T1R cells treated with TMTA (4.0 μM), DIBO (6.0 μM), and Pluronic F-127 (0.04 wt%) for 0–36 h.

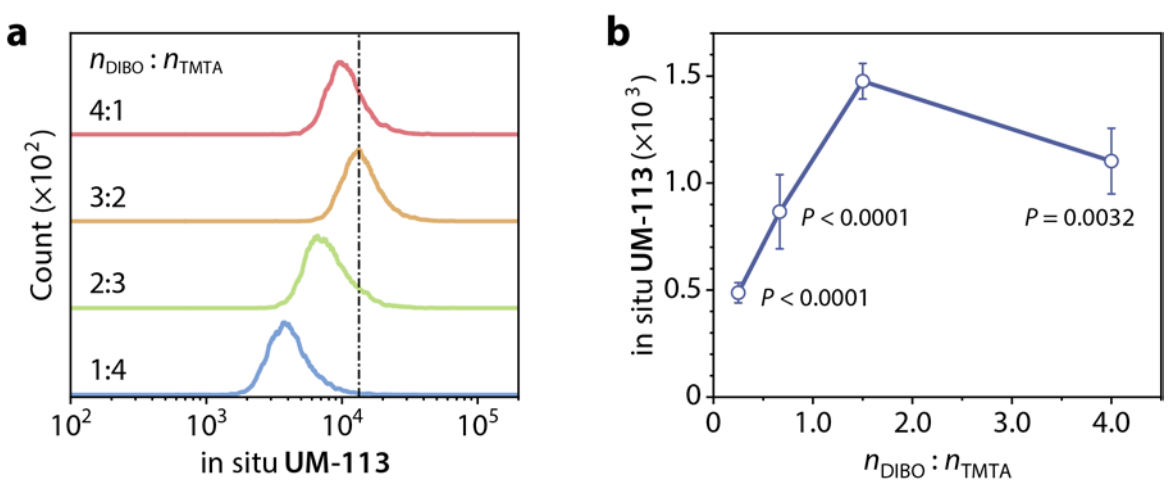

Figure S19. Flow cytometric analysis (a) and the corresponding MFI (b) of 4T1R cells incubated with DIBO (2.0, 4.0, 6.0, and 8.0 μM) and TMTA (8.0, 6.0, 4.0, and 2.0 μM) in the presence of Pluronic F-127 (0.04 wt%) for 24 h. Data are expressed as mean ± SD, n = 4 biological replicates. Statistical significance was calculated by one-way ANOVA followed by Dunnett's multiple comparison test.

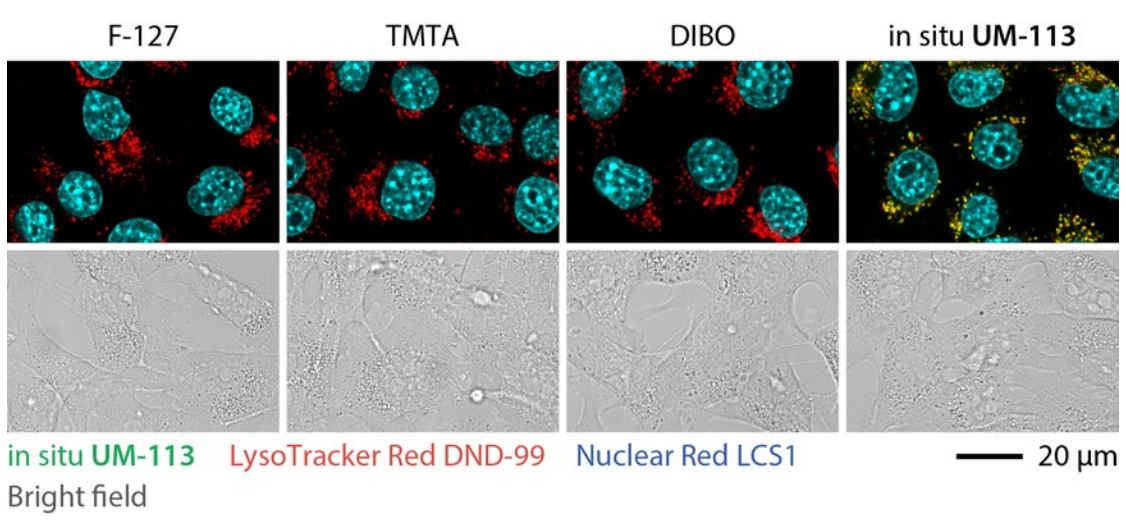

Figure S20. Confocal laser scanning fluorescence micrographs of 4T1R cells treated with TMTA (4.0 μM), DIBO (6.0 μM), and Pluronic F-127 (0.04 wt%) for 24 h.

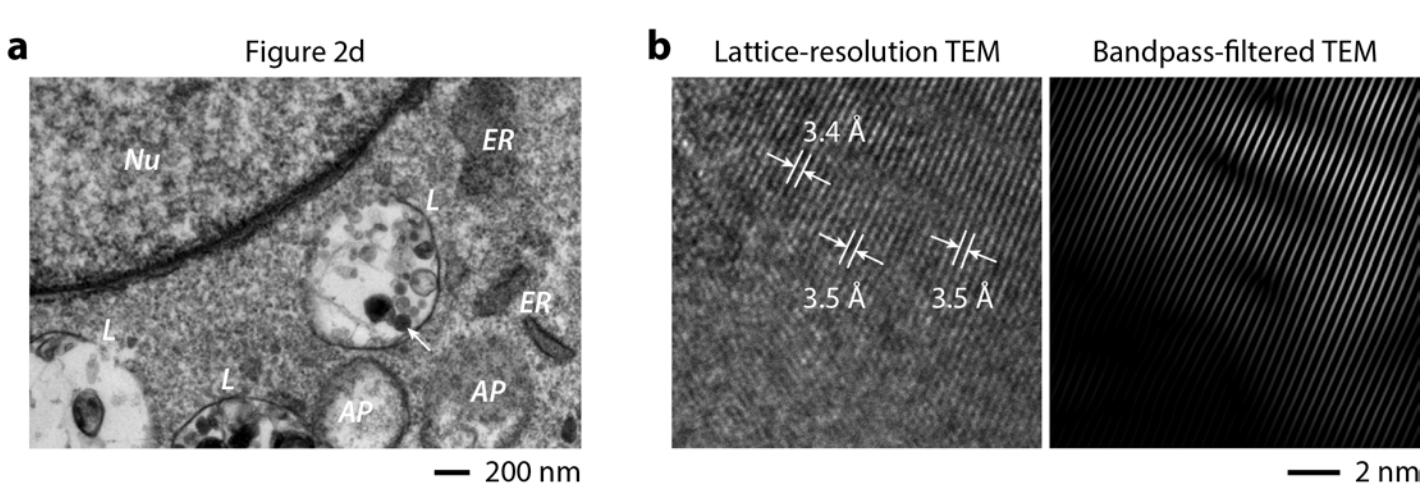

Figure S21. TEM images of 4T1R cells showing in situ **UM-113** nanoparticles within lysosomes. (a) Enlarged view of Figure 2d. (b) Lattice-resolution TEM image of in situ **UM-113** nanoparticles inside 4T1R cells and its bandpass-filtered images with enhanced contrast.

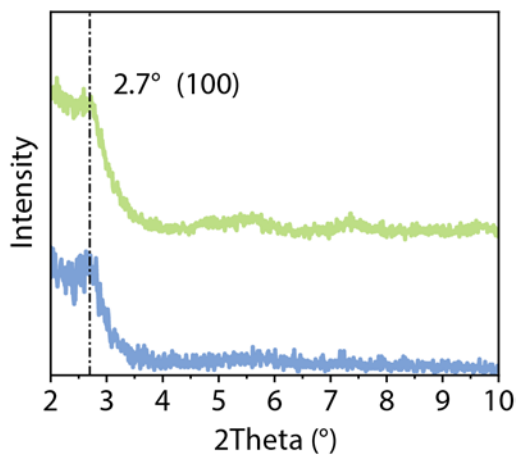

Figure S22. PXRD patterns of in situ **UM-113** extracted from 4T1R cells following 24 h of treatment with TMTA (4.0 μM), DIBO (6.0 μM), and Pluronic F-127 (0.04 wt%) via density gradient centrifugation (green) or triethylamine precipitation (blue).

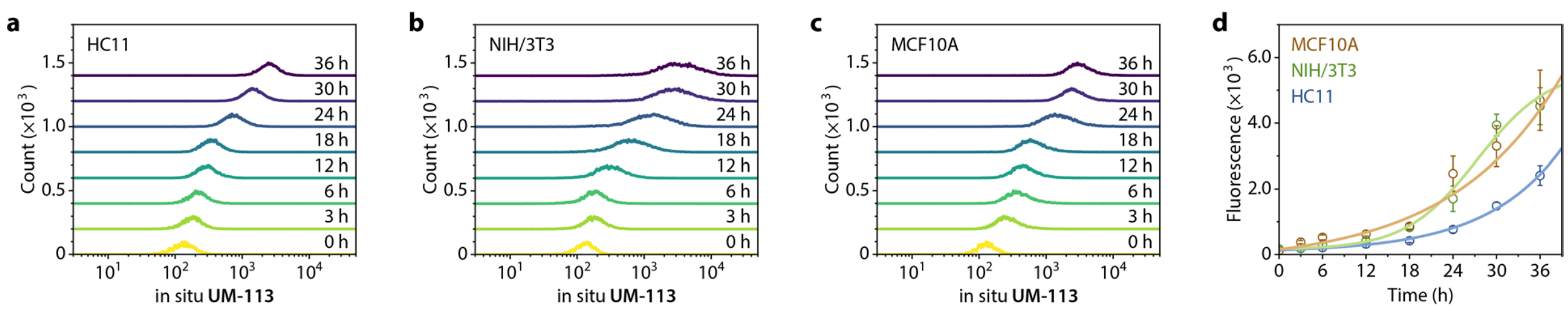

Figure S23. COF synthesis inside healthy tissue cells. (a–d) Flow cytometric analysis (a–c) and the corresponding MFI (d) of HC11 (a), NIH/3T3 (b), MCF10A (c) cells incubated with TMTA (4.0 μM), DIBO (6.0 μM), and Pluronic F-127 (0.04 wt%) for 0–36 h. Data are expressed as mean ± SD and fitted with the Logistic model, n = 4 biological replicates.

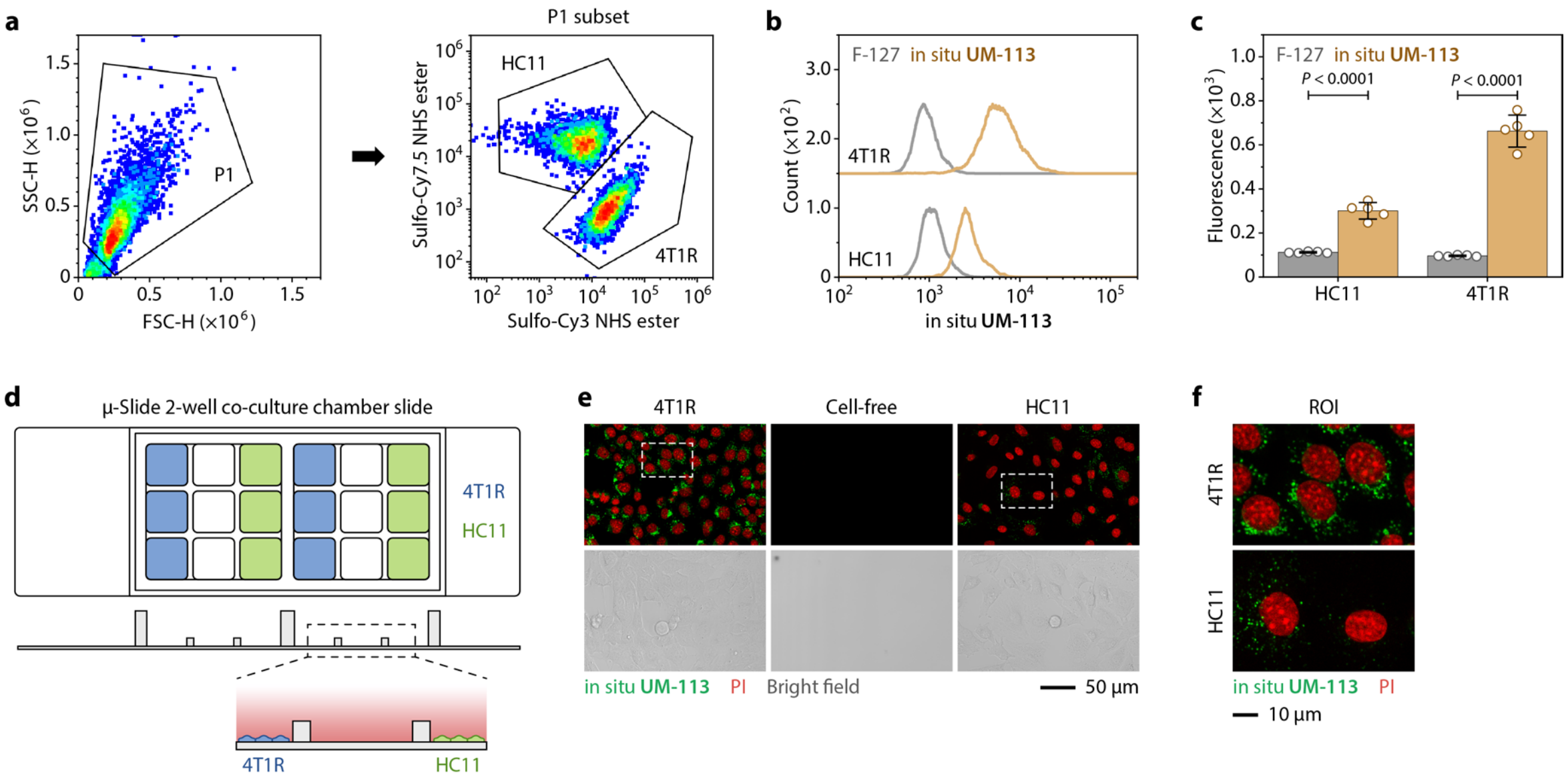

Figure S24. Intracellular COF formation in HC11 and 4T1R co-cultures treated with TMTA (4.0 μM), DIBO (6.0 μM), and Pluronic F-127 (0.04 wt%) for 24 h. (a–c) Flow cytometric detection of in situ **UM-113** synthesis, showing the gating strategy for cell isolation (a), flow cytometric plots (b), and the corresponding MFI (c). Data are expressed as mean ± SD, n = 5 biological replicates. Statistical significance is calculated by two-way ANOVA with Šídák's multiple comparison test. Interaction $p < 0.0001$. (d–f) Confocal laser scanning fluorescence imaging of in situ **UM-113** synthesis using μ-slide 2-well co-culture chamber slides (d), with representative images (e) and magnified ROI images (f).

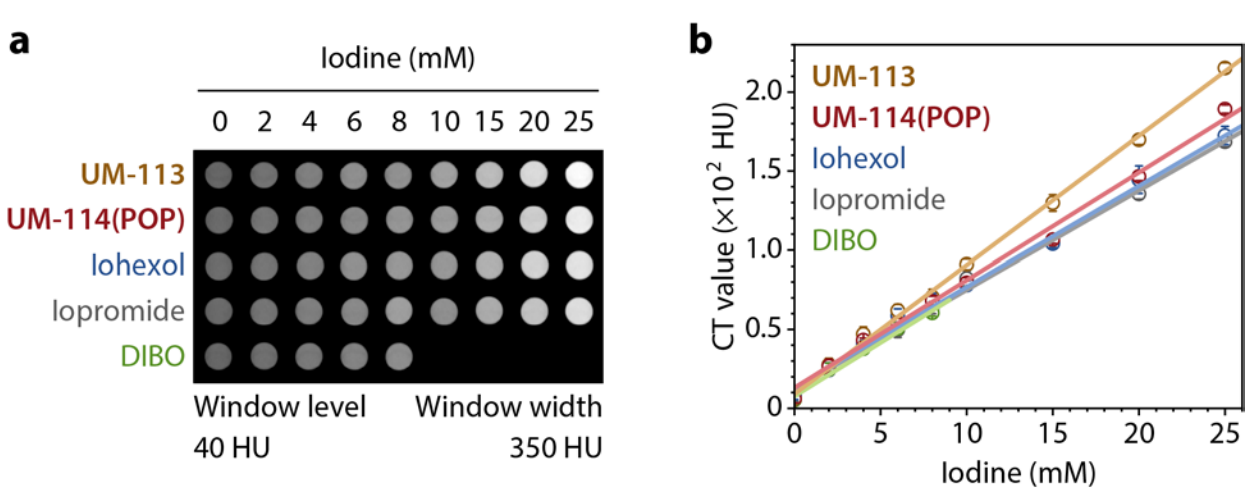

Figure S25. CT images (a) and the corresponding attenuation plots (b) of **UM-113**, **UM-114(POP)**, iohexol, iopromide, and DIBO in DPBS. Data are expressed as mean ± SD and fitted with the linear model, n = 4 independent experiments.

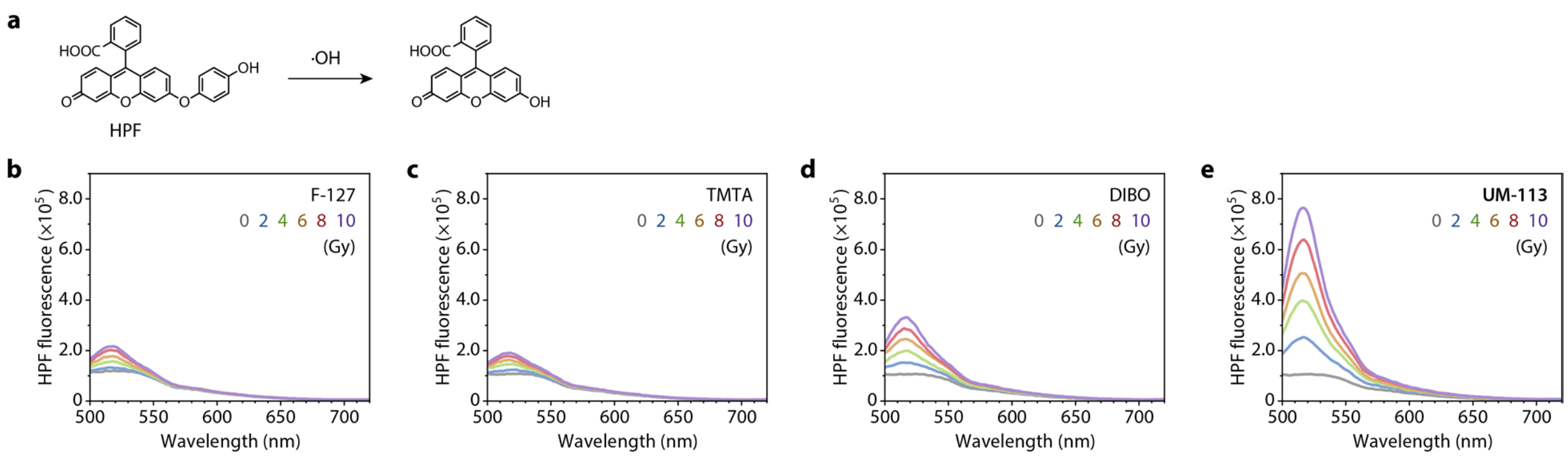

Figure S26. Detection of X-ray-induced ·OH generation using HPF. (a) Testing principle. (b–e) Fluorescence spectra of HPF in the presence of Pluronic F-127 (1.0 wt%), TMTA (100 μM), DIBO (150 μM), and **UM-113** (300 μM, iodine equiv.) upon X-ray irradiation (0–10 Gy). Excitation wavelength λ_{ex} = 455 nm.

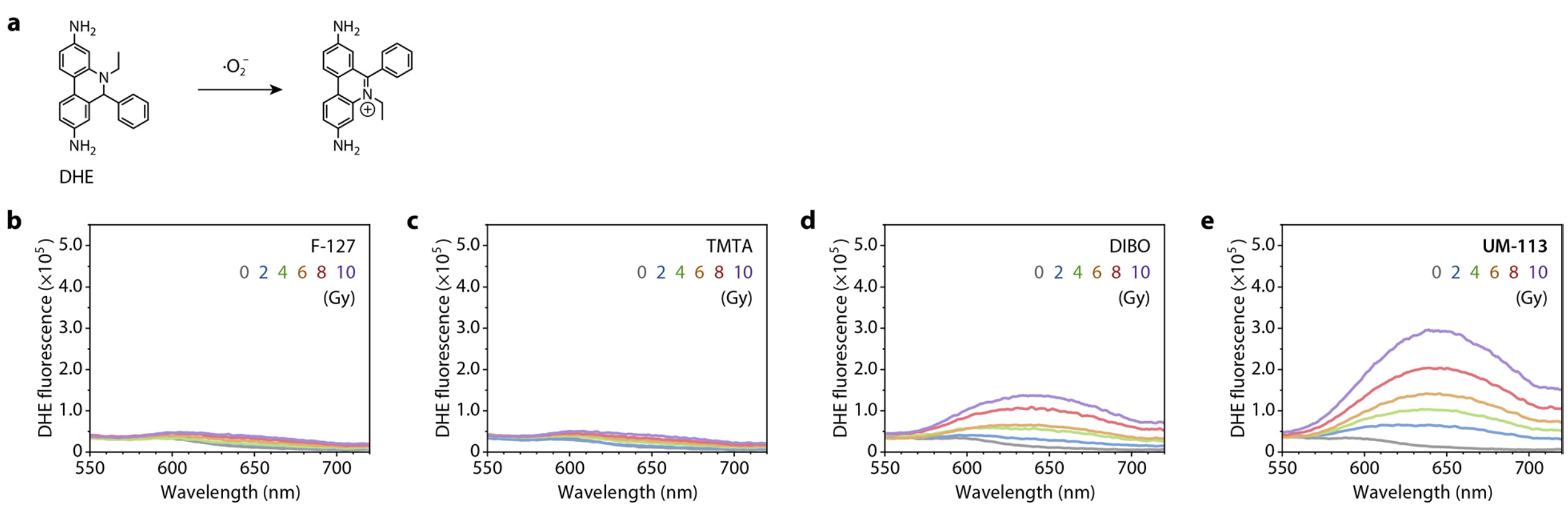

Figure S27. Detection of X-ray-induced $\cdot O_2^-$ generation using DHE. (a) Testing principle. (b–e) Fluorescence spectra of DHE in the presence of Pluronic F-127 (1.0 wt%), TMTA (100 μM), DIBO (150 μM), and **UM-113** (300 μM, iodine equiv.) upon X-ray irradiation (0–10 Gy). Excitation wavelength λ_{ex} = 500 nm.

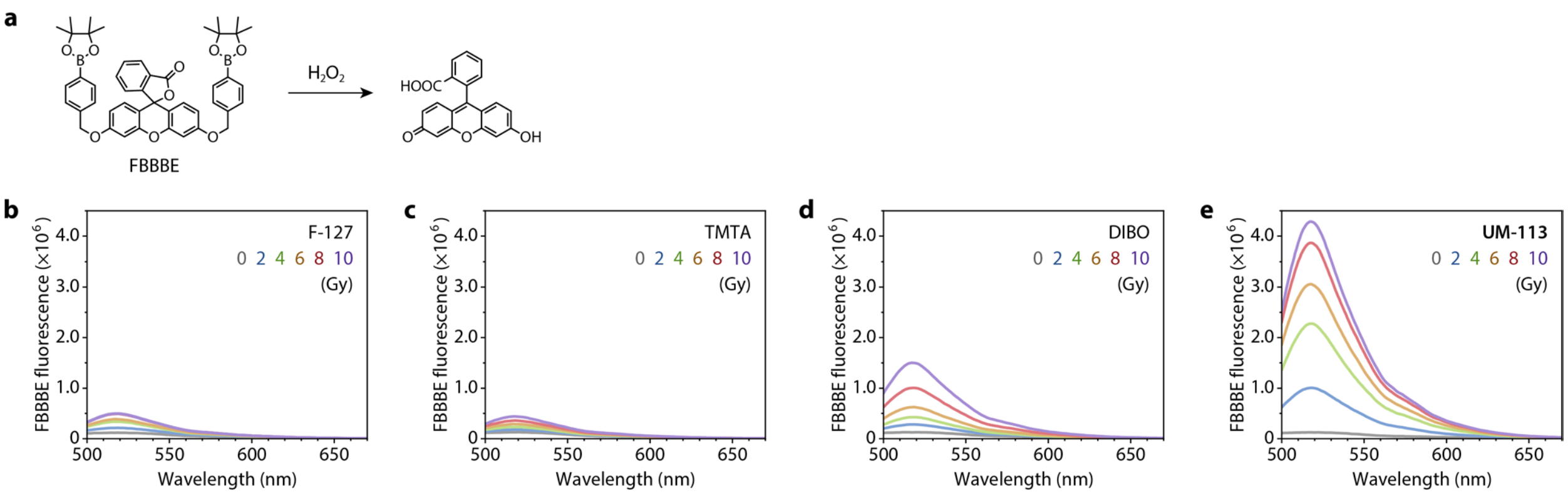

Figure S28. Detection of X-ray-induced H_2O_2 generation using FBBBE. (a) Testing principle. (b–e) Fluorescence spectra of FBBBE in the presence of Pluronic F-127 (1.0 wt%), TMTA (100 μM), DIBO (150 μM), and **UM-113** (300 μM, iodine equiv.) upon X-ray irradiation (0–10 Gy). Excitation wavelength λ_{ex} = 455 nm.

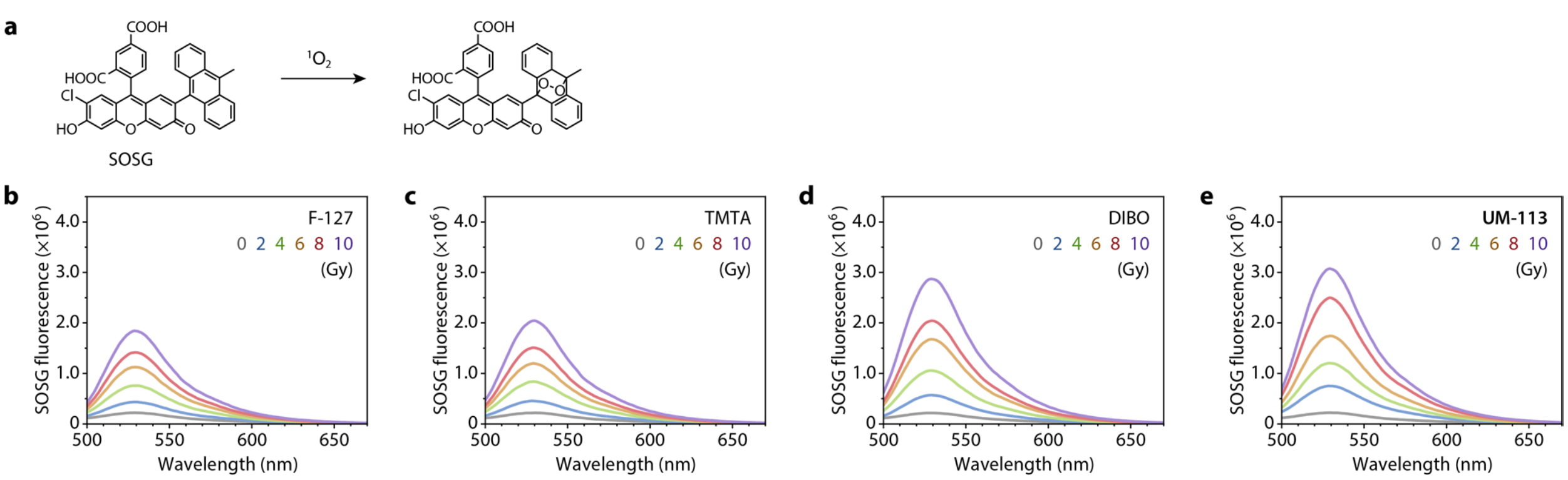

Figure S29. Detection of X-ray-induced $^1O_2$ generation using SOSG. (a) Testing principle. (b–e) Fluorescence spectra of SOSG in the presence of Pluronic F-127 (1.0 wt%), TMTA (100 μM), DIBO (150 μM), and **UM-113** (300 μM, iodine equiv.) upon X-ray irradiation (0–10 Gy). Excitation wavelength λ_{ex} = 455 nm.

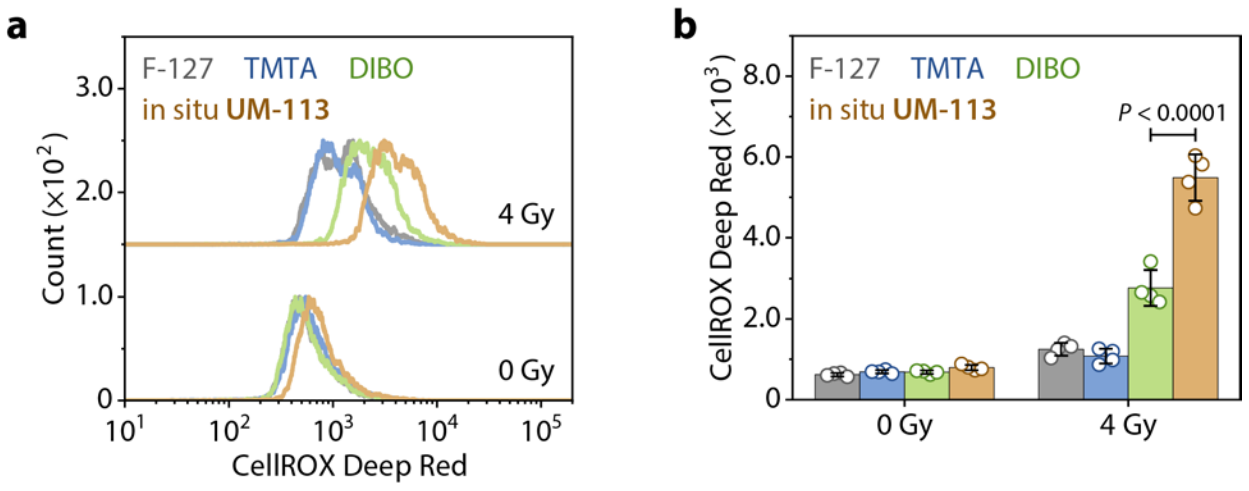

Figure S30. Flow cytometric analysis (a) and the corresponding quantification (b) of CellROX Deep Red staining for the detection of total ROS in 4T1R cells treated with TMTA (4.0 μM), DIBO (6.0 μM), and Pluronic F-127 (0.04 wt%) for 24 h followed by X-ray irradiation (0–4 Gy). Data are expressed as mean ± SD, n = 4 biological replicates. Statistical significance was calculated by two-way ANOVA with Tukey's multiple comparison test.

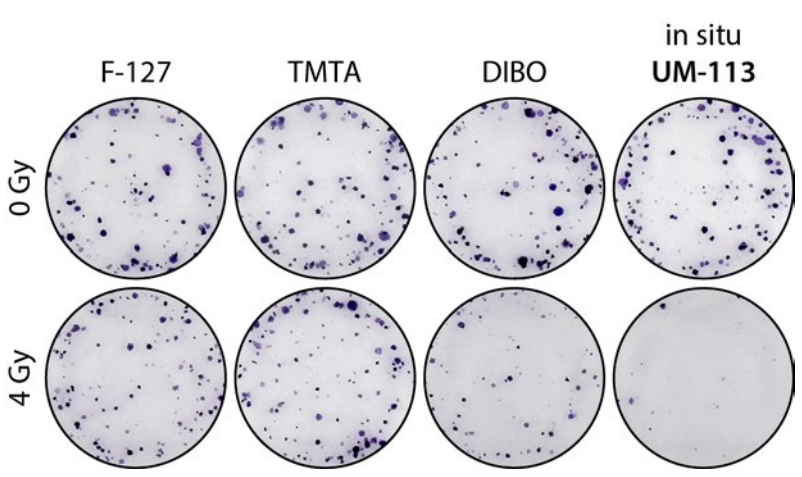

Figure S31. Clonogenic assays of 4T1R cells treated with TMTA (4.0 μM), DIBO (6.0 μM), and Pluronic F-127 (0.04 wt%) for 24 h and exposed to X-ray irradiation (0–4 Gy).

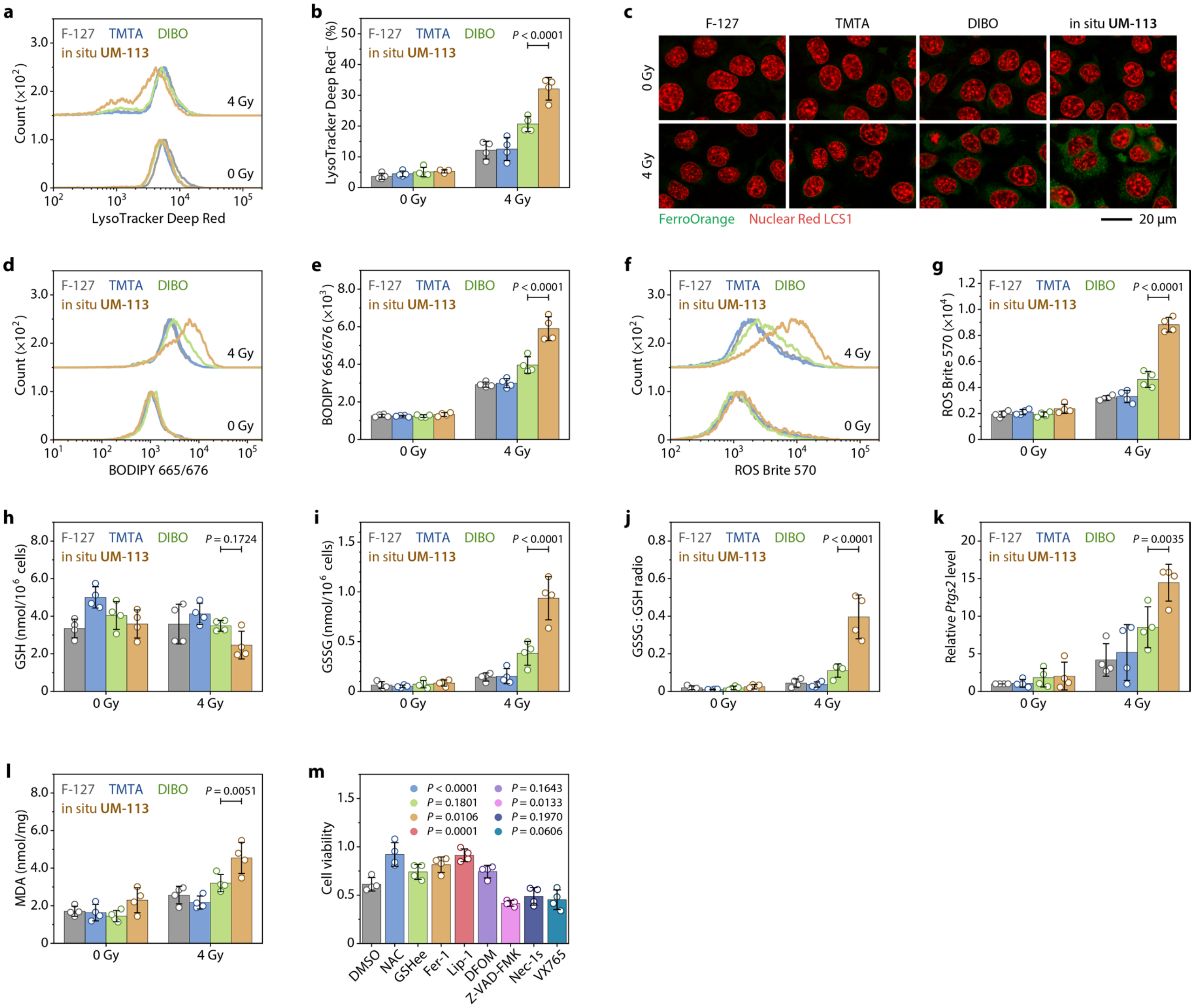

Figure S32. Ferroptosis in vitro. 4T1R cells were treated with TMTA (4.0 μM), DIBO (6.0 μM), and Pluronic F-127 (0.04 wt%) for 24 h and exposed to X-ray irradiation (0–4 Gy). After 24 h of incubation, the cells were used for detecting ferroptosis. (a, b) Flow cytometric analysis (a) and the quantification (b) of LysoTracker Deep Red staining revealing lysosomal alkalisation. (c) Confocal laser scanning fluorescence images of FerroOrange staining for detecting Fe(II). (d, e) Flow cytometric analysis (d) and the quantification (e) of BODIPY 665/676 staining revealing lipid peroxidation. (f, g) Flow cytometric analysis (f) and the quantification (g) of ROS Brite 570 staining for detecting oxidative stress. (h–j) Intracellular GSH (h) and GSSG (i) levels and their ratio (j). (k) Intracellular *Ptgs2* mRNA level. (l) Intracellular MDA level. (m) Cell death rescue experiments in the absence or presence of DMSO (0.1 vol%), NAC (5.0 mM), GSHee (2.0 mM), Fer-1 (1.0 μM), Lip-1 (0.5 μM), DFOM (100 μM), Z-VAD-FMK (25 μM), Nec-1s (5.0 μM), and VX765 (20 μM). Data are expressed as mean ± SD, $n = 4$ biological replicates (b, e, g–m). Statistical significance was calculated by two-way ANOVA followed by Tukey's multiple comparison test (b, e, g–l) and one-way ANOVA followed by Dunnett's multiple comparison test (m).

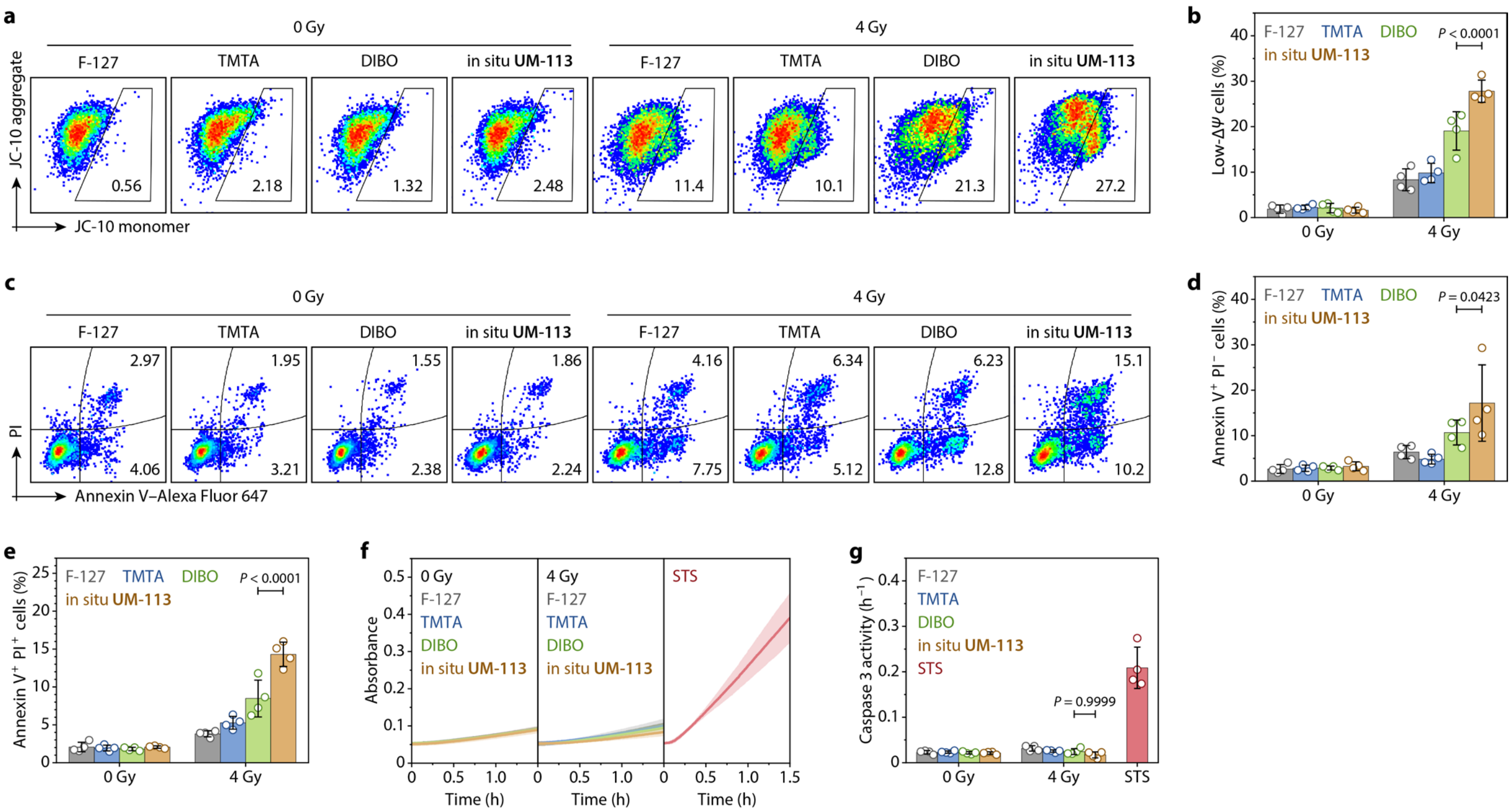

Figure S33. Apoptosis in vitro. 4T1R cells were treated with TMTA (4.0 μM), DIBO (6.0 μM), and Pluronic F-127 (0.04 wt%) for 24 h, followed by exposure to X-ray irradiation (0–4 Gy). After an additional 24 h of incubation, the cells were used for detecting apoptosis. (a, b) Flow cytometric analysis (a) and the quantification (b) of mitochondrial membrane potentials using JC10 staining. (c–e) Flow cytometric analysis (c) and the quantification (d, e) of phosphatidylserine externalization via annexin V–Alexa Fluor 647 staining. (f, g) Kinetic tracking (f) and the quantification (g) of caspase 3 activity in cell lysates. Cells treated with staurosporine (2.0 μM) for 10 h served as the positive control of apoptosis. Data are expressed as mean ± SD, $n = 4$ biological replicates (b, d–g). Statistical significance was calculated by two-way ANOVA followed by Tukey's multiple comparison test (b, d, e, g).

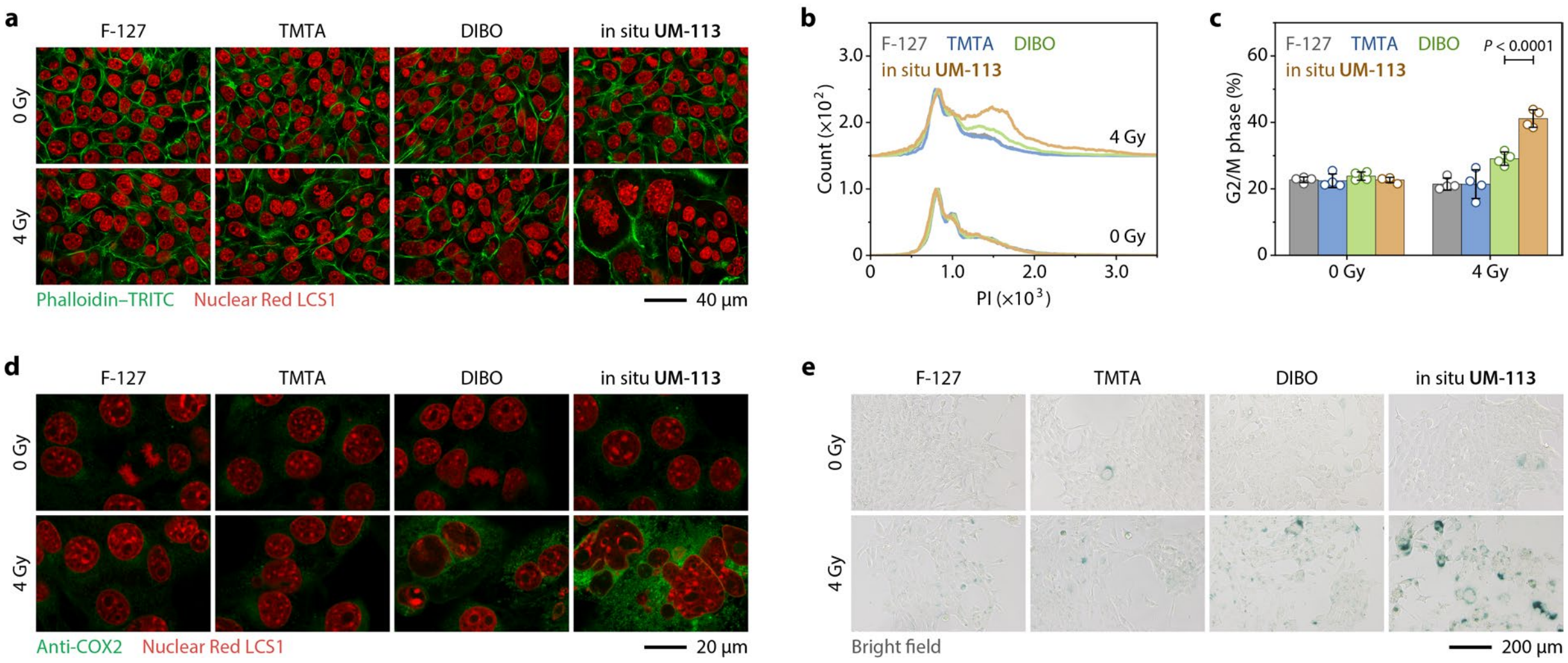

Figure S34. Mitotic catastrophe in vitro. 4T1R cells were treated with TMTA (4.0 μM), DIBO (6.0 μM), and Pluronic F-127 (0.04 wt%) for 24 h, followed by exposure to X-ray irradiation (0–4 Gy). After 72 h of incubation, markers of mitotic catastrophe were assessed. (a) Confocal laser scanning microscopy images showing cytoskeletal morphology stained with phalloidin–TRITC. (b, c) Flow cytometric analysis (b) and quantification (c) of cell cycle distribution based on PI staining. Data are expressed as mean ± SD, $n = 4$ biological replicates. Statistical significance was calculated by two-way ANOVA followed by Tukey's multiple comparison test. (d) Immunofluorescence staining of COX2. (e) Cell senescence-related β-galactosidase staining.

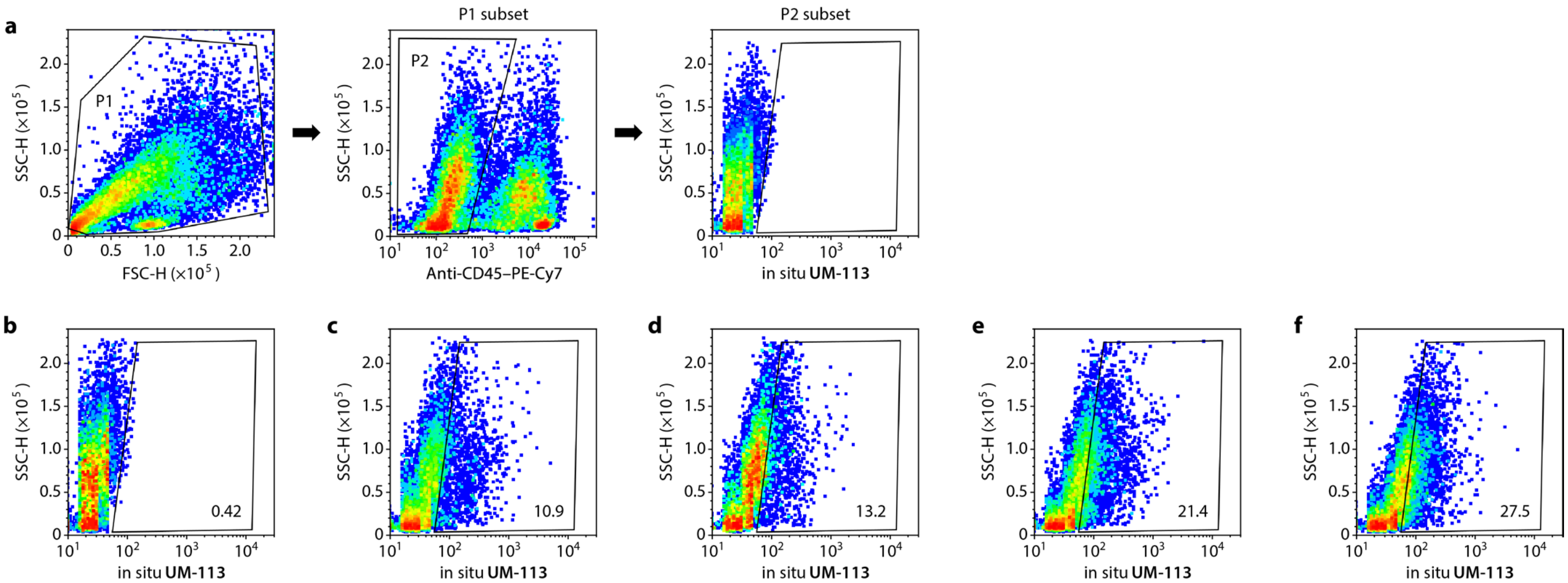

Figure S35. Flow cytometric analysis for in situ **UM-113** synthesis in tumors of 4T1R tumor-bearing BALB/c mice 24 h after receiving different injection doses. (a) Gating strategy. (b–f) Representative flow cytometry plots at injection doses (iodine equiv.) of 0 nmol/mouse (b), 2.5 nmol/mouse (c), 5.0 nmol/mouse (d), 7.5 nmol/mouse (e), and 10 nmol/mouse (f).

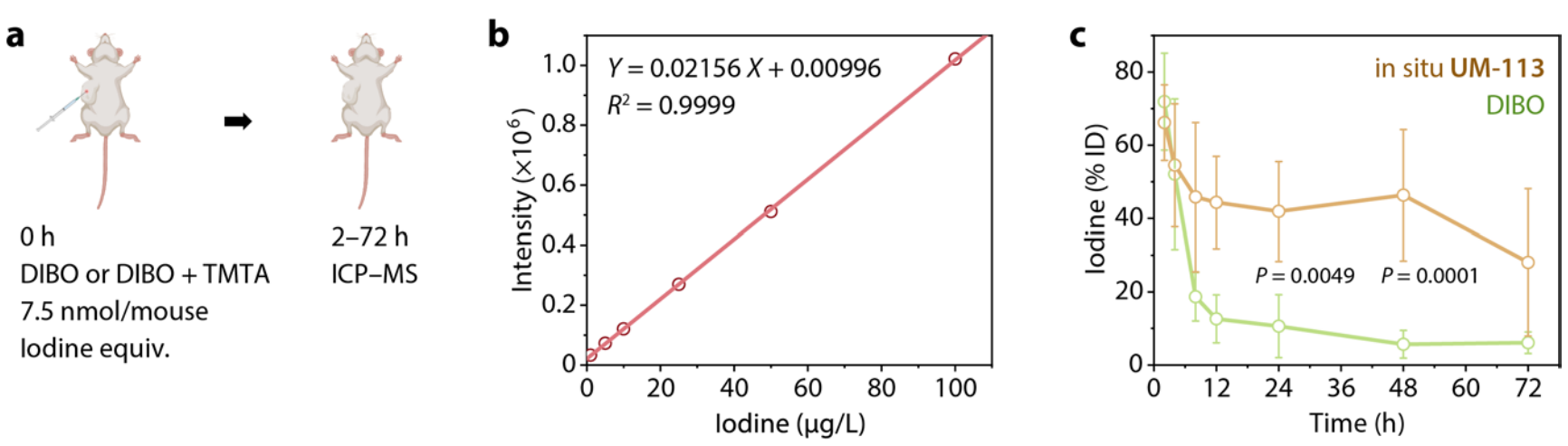

Figure S36. ICP–MS analysis of iodine content in tumors of 4T1R tumor-bearing BALB/c mice at 2–72 h after different treatments. (a) Treatment protocol (created with *BioRender.com*). (b) Standard curve established for ICP–MS analysis. Data are fitted with a linear model. (c) Iodine retention time profile in tumors. Data are expressed as mean ± SD, n = 5 mice per group per time point. Statistical significance was determined using two-way ANOVA with Šídák's multiple comparison test (interaction $p = 0.0034$).

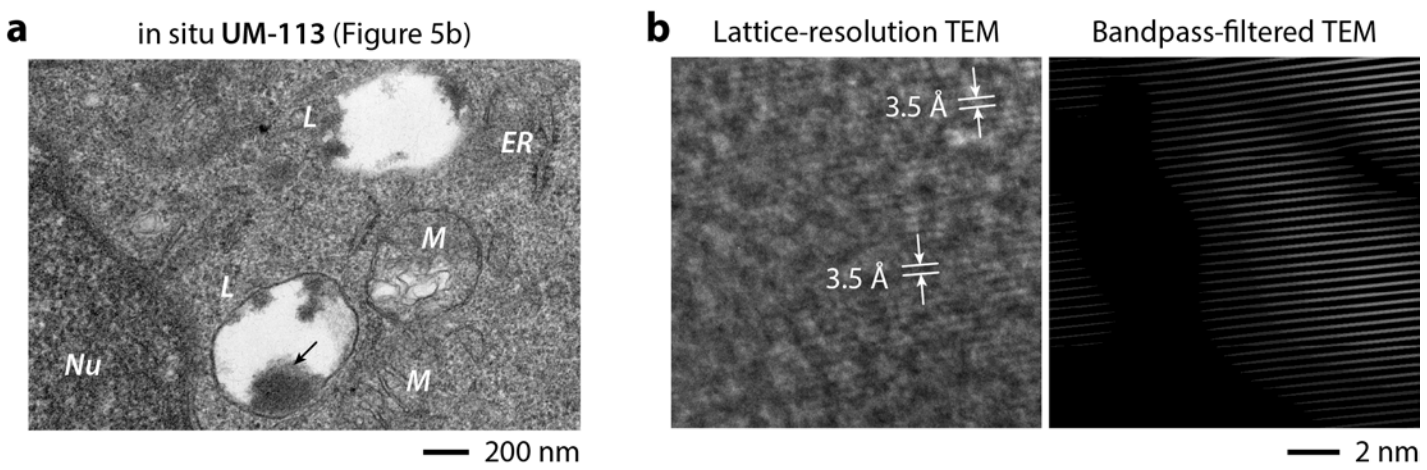

Figure S37. TEM images of tumors showing in situ **UM-113** nanoparticles within lysosomes. (a) Enlarged view of Figure 5b. (b) Lattice-resolution TEM image of in situ **UM-113** nanoparticles inside 4T1R tumors and its bandpass-filtered images with enhanced contrast.

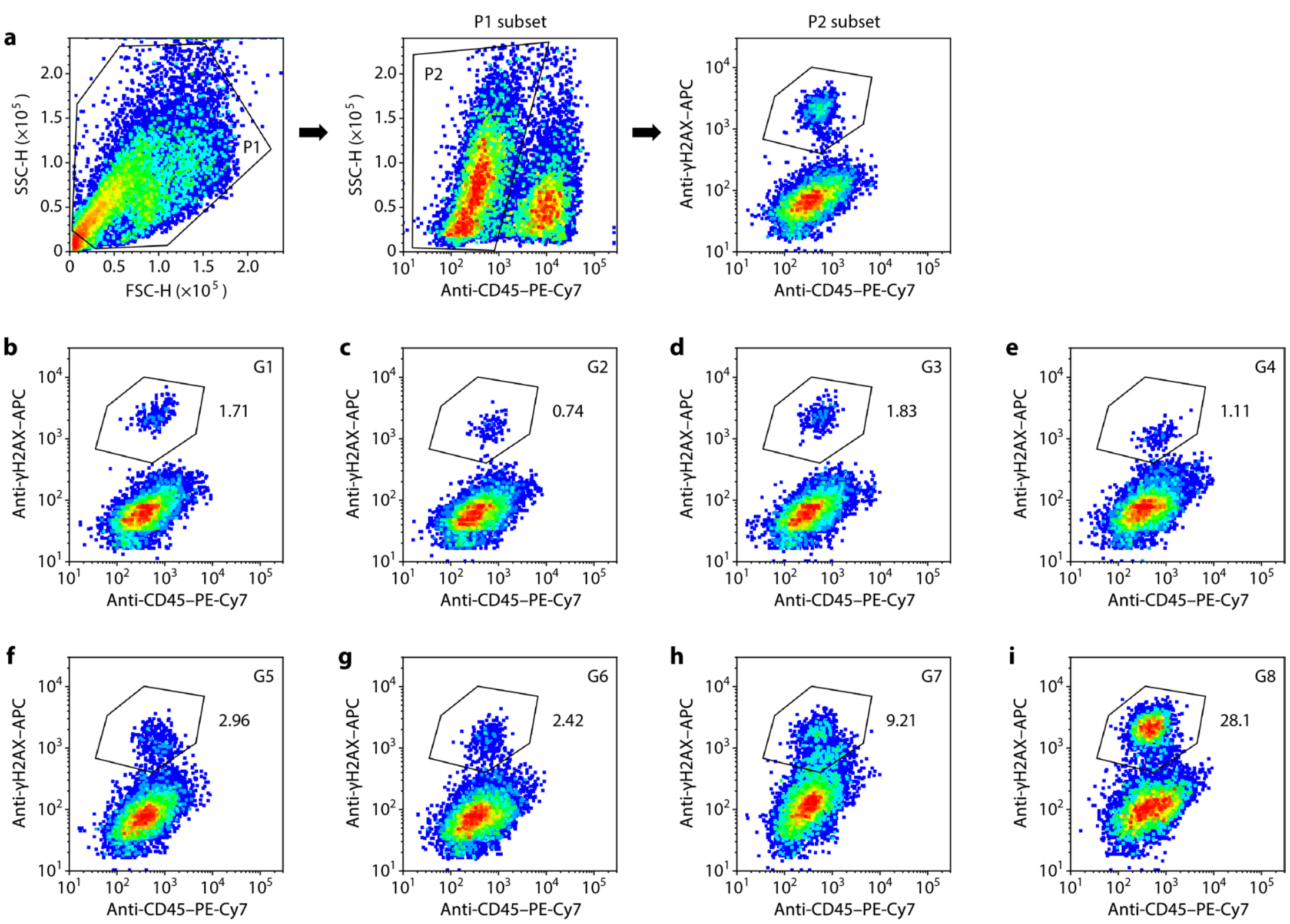

Figure S38. Flow cytometric analysis for γH2AX expression in tumors of 4T1R tumor-bearing BALB/c mice with different treatments. (a) Gating strategy. (b–i) Representative flow cytometric plots of groups G1–G8.

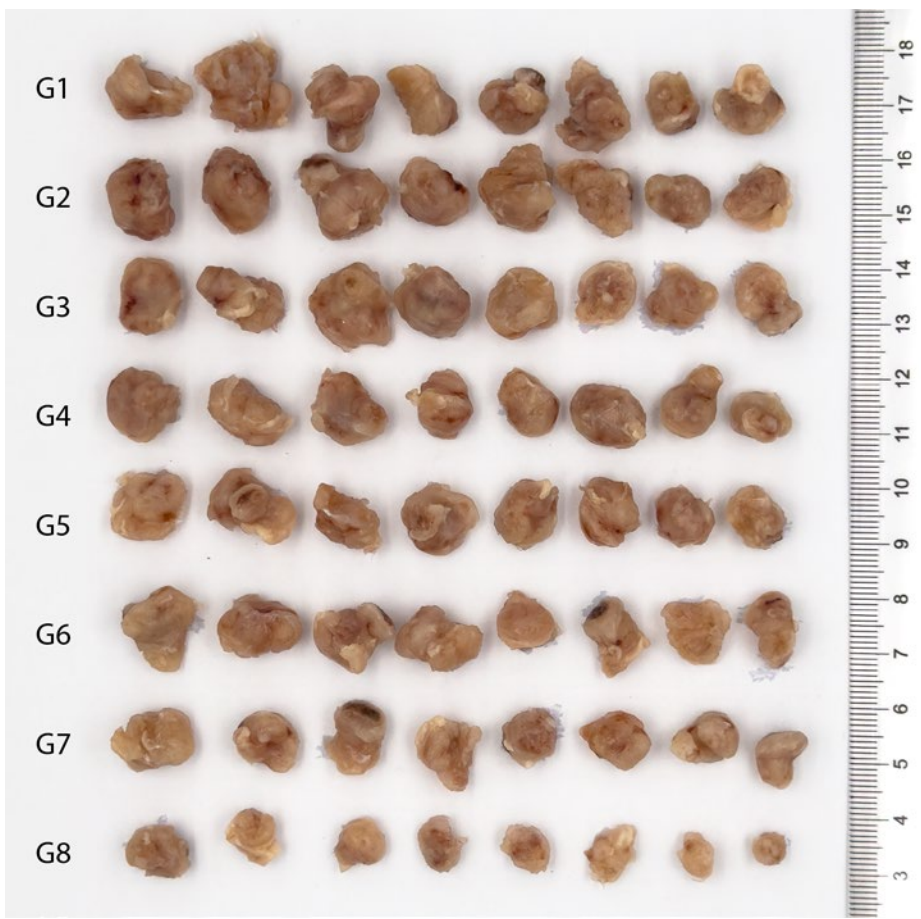

Figure S39. Photograph of tumors excised from mice in groups G1–G8 on day 15, $n = 8$ mice per group.

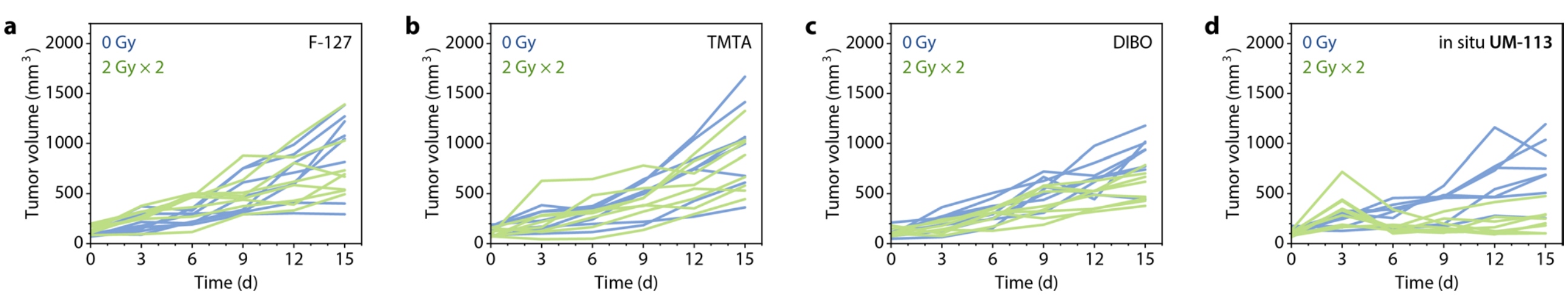

Figure S40. Tumor growth curves of 4T1R tumor-bearing BALB/c mice with different treatments. (a) G1 vs G5. (b) G2 vs G6. (c) G3 vs G7. (d) G4 vs G8.

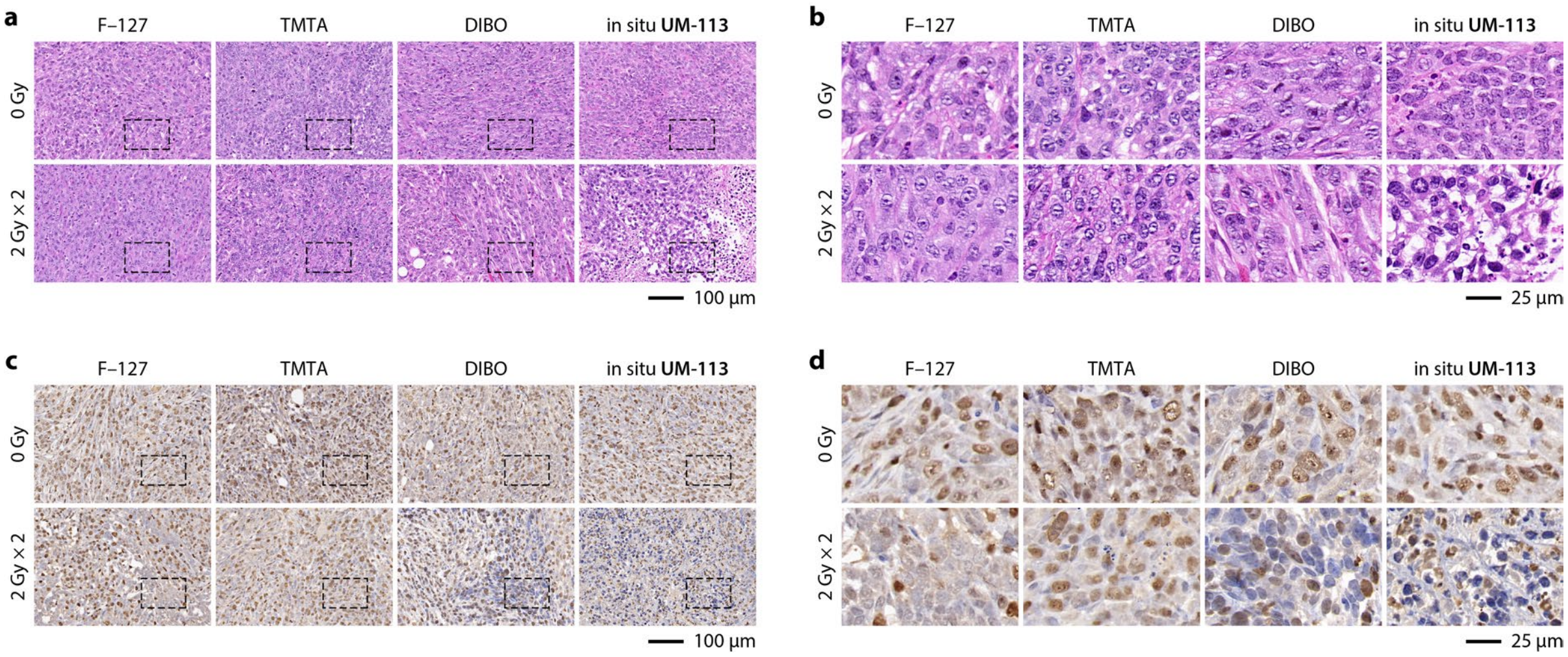

Figure S41. H&E histological analysis (a, b) and Ki67 IHC (c, d) of tumors excised from mice in groups G1–G8 on day 15.

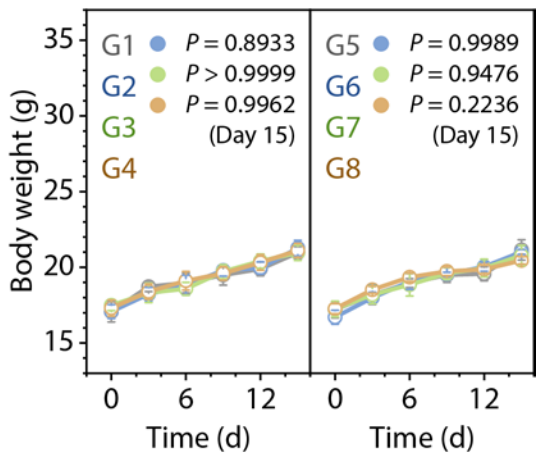

Figure S42. Body weight changes of mice with different treatments. Data are expressed as mean ± SD, n = 8 mice per group. Statistical significance was determined using two-way repeated measures ANOVA with Geisser–Greenhouse correction, followed by Tukey's multiple comparison test.

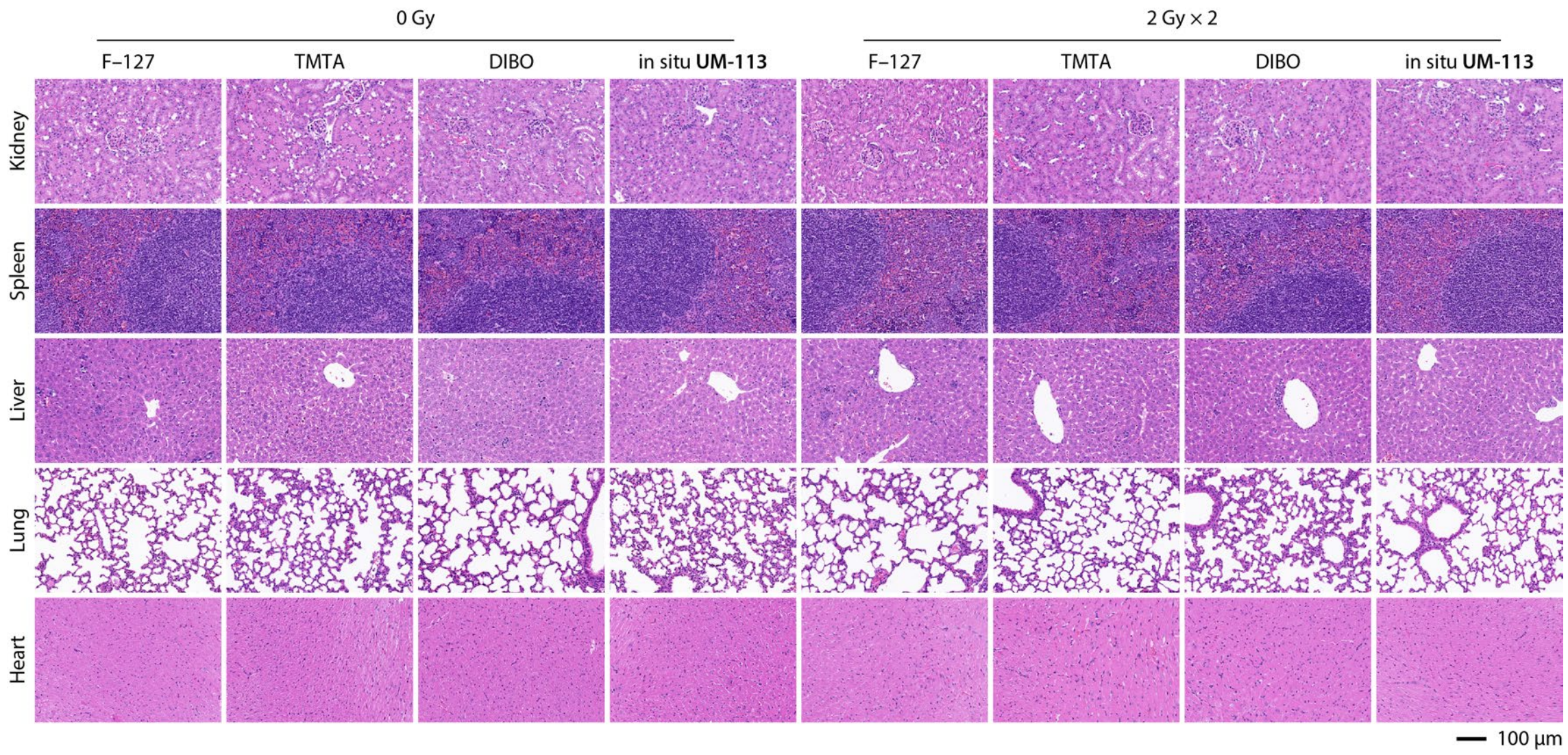

Figure S43. H&E histological analysis of major organs excised from mice in groups G1–G8 on day 15.

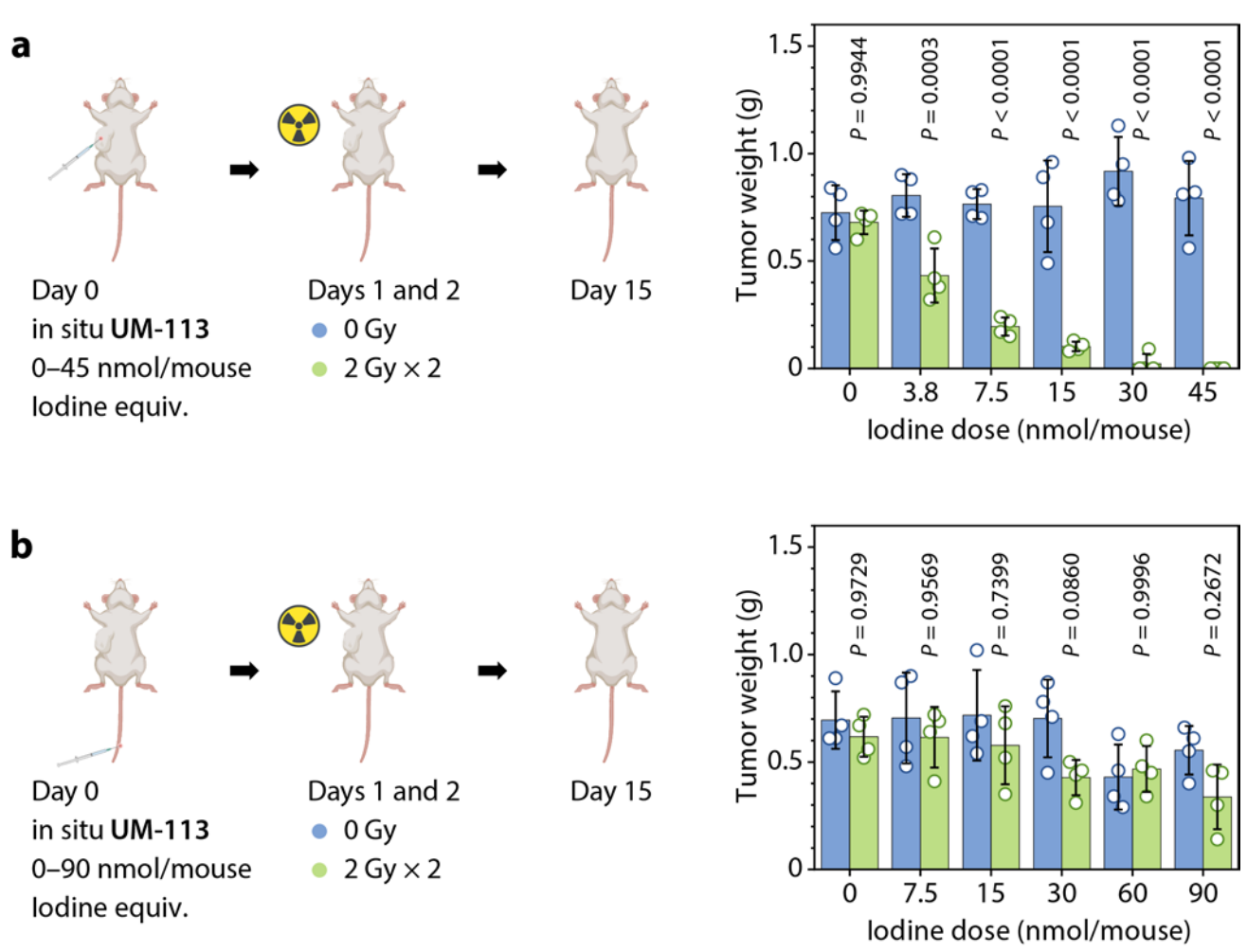

Figure S44. Tumor weights following in situ **UM-113**–enhanced radiotherapy via intratumoral (a) or intravenous (b) injection (created with *BioRender.com*). Data are expressed as mean ± SD, $n = 4$ mice per group. Statistical significance was determined using two-way ANOVA with Šídák's multiple comparison test. Interaction $p < 0.0001$ (a) and $p = 0.3994$ (b).

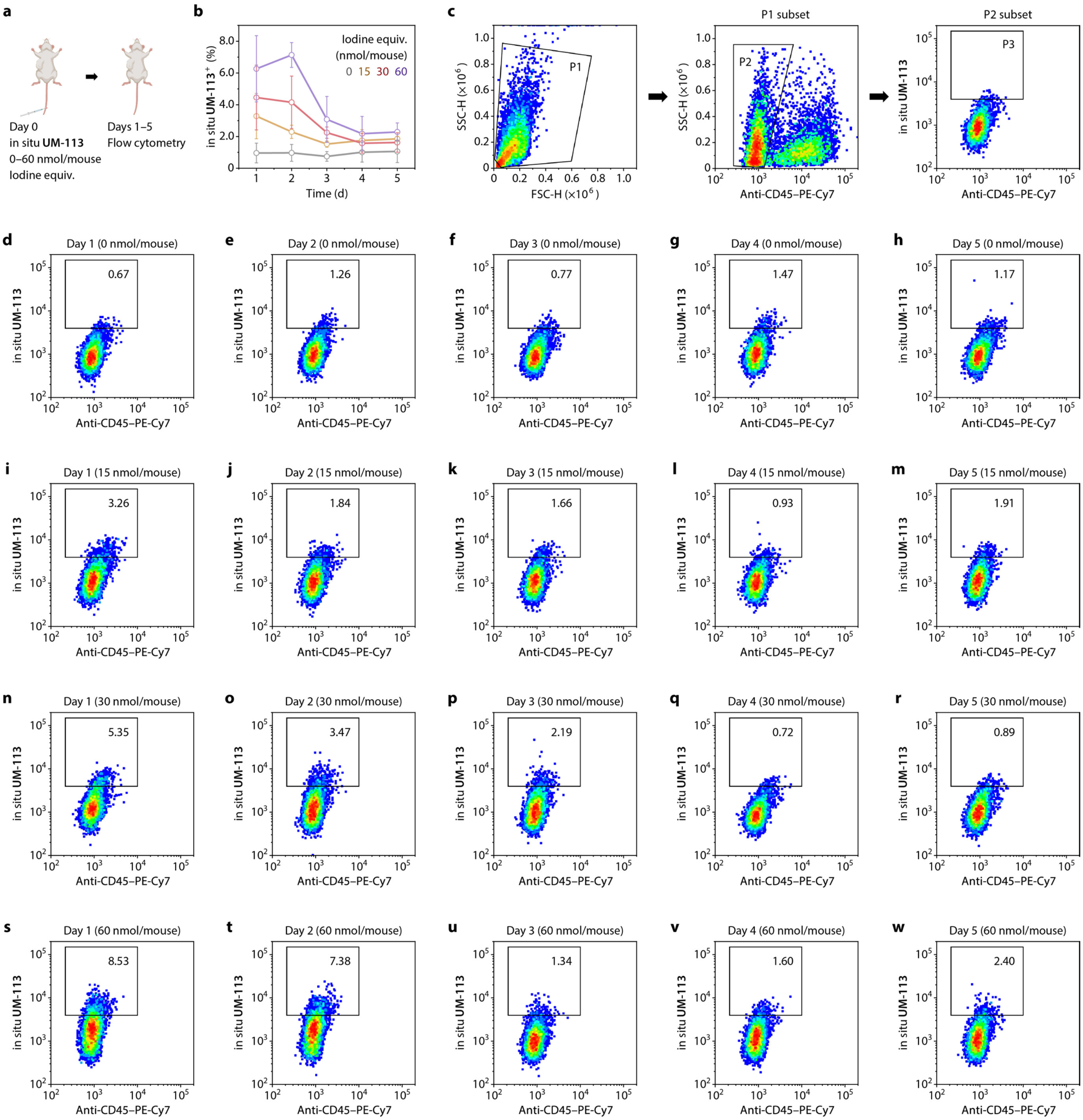

Figure S45. Flow cytometric analysis for in situ **UM-113** synthesis within tumors 1–5 d after intravenous injection of Pluronic F-127, TMTA, and DIBO. (a) Treatment protocol (created with *BioRender.com*). (b) Percentage of in situ **UM-113**-positive cancer cells in tumor tissue. (c) Gating strategy. (d–w) Representative flow cytometry plots at injection doses (iodine equiv.) of 0 (d–h), 15 (i–m), 30 (n–r), and 60 (s–w) nmol/mouse. Data are expressed as mean ± SD, n = 4 mice per group.

Table S1. Atomic coordinates of the AB-stacking mode of **UM-113**.

Atom	*x*/*a*	*y*/*b*	*z*/*c*	Atom	*x*/*a*	*y*/*b*	*z*/*c*
C1	0.31849	−0.37641	0.01365	C25	0.07384	−0.90998	0.02117
C2	0.36168	−0.34828	0.01315	C26	0.11378	−0.88490	0.11508
C3	0.30325	−0.42145	0.02079	C27	0.14060	−0.84274	0.07820
C4	0.39136	−0.36514	0.01335	C28	0.03547	−0.95677	0.05582
C5	0.29747	−0.44384	−0.14970	C29	0.04166	−0.99206	0.05337
C6	0.28227	−0.48657	−0.14263	C30	0.08475	−0.98204	−0.00034
C7	0.27282	−0.50731	0.03608	H31	0.38571	−0.38485	−0.11581
C8	0.27843	−0.48470	0.20561	H32	0.38626	−0.38413	0.14468
C9	0.29373	−0.44200	0.19849	H33	0.42453	−0.34088	0.01075
N10	0.25547	−0.55137	0.05163	H34	0.30435	−0.42825	−0.28781
C11	0.25635	−0.57589	−0.07912	H35	0.27713	−0.50311	−0.27696
C12	0.23534	−0.62118	−0.04492	H36	0.27079	−0.50033	0.34370
C13	0.23871	−0.64815	−0.17724	H37	0.29785	−0.42499	0.33096
C14	0.21726	−0.69096	−0.14355	H38	0.27261	−0.56334	−0.21284
C15	0.19280	−0.70754	0.02200	H39	0.22012	−0.71133	−0.24606
C16	0.18971	−0.68072	0.15506	H40	0.20729	−0.61818	0.22391
C17	0.21071	−0.63807	0.12070	H41	0.16542	−0.76389	0.20263
I18	0.27614	−0.62708	−0.42901	H42	0.08710	−0.83095	−0.27885
I19	0.15150	−0.70253	0.40437	H43	0.04279	−0.90713	−0.19870
C20	0.17071	−0.75258	0.05572	H44	0.12621	−0.89211	0.23390
N21	0.15415	−0.77844	−0.08632	H45	0.16891	−0.82575	0.16124
C22	0.13026	−0.82244	−0.05862	H46	0.08525	−0.97686	−0.14853
C23	0.09481	−0.84575	−0.16479	H47	0.09382	−1.00445	0.05286
C24	0.06869	−0.88798	−0.12056	H48	0.11102	−0.95097	0.02348

*Cell parameters: $a = b = 37.22$ Å, $c = 6.94$ Å, $\alpha = \beta = 90°$, $\gamma = 120°$.

Table S2. Atomic coordinates of the AA'-stacking mode of **UM-114(AA')**.

Atom	*x*/*a*	*y*/*b*	*z*/*c*	Atom	*x*/*a*	*y*/*b*	*z*/*c*	Atom	*x*/*a*	*y*/*b*	*z*/*c*
C1	0.30888	−0.37829	0.25452	C33	0.25182	0.55345	0.63843	H65	0.18996	−0.49791	0.03664
C2	0.35332	−0.35718	0.26124	C34	0.22803	0.51067	0.62740	H66	0.27720	−0.51867	0.33484
C3	0.28218	−0.42636	0.23415	C35	0.24273	0.48632	0.70595	H67	0.31476	−0.44928	0.41447
C4	0.37307	−0.38244	0.31353	C36	0.27884	0.50538	0.80998	H68	0.06859	−0.76425	−0.04246
C5	0.24476	−0.44436	0.13238	C37	0.29847	0.54757	0.84489	H69	0.13379	−0.61924	0.09394
C6	0.21857	−0.48655	0.11353	N38	0.22402	0.44304	0.67119	H70	0.18419	−0.71578	−0.04205
C7	0.23053	−0.51377	0.17782	C39	0.18683	0.41909	0.61144	H71	0.24898	−0.57341	0.14039
C8	0.26789	−0.49816	0.27390	C40	0.17261	0.37550	0.56892	H72	0.03663	−0.83088	0.01003
C9	0.29176	−0.45592	0.31043	C41	0.13094	0.34759	0.52995	H73	0.00535	−0.90384	−0.00346
N10	0.20454	−0.55660	0.13756	C42	0.11854	0.30676	0.48594	H74	0.09676	−0.89084	−0.40416
C11	0.10108	−0.75242	−0.06174	C43	0.14713	0.29299	0.47688	H75	0.13655	−0.82032	−0.32661
C12	0.13044	−0.70883	−0.01922	C44	0.18873	0.32079	0.51769	H76	0.09447	−0.94552	−0.39983
C13	0.11668	−0.68093	0.01974	C45	0.20110	0.36157	0.56250	H77	0.10926	−0.96868	−0.24416
C14	0.14511	−0.64009	0.06376	C46	0.13336	0.24989	0.42581	H78	0.10827	−0.92578	−0.17158
C15	0.18748	−0.62632	0.07282	N47	0.09447	0.22326	0.41214	H79	0.41128	0.63535	0.79386
C16	0.20127	−0.65412	0.03201	C48	0.07763	0.18043	0.37188	H80	0.36738	0.59245	0.72128
C17	0.17286	−0.69490	−0.01280	C49	0.03846	0.15322	0.43617	H81	0.37333	0.61219	0.94953
C18	0.21680	−0.58323	0.12389	C50	0.02246	0.11103	0.41732	H82	0.24252	0.57051	0.55316
N19	0.11431	−0.77638	−0.12149	C51	0.04187	0.09303	0.31555	H83	0.20085	0.49755	0.53967
C20	0.08975	−0.81965	−0.15625	C52	0.08101	0.12259	0.23927	H84	0.29021	0.48699	0.87631
C21	0.05069	−0.84400	−0.07770	C53	0.09938	0.16483	0.27580	H85	0.32094	0.55751	0.95386
C22	0.03170	−0.88679	−0.08873	C54	0.02050	0.04495	0.29518	H86	0.16617	0.43091	0.59216
C23	0.04657	−0.90739	−0.20138	C55	0.04383	0.02384	0.28845	H87	0.08637	0.28591	0.45575
C24	0.08424	−0.88090	−0.29519	C56	0.08884	0.04910	0.23617	H88	0.23331	0.38245	0.59176
C25	0.10680	−0.83871	−0.26028	I57	0.08481	0.36462	0.53009	H89	0.15572	0.24007	0.40931
C26	0.02260	−0.95516	−0.21560	I58	0.23473	0.30356	0.52164	H90	0.02120	0.16457	0.51306
C27	0.04413	−0.97807	−0.21894	I59	0.05352	0.30204	1.01959	H91	−0.00602	0.09289	0.48291
C28	0.09059	−0.95412	−0.25386	I60	0.26450	0.36311	1.02808	H92	0.09737	0.11594	0.13523
C29	0.31109	0.62183	0.76530	H61	0.36468	−0.40909	0.22160	H93	0.12920	0.18534	0.21486
C30	0.35554	0.64474	0.76864	H62	0.36277	−0.39235	0.45452	H94	0.08845	0.05902	0.09518
C31	0.28728	0.57405	0.75107	H63	0.40616	−0.36830	0.33135	H95	0.10779	0.03496	0.21835
C32	0.37804	0.62079	0.80355	H64	0.23442	−0.42623	0.06679	H96	0.10711	0.07576	0.32810

*Cell parameters: $a = b = 37.54$ Å, $c = 7.08$ Å, $\alpha = \beta = 90°$, $\gamma = 120°$.
